# The African cynodont *Aleodon* (Cynodontia, Probainognathia) in the Triassic of southern Brazil and its biostratigraphic significance

**DOI:** 10.1371/journal.pone.0177948

**Published:** 2017-06-14

**Authors:** Agustín G. Martinelli, Christian F. Kammerer, Tomaz P. Melo, Voltaire D. Paes Neto, Ana Maria Ribeiro, Átila A. S. Da-Rosa, Cesar L. Schultz, Marina Bento Soares

**Affiliations:** 1 Laboratório de Paleontologia de Vertebrados, Departamento de Paleontologia e Estratigrafia, Instituto de Geociências, Universidade Federal do Rio Grande do Sul (UFRGS), Agronomia, Porto Alegre, Rio Grande do Sul, Brazil; 2 Museum für Naturkunde, Leibniz-Institut für Evolutions- und Biodiversitätsforschung, Berlin, Germany; 3 Seção de Paleontologia, Museu de Ciências Naturais da Fundação Zoobotânica do Rio Grande do Sul, Porto Alegre, Rio Grande do Sul, Brazil; 4 Laboratório de Estratigrafia e Paleobiologia, Departamento de Geociências, Universidade Federal de Santa Maria, Santa Maria, Rio Grande do Sul, Brazil; University of Michigan, UNITED STATES

## Abstract

In this contribution we report the first occurrence of the enigmatic African probainognathian genus *Aleodon* in the Middle-early Late Triassic of several localities from the state of Rio Grande do Sul in southern Brazil. *Aleodon* is unusual among early probainognathians in having transversely-expanded postcanine teeth, similar to those of gomphodont cynognathians. This genus was previously known from the Manda Beds of Tanzania and the upper Omingonde Formation of Namibia. The Brazilian record of this genus is based upon multiple specimens representing different ontogenetic stages, including three that were previously referred to the sectorial-toothed probainognathian *Chiniquodon theotonicus*. We propose a new species of *Aleodon* (*A*. *cromptoni* sp. nov.) based on the specimens from Brazil. Additionally, we tentatively refer one specimen from the upper Omingonde Formation of Namibia to this new taxon, strengthening biostratigraphic correlations between these strata. Inclusion of *A*. *cromptoni* in a phylogenetic analysis of eucynodonts recovers it as the sister-taxon of *A*. *brachyrhamphus* within the family Chiniquodontidae. The discovery of numerous specimens of *Aleodon* among the supposedly monospecific *Chiniquodon* samples of Brazil raises concerns about chiniquodontid alpha taxonomy, particularly given the extremely broad geographic distribution of *Chiniquodon*. The discovery of Brazilian *Aleodon* and new records of the traversodontid *Luangwa* supports the hypothesis that at least two subzones can be recognized in the *Dinodontosaurus* Assemblage Zone.

## Introduction

The South American fossil record of non-mammalian eucynodonts is one of the most diverse known worldwide [1–3] and includes representatives of most of the major Triassic subclades (e.g., cynognathids, gomphognathids, traversodontids, chiniquodontids, ecteniniids, probainognathids, ictidosaurs, and mammaliaforms; for review see [1, 4]). While most South American Triassic eucynodont species appear to be local endemics, several have trans-basinal distributions that are useful for strengthening biostratigraphic correlations both among South American basins and with other rock units globally. Notable examples of South American taxa with trans-Gondwanan distributions include the cynognathian species *Cynognathus crateronotus* and *Diademodon tetragonus* from the Middle Triassic of southern Africa, Antarctica, and Argentina [5–10] (see also Discussion), the traversodontid genus *Luangwa* from the late Middle Triassic of Brazil, Namibia, and Zambia [11–13], the chiniquodontid genus *Chiniquodon* from the late Middle to middle Late Triassic of Argentina, Brazil, Namibia, and Madagascar [13–15], and the traversodontid species *Menadon besairiei* from the Late Triassic of Brazil and Madagascar [16–18].

**Fig 1:**
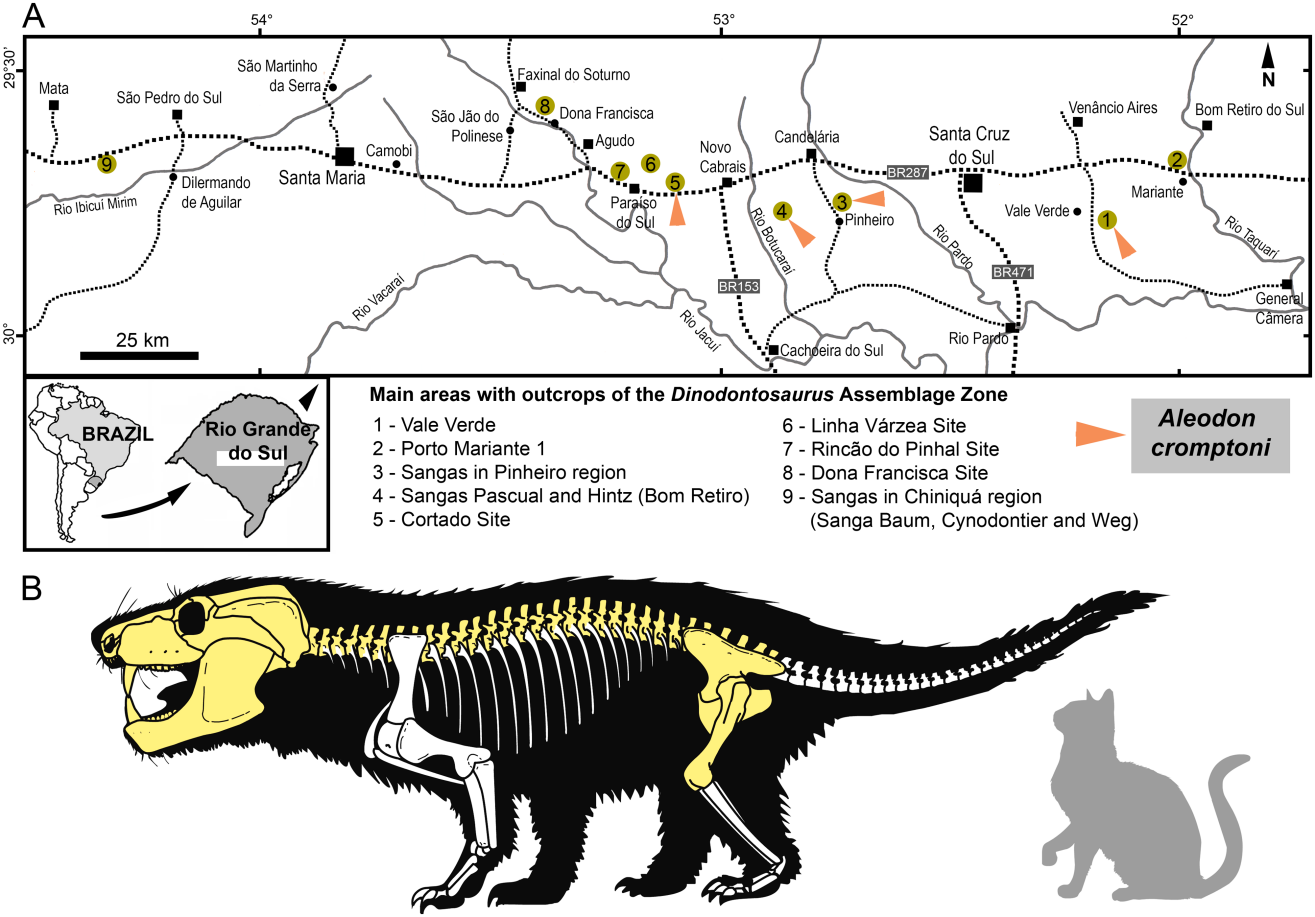
Localities with *Aleodon cromptoni* sp. nov. Map of the main Middle to early Upper Triassic outcrops in the state of Rio Grande do Sul, southern Brazil, highlighting the occurrence of *Aleodon* (A) and its skeletal reconstruction with available bones (in yellow) based on all known specimens (made by VDPN).

In this contribution we report the first occurrence of the enigmatic African probainognathian *Aleodon* in the Middle-early Late Triassic of several localities from the state of Rio Grande do Sul in southern Brazil (Fig 1). *Aleodon* is unusual among early probainognathians in having transversely-expanded postcanines, similar to those of gomphodont cynognathians. The new record is based upon multiple specimens, including three that were previously referred to the sectorial-toothed probainognathian *Chiniquodon* [14, 19]. We propose a new species of *Aleodon* based on the new specimens from Brazil (Fig 1) and discuss its biostratigraphic importance, as well as commenting on other Middle to Late Triassic faunal associations in South America.

### *Aleodon* crompton 1955 –Background

*Aleodon brachyrhamphus* was initially described by Crompton [20] based on a partial skull and lower jaws (the holotype, UMZC T906) collected by F. R. Parrington in 1933 in the Lifua Member of the Manda Beds of Tanzania [21]. The holotype was found at locality B29 of Stockley [22], located between Gingama and Tschikongo in the southwestern portion of the Ruhuhu Valley. Crompton [20] originally described *Aleodon* as a gomphodont cynodont, and diagnosed it as a new genus based on the possession of a short snout and only two lower incisors. Referral to Gomphodontia was based primarily on the presence of three types of postcanines, including circular anterior, transversely expanded, ovate middle, and sectorial posterior teeth, similar to the postcanine pattern of the well-known gomphodont *Diademodon tetragonus* [23–24].

Hopson and Kitching [25] disputed the gomphodont affinities of *Aleodon* based on unpublished specimens with less worn dentition, and transferred *Aleodon* to the Chiniquodontidae. This proposal was accepted in the majority of subsequent studies (e.g., [26–28]). In their revision of the Chiniquodontidae, however, Abdala and Giannini [14] excluded *Aleodon* from the family because of well-developed lingual cingular platforms on the postcanines, which are supposed to be absent in *Chiniquodon*. They also noted that the majority of *Aleodon* specimens from the Manda Beds, such as those housed in the collections of the Natural History Museum in London, are currently undescribed [14] and that further study is necessary to elucidate the phylogenetic position of this taxon. More recently, Abdala and Smith [13] described a partial skull with poorly preserved dentition as *Aleodon* sp. from the upper Omingonde Formation of Namibia, extending the geographic distribution of the genus. They also provided an emended diagnosis of the species *A*. *brachyrhamphus*. Finally, Sidor et al. ([29]; see also [30–31]) briefly reported on new finds from the Manda Beds of Tanzania, including multiple more complete specimens of *A*. *brachyrhamphus* currently awaiting description.

Compared to coeval cynodonts known from extensive cranial material, *Aleodon brachyrhamphus* has been included in relatively few analyses of phylogenetic relationships. Hopson and Kitching [32] were the first to include *A*. *brachyrhamphus* in a phylogenetic analysis, followed by Sidor [28] and Ruta et al. [33]. All of these analyses recovered *Aleodon* as the sister taxon of *Chiniquodon*, with this clade nested between *Lumkuia*/*Ecteninion* and the large clade containing *Probainognathus* plus other, more crownward probainognathians.

## Materials and methods

### Access to specimens

The specimens described here (Table 1) and those used for comparison belong to public collections and were examined with the explicit permission of appropriate curators and/or collection managers (see Acknowledgments). Repository locations and abbreviations for all specimens cited in the text are listed below. We followed all Brazilian regulations for fossil studies and we complied with the PLoS Paleontological Ethics Statement.

**Table 1:** List of specimens referred to *Aleodon cromptoni* sp. nov. from the *Dinodontosaurus* Assemblage Zone, state of Rio Grande do Sul, Brazil.

Specimen	Locality	Original assignment	Main references
MPDC-501-117	Vale Verde	Traversodontidae indet.	Museum specimen catalog
UFRGS-PV-0071-T	Sanga Pinheiro	*Chiniquodon*	Museum specimen catalog
UFRGS-PV-0122-T	Sanga Pinheiro	*Chiniquodon theotonicus*	Abdala and Giannini [14]
UFRGS-PV-0125-T	Sanga Nicanor	*Chiniquodon*	Museum specimen catalog
MMACR-PV-018-T	Sanga Nicanor	*Chiniquodon*	Museum specimen catalog
UFRGS-PV-0146-T	Sanga Pascual	*Chiniquodon* cf. *C*. *theotonicus*	Oliveira et al. [19]
MCN-PV 10338	Cortado site	*Chiniquodon*	Museum specimen catalog
MCP-PV-1695T	Porto Mariante 2	*Chiniquodon*	Museum specimen catalog
UFRGS-PV-0274-T	Exact locality unknown	*Chiniquodon theotonicus*	Abdala and Giannini [14]

### Materials

**Fig 2:**
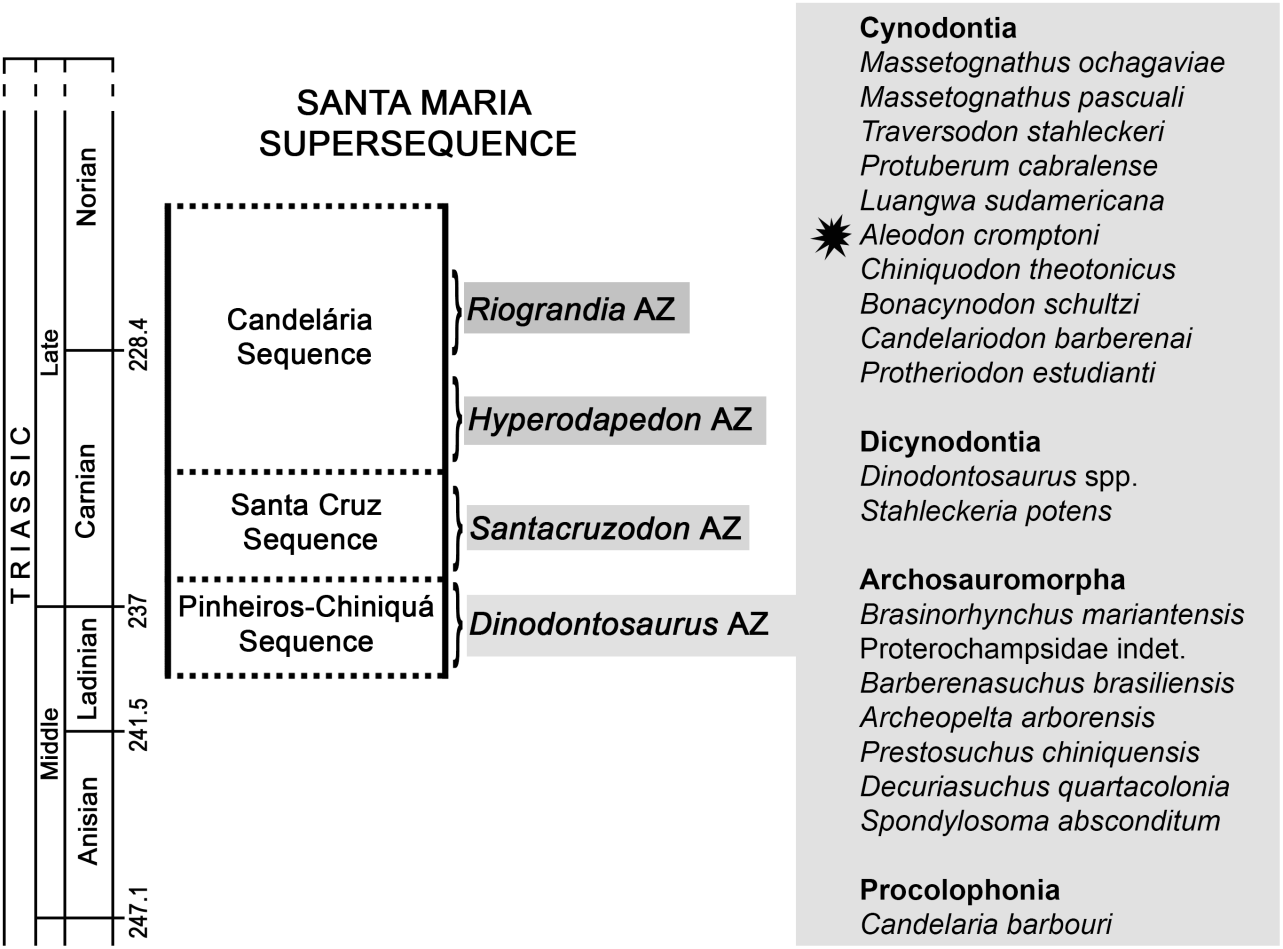
Chrono- and biostratigraphy of Triassic units with vertebrate Assemblages Zones (AZ) from southern Brazil, and the tetrapod fossil content of the *Dinodontosaurus* AZ. The ages of the column follow Gradstein et al. [36].

Cynodont specimens from the *Dinodontosaurus* Assemblage Zone (AZ; Fig 2) [34–35] of southern Brazil were examined firsthand by the lead author, leading to the discovery of eight specimens referable to *Aleodon*. Comparisons with the type species *A*. *brachyrhamphus* were based on direct examination of specimens housed at the UMZC (Cambridge) and NHMUK (London). Measurements of the *Aleodon* specimens from Brazil are detailed in Table 2.

**Table 2:** Measurements (in mm) of *Aleodon cromptoni* sp. nov. specimens from the Middle-early Late Triassic of southern Brazil. Measurement parameters were taken from Abdala and Giannini [14]. *Estimated measure.

**MPDC-Holotype 501–117, right maxilla**														
	Canine	PC1	PC2	PC3	PC4	PC5		PC6	PC7					
Mesio-distal length	12	2.5	3	5	5.5	5.5		6.2	6.5					
Labio-lingual length	8.3	3	4	6.2	8.2	7.9		8.6	9.6					
Upper postcanine row length	34.5													
**UFRGS-PV-071T, snout**														
Muzzle length	56.8													
Upper postcanine row length	31													
Maxillary bicanine width	42.4													
Palate length	58.6													
Anterior postcanine distance	17*													
Posterior postcanine distance	43*													
Canine mesio-distal length (right)	8.7													
Canine labio-lingual length (right)	6.1													
**MMACR-PV-018T, left maxilla**														
Upper postcanine row length (9 alveoli)	32.1*													
Canine mesio-distal length	8.9													
Canine labio-lingual length	6													
**UFRGS-PV-0125T, skull**														
Total skull length	226													
Maxillary bicanine width	74.4													
Muzzle length	86													
Temporal region length	88.4													
Palate length	97													
Zygomatic height	60.2													
**MCN-PV-10338, lower jaws**														
Left side	**c**	**pc1**	**pc2**	**pc3**	**pc4**		**pc5**	**pc6**	**pc7**	**pc8**	**pc9**	**pc10**	**pc11**	**pc12**
Mesio-distal length	16	4	_	4.1	4.7		_	5.2	5.5	7.4	8.5	7.8	8	8.5
Labio-lingual length	11	3	_	3.5	5.3		_	5.7	5.5	5.2	5.3	5.6	6.2	7
Lower postcanine row length	66.1													
**UFRGS-PV-0146T, skull and jaws**														
Total skull length	236													
Maxillary bicanine width	65.3													
Muzzle length	87.2													
Temporal region length	107													
Palate length	117.2													
Zygomatic height	78													
Upper canine mesio-distal length (left)	21.4													
Upper canine labio-lingual length (left)	11.6													
Mandibular lenght (left)	211													
Lower canine mesio-distal length (right)	18													
Lower canine labio-lingual length (right)	11													
**UFRGS-PV-0274T, skull**														
Total skull length	277													
Maxillary bicanine width	123													
Muzzle length	127													
Temporal region length	107													
Palate length	133													
Upper canine mesio-distal length (left)	22													
Upper canine labio-lingual length (left)	14													
**UFRGS-PV-0122T, skull and jaws**														
Total skull length	310													
Muzzle length	126.3													
Temporal region length	147.8													
Palate length	146.4													
Zygomatic heigth	93													
Upper canine mesio-distal length (right)	23.9													
Upper canine labio-lingual length (right)	17.4													
Mandibular length (left)	274													

The database of localities and tetrapod species from the *Dinodontosaurus* AZ (Fig 2) was based on the literature (see S1 File) and personal observation of various specimens (other than probainognathians), particularly those deposited in institutions in Rio Grande do Sul (e.g., MCN-PV, MMACR-PV-T, MPDC, UFRGS-PV-T, UFSM; see Institutional Abbreviations). For the study of similarities among localities we used the Jaccard Similarity Coefficient to construct the dendrograms, using the software Past v3.13 [37]. The GPS coordinates of the mentioned *Dinodontosaurus* AZ localities are available by contacting the corresponding author.

### Phylogenetics

The new species named herein (*Aleodon cromptoni*) was included in the phylogenetic data matrix of Ruta et al. [33], which includes 54 cynodont operational taxonomic units (including *A*. *cromptoni*) and 150 discrete cranial, dental, and postcranial characters. Modifications of character-states of some taxa (i.e., *Chiniquodon*, *Aleodon brachyrhamphus*, *Probainognathus*) and the complete data matrix are available in the S1 and S2 Files.

The modified data matrix (S2 File) was analyzed under equally-weighted parsimony using TNT 1.5 [38]. A heuristic search of 500 replicates of Wagner trees, followed by TBR branch-swapping algorithm (holding 10 trees per replication), was performed. All characters were treated as non-additive. Bremer support [39] and bootstrap resampling analysis [40] were conducted.

### Nomenclatural acts

The electronic edition of this article conforms to the requirements of the amended International Code of Zoological Nomenclature, and hence the new names contained herein are available under that Code from the electronic edition of this article. This published work and the nomenclatural acts it contains have been registered in ZooBank, the online registration system for the ICZN. The ZooBank LSIDs (Life Science Identifiers) can be resolved and the associated information viewed through any standard web browser by appending the LSID to the prefix http://zoobank.org/. The LSID for this publication is: urn:lsid:zoobank.org:pub:BFF11AF1-742F-4446-9871-3908F70F2E93. The electronic edition of this work was published in a journal with an ISSN, and has been archived and is available from the following digital repositories: PubMed Central and LOCKSS.

### Institutional Abbreviations

**GPIT**, Institut und Museum für Geologie und Paläontologie der Universität Tübingen, Tübingen, Germany; **GSN**, Geological Survey of Namibia, Windhoek, Namibia; **MCN-PV**, Museu de Ciências Naturais (Paleovertebrate Collection), Fundação Zoobotânica do Rio Grande do Sul, Porto Alegre, Brazil; **MCP-PV**, Museu de Ciências e Tecnologia (Paleovertebrate Collection), Pontifícia Universidade Católica do Rio Grande do Sul, Porto Alegre, Brazil; **MCT**, Museu de Ciências da Terra, Rio de Janeiro, Brazil; **MMACR-PV-T**, Museu Municipal Aristides Carlos Rodrigues (Paleovertebrates-Triassic Collection), Candelária, Rio Grande do Sul, Brazil; **MPDC**, Museu Padre Daniel Cargnin, Mata, Rio Grande do Sul, Brazil; **NHMUK** (ex BMNH), Natural History Museum (PV, Vertebrate Paleontology; R, Reptiles), London, United Kingdom; **PVL**, Instituto Miguel Lillo, Universidad Nacional de Tucumán (Paleovertebrate Collection), San Miguel de Tucumán, Tucumán, Argentina; **UFRGS-PV-T**, Universidade Federal Rio Grande do Sul (Paleovertebrate, Triassic Collection), Porto Alegre, Brazil; **UFSM**, Laboratório de Estratigrafia e Paleobiologia, Universidade Federal de Santa Maria, Santa Maria, Brazil; **UMZC**, University Museum of Zoology, Cambridge, United Kingdom.

### Anatomical Abbreviations

A, main upper cusp A; a-b-c-d, lower cusps on crown; a1-4, alveoli for incisors 1 to 4; a1-9, alveoli for postcanines 1 to 9; a8, alveolus for lower postcanine 8; ac, alveolus for lower canine; ai3, alveolus incisor for i3; bs/ps basisphenoid/parasphenoid; C, upper canine; aPC, alveolus for upper postcanine teeth; c, lower canine; Ca, upper canine alveolus; co, condyle of exoccipital; Cr, upper canine root; cre, canine reconstruction; d, dentary; de, dentine; di, diastema; eal, empty alveolus; enl, enamel layer; fpal, facet for palatine; fr, frontal; gr, groove; I, upper incisor; i, lower incisor; in, interlocking; ju, jugal; lae, labial edge; lie, lingual edge; lPC, last upper postcanine tooth; lpl, lingual platform; max, maxilla; mpm, medial process of maxilla; na, nasal; pa, parietal; pal, palatine; PC, upper postcanine; pc, lower postcanine; pdc, postdentary bone complex; pf, paracanine fossa; pfr, prefrontal; pm, premaxilla; po, postorbital; pp, postparietal; pt, pterygoid; r, root; ral, resorbed alveoli; sm, septomaxilla; spl, splenial; sq, squamosal; v, vomer.

## Results

### Systematic Paleontology

Therapsida Broom, 1905

Cynodontia Owen, 1861

Eucynodontia Kemp, 1982

Probainognathia Hopson, 1990

*Aleodon* Crompton, 1955

#### Type species

*Aleodon brachyrhamphus* Crompton, 1955

#### Diagnosis

Probainognathian cynodonts characterized by the following combination of features (autapomorphies denoted by an asterisk): dental formula of I4/i3, C1/c1, PC9–11/pc10–12, long osseous secondary palate extending to the posterior end of the tooth row; dorsoventrally deep zygomatic arch formed by jugal and squamosal; strongly labially concave upper postcanine tooth row; upper and lower postcanines ovoid to ellipsoid in outline in occlusal view, bearing a sectorial labial margin with two to three cusps in line and a well-developed lingual platform (more expansive than the lingual cingulum of other basal probainognathians) without a transverse crest*; cusps of upper and lower postcanines less recurved posteriorly and apicobasally shorter than in *Chiniquodon* species.

*Aleodon brachyrhamphus* Crompton, 1955

#### Holotype

UMZC T906, partial skull and lower jaws from the Ruhuhu Valley, Tanzania [20]. Lifua Member, Manda Beds, Ruhuhu Basin [21].

#### Emended diagnosis

Distinguished (autapomorphies denoted by an asterisk) from its congener *A*. *cromptoni* sp. nov. by a gradual increase in upper postcanine size between PC1 and PC4; mesiodistally narrower lingual platform of the upper and lower postcanines*; relatively labiolingually elongate upper and lower postcanine crowns, with the occlusal surface of the uppers notably constricted between the labial cusp and the lingual platform*; slightly concave mesial edge and slightly convex distal edge of middle and posterior lower postcanines; enlargement of canine-postcanine diastema during growth and, consequently, fewer upper and lower postcanines at adult size.

*Aleodon cromptoni* sp. nov.

urn:lsid:zoobank.org:act:2AC78692-01AC-4CE1-8E39-3349B14214F6

#### Holotype

**Fig 3:**
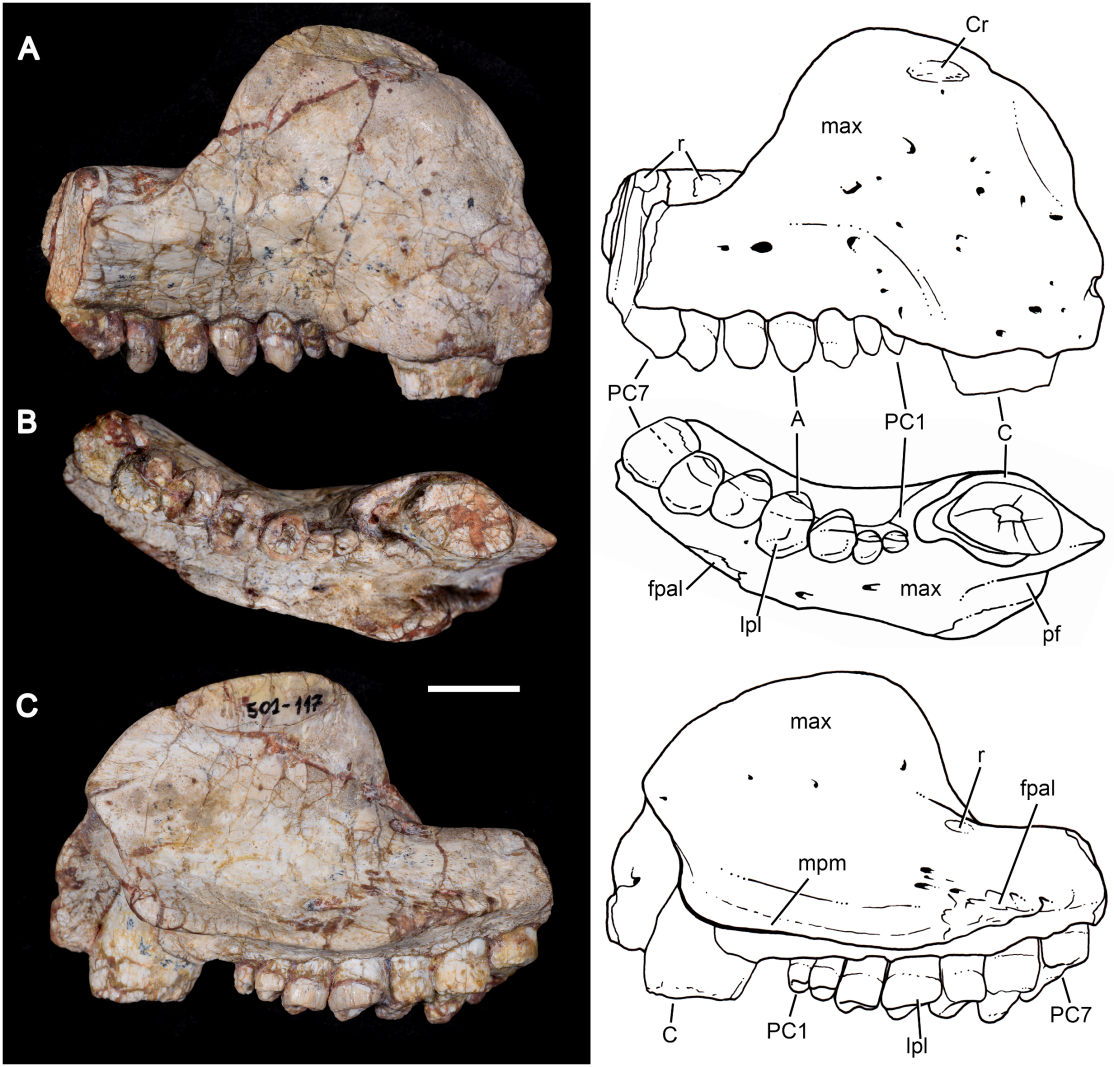
*Aleodon cromptoni* sp. nov. from southern Brazil. Photographs and accompanying drawings of right maxilla MPDC-501-117 in lateral (A), ventral (B), and medial views (C). Scale bar equals 10mm. Abbreviations: A, main cusp A; C, upper canine; Cr, canine root; fpal, facet for palatine; lpl, lingual platform; max, maxilla; mpm, medial process of maxilla; PC, upper postcanine; pf, paracanine fossa; r, root.

**Fig 4:**
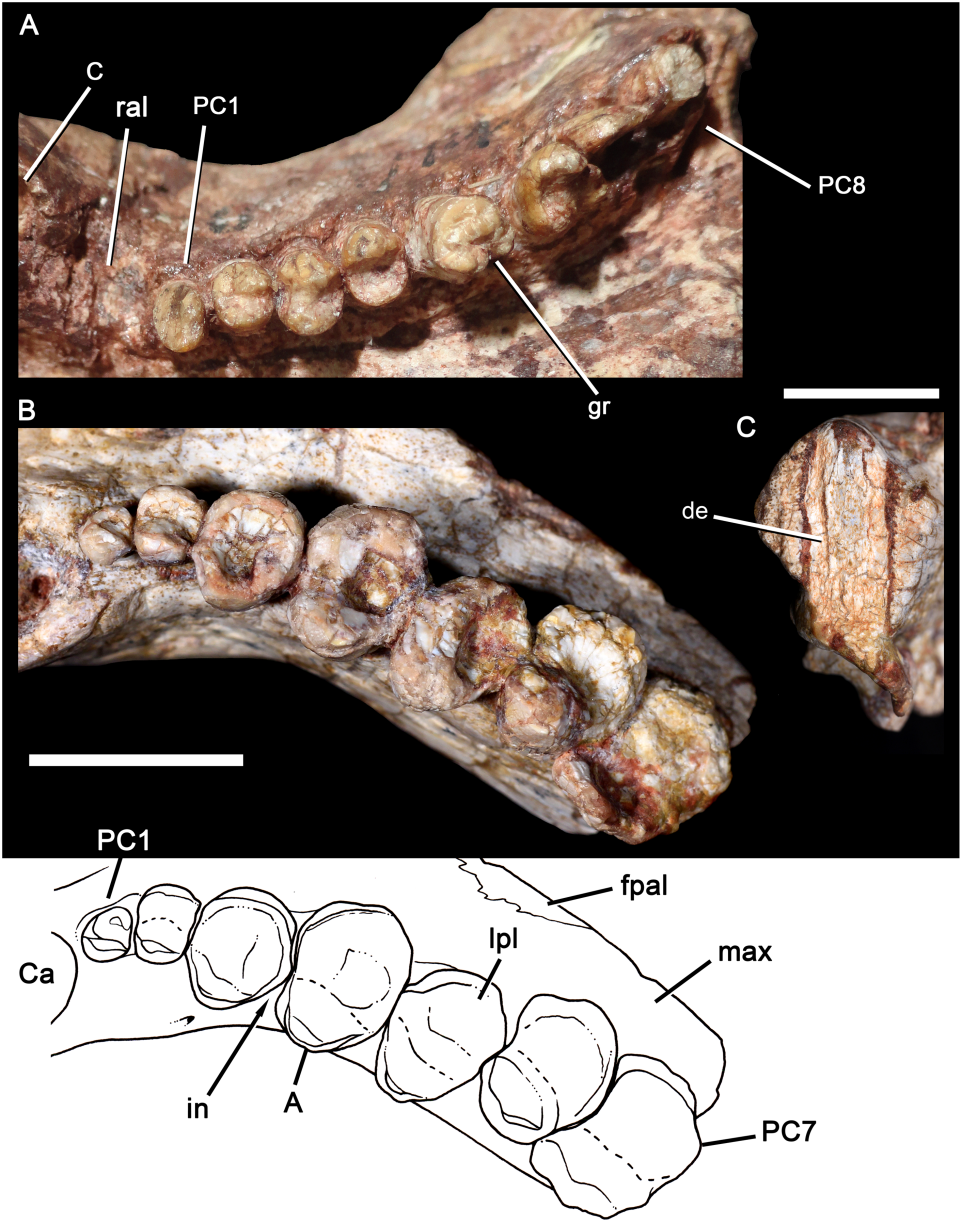
*Aleodon brachyrhamphus* and *A*. *cromptoni* sp. nov. Comparison of the left maxilla of *A*. *brachyrhamphus* (NHMUK PV R9390) from Tanzania (A), with the right maxilla of the specimen MPDC-501-117 from southern Brazil (B), in ventral view, with accompanying line drawing, and detail of the root through a natural section of the last preserved postcanine tooth in posterior view (C). Scale bar equals 10mm. Abbreviations: A, main cusp A; C, upper canine; Ca, upper canine alveolus; de, dentine; fpal, facet for palatine; gr, groove; in, interlocking; max, maxilla; PC, upper postcanine; ral, resorbed alveoli.

MPDC-501-117, right partial maxilla with broken canine and the first seven postcanines (Figs 3 and 4).

#### Diagnosis

Species of *Aleodon* distinguished (autapomorphies denoted by an asterisk) from *A*. *brachyrhamphus* by a distinct size increase between PC2 and PC3*; mesiodistally wider lingual platform of upper and lower postcanines*; less labiolingually elongate upper and lower postcanine crowns, without distinct constriction between labial cusp and lingual platform in the uppers; reduced transverse contact between lower postcanine crowns along tooth row*; and very reduced or absent canine-postcanine diastema in all ontogenetic states, resulting in an elevated number of postcanines compared with *A*. *brachyrhamphus* (up to 12).

#### Etymology

Named in honor of Alfred W. “Fuzz” Crompton, original describer of the genus *Aleodon*, for his many contributions to cynodont research.

#### Referred specimens

**Fig 5:**
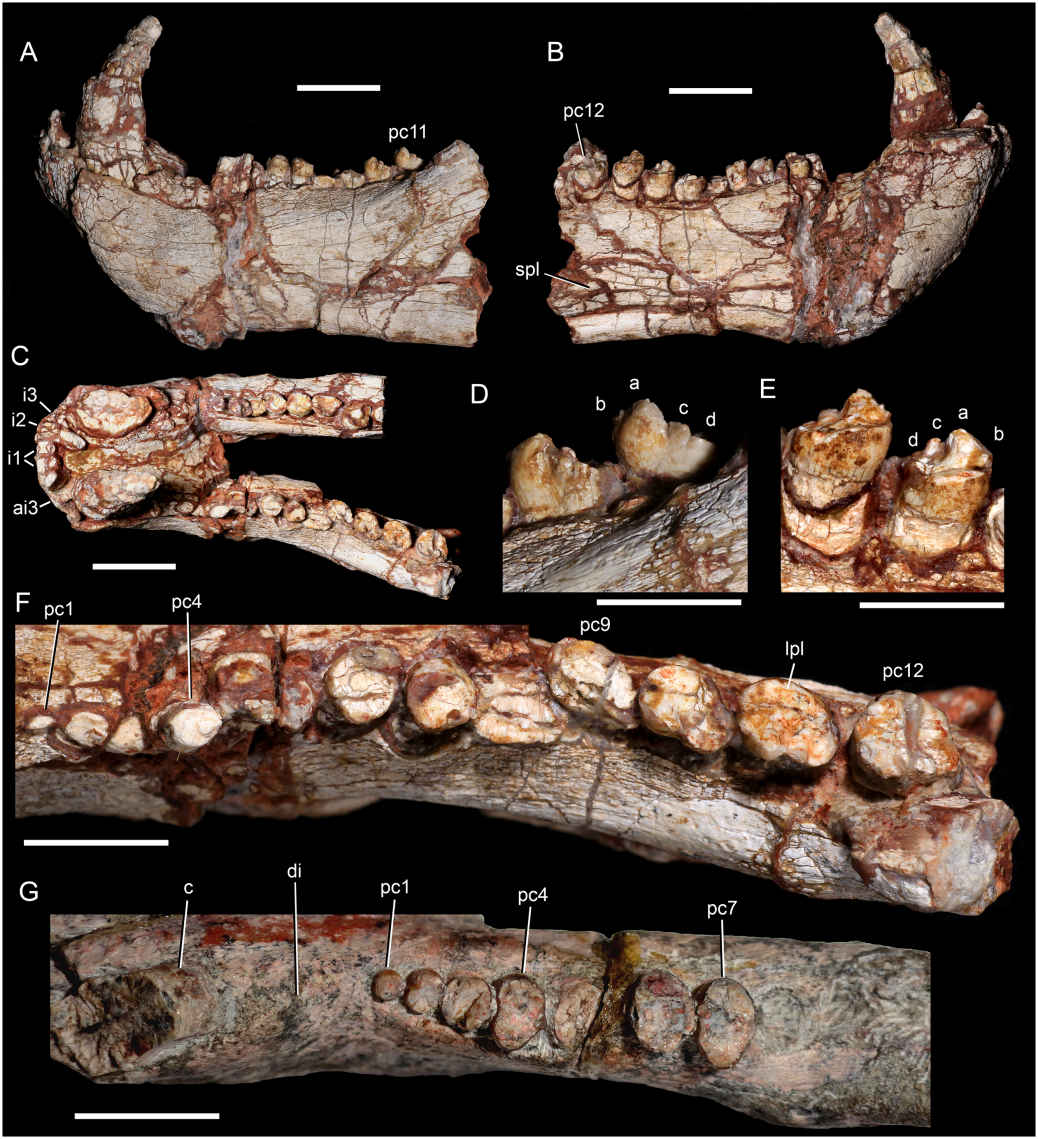
*Aleodon cromptoni* sp. nov. from southern Brazil. Specimen MCN-PV 10338, jaws in lateral (A), medial (B) and dorsal (C) views, with details of left pc10 and pc11 in labial (D) and lingual (E) views, and all the left postcanine tooth row in occlusal view (F). *Aleodon brachyrhamphus* from Tanzania. Holotype UMZC T906, detail of the left tooth row (canine and postcanines) in occlusal view (G). Scale bar equals 20mm in A-C and 10mm in D-G. Abbreviations: a-b-c-d, lower cusps on crown; ai3, alveolus incisor for i3; c, lower canine; di, diastema; i, lower incisor; lpl, lingual platform; pc, lower postcanine; spl, splenial.

**Fig 6:**
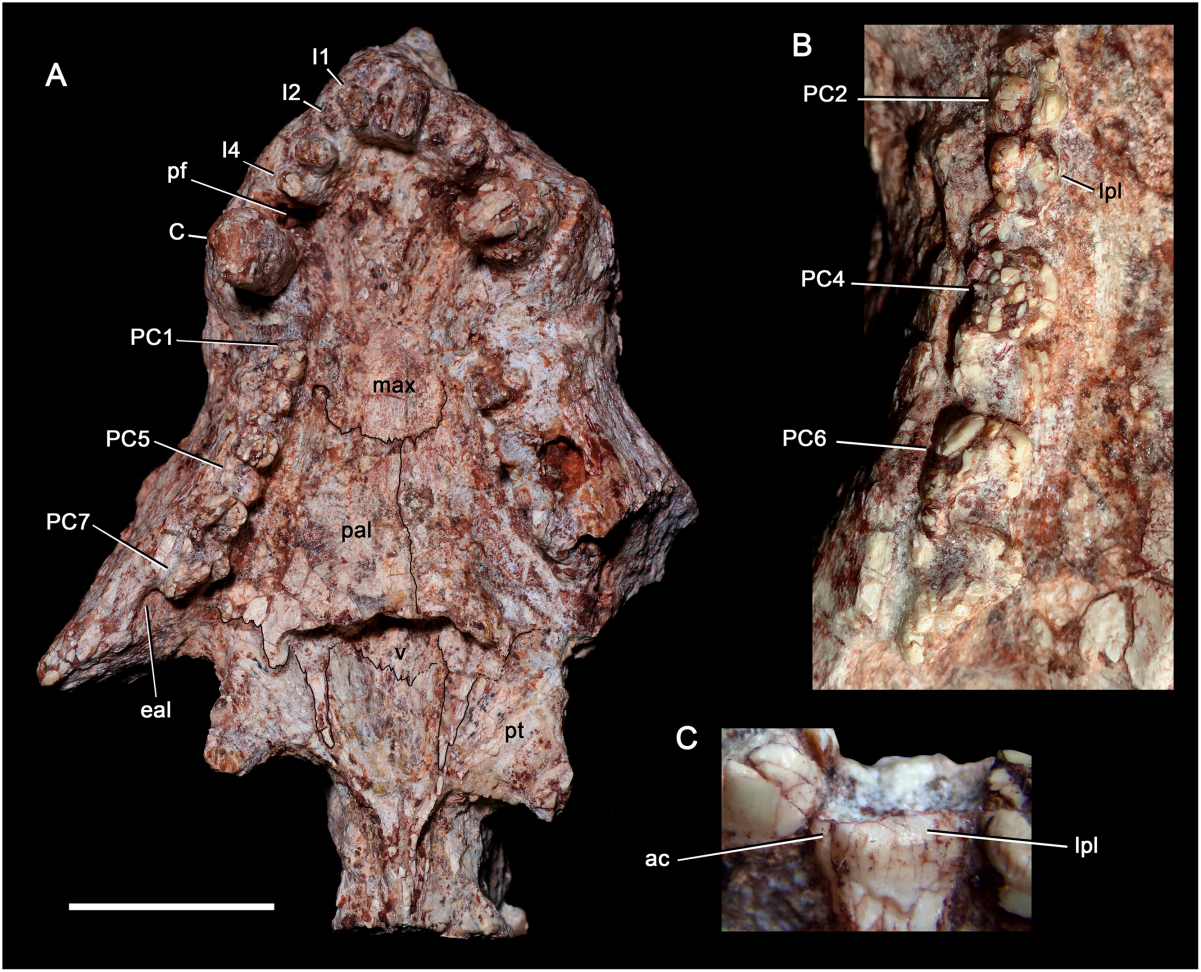
*Aleodon cromptoni* sp. nov. from southern Brazil. Specimen UFRGS-PV-0071-T, anterior half of skull in ventral view (A), detail of the right postcanine row (B), and detail of PC5 in lingual view (C). Scale bar equals 20mm in A. Abbreviations: C, upper canine; eal, empty alveolus; pf, paracanine fossa; I, upper incisor; lpl, lingual platform; max, maxilla; pal, palatine; PC, upper postcanine; pt, pterygoid; v, vomer.

**Fig 7:**
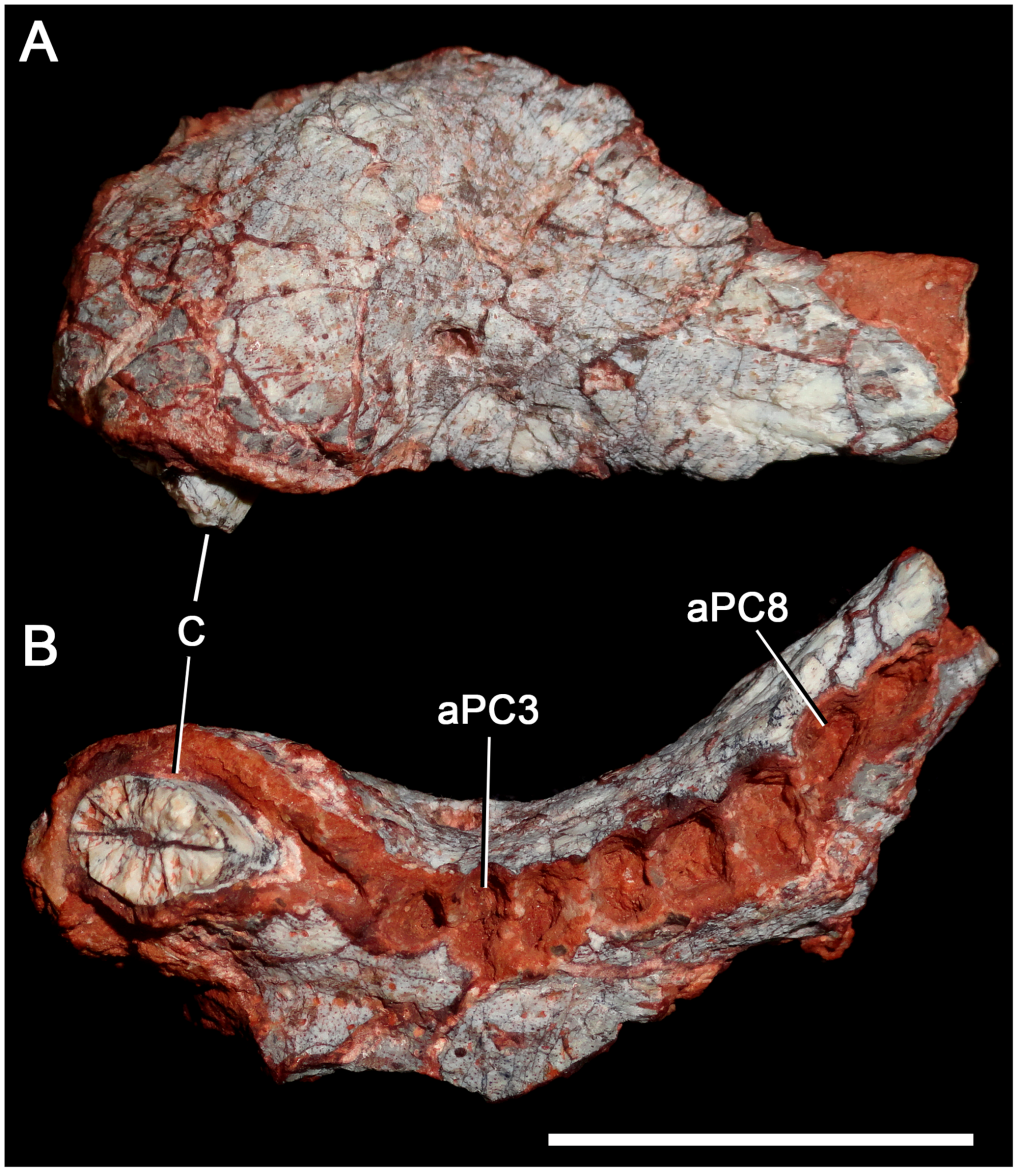
*Aleodon cromptoni* sp. nov. from southern Brazil. Specimen MMACR-PV-018-T, left maxilla in lateral (A) and ventral (B) views. Scale bar equals 20mm. Abbreviations: apc, alveolus for upper postcanine teeth; C, upper canine.

**Fig 8:**
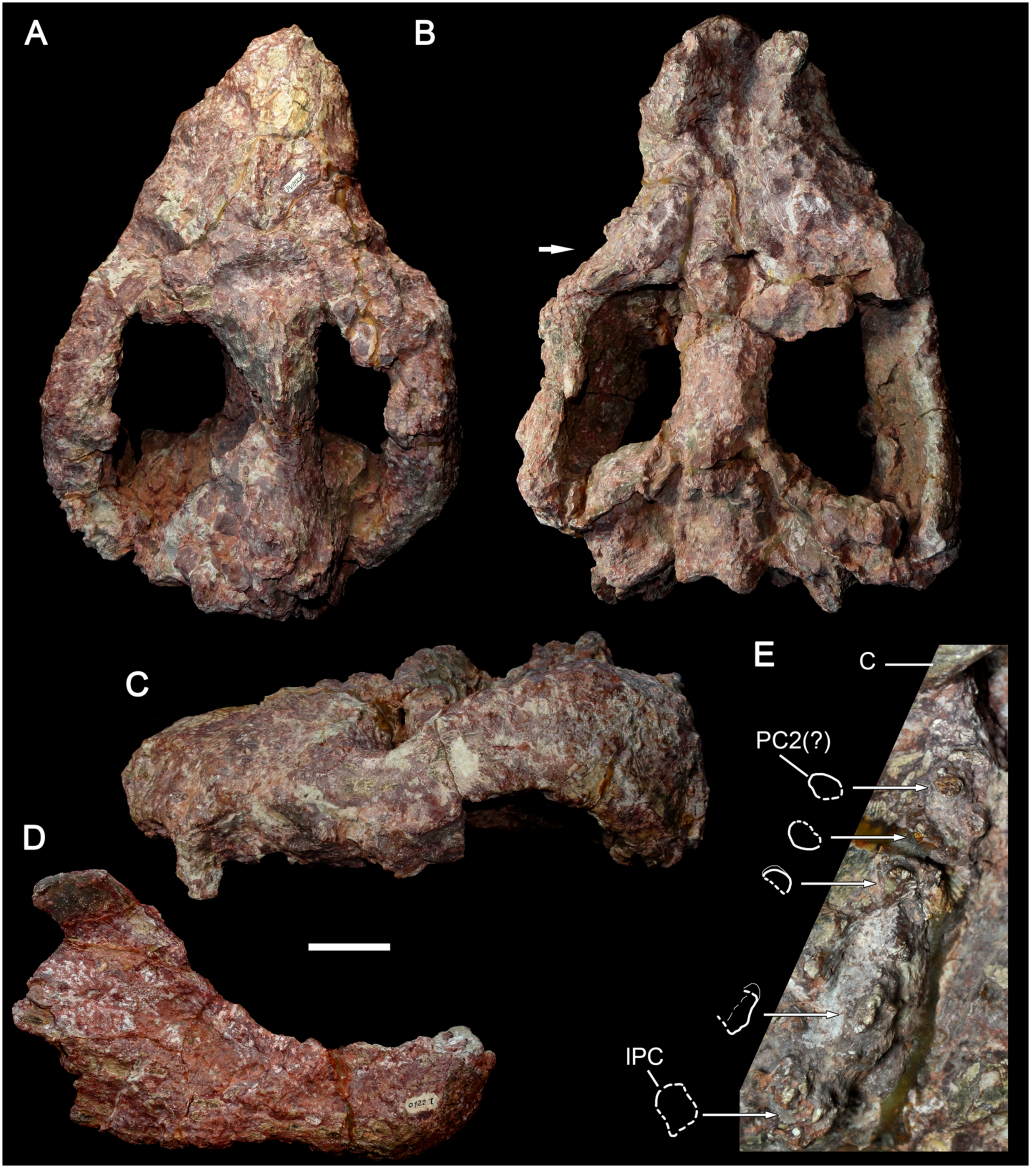
*Aleodon cromptoni* sp. nov. from southern Brazil. Specimen UFRGS-PV-0122-T, skull in dorsal (A), ventral (B) and lateral (C) views, left lower jaw (D) in lateral view, and detail of righ upper tooth row (E) in occlusal view. The arrow (B) indicates the level of the posterior edge of secondary palate. Scale bar equals 50mm. Abbreviations: C, upper canine; lPC, last upper postcanine tooth; PC, postcanine tooth.

**Fig 9:**
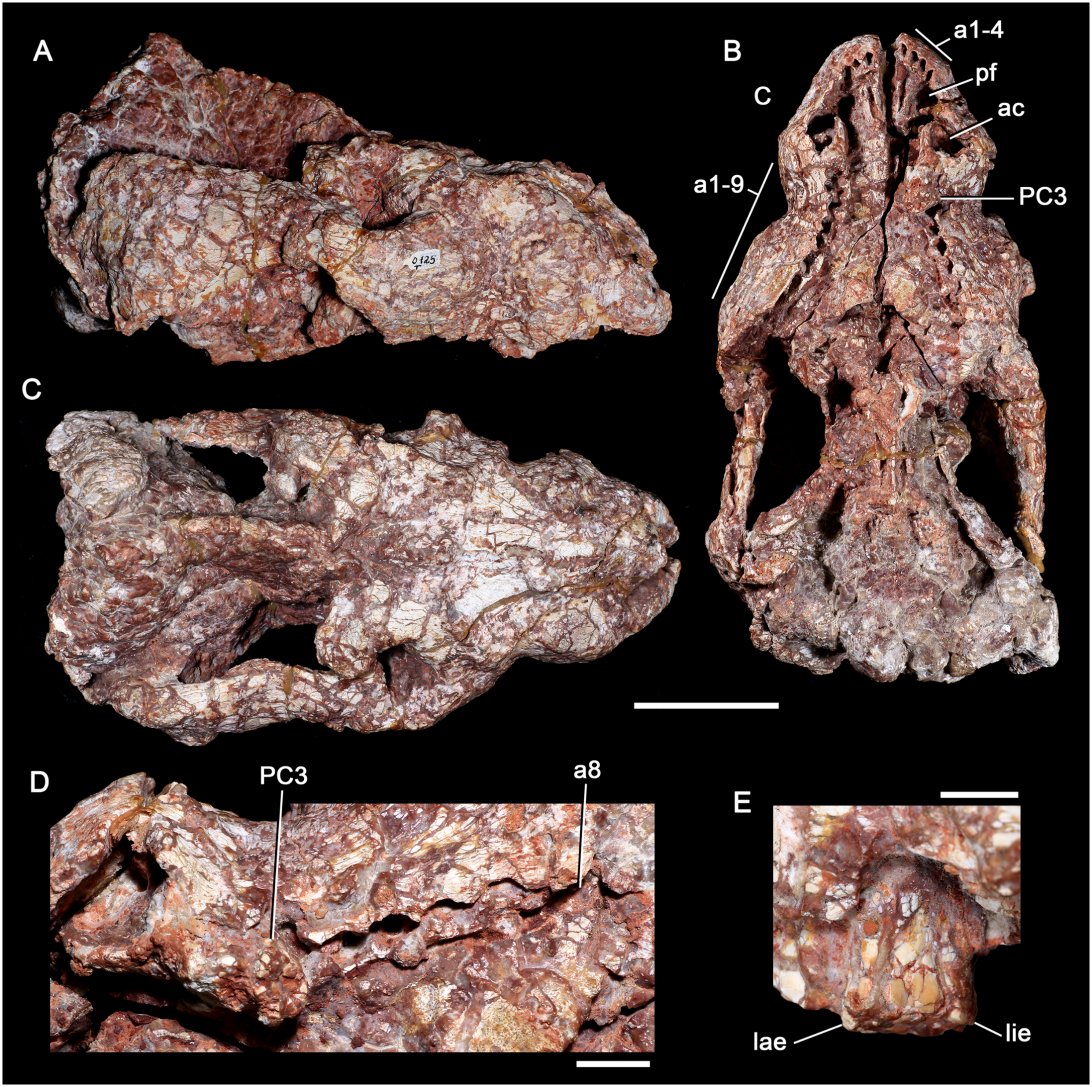
*Aleodon cromptoni* sp. nov. from southern Brazil. Specimen UFRGS-PV-0125-T, skull in lateral (A), ventral (B) and dorsal (C) views, with details of the left canine-postcanine tooth row in occlusal view (D) and of left postcanine 3 in distal view. Scale bar equals 50mm in A-C, 10mm in D, and 5mm in E. Abbreviations: a1-4, alveoli for incisors 1 to 4; a1-9, alveoli for postcanines 1 to 9; a8, alveolus for postcanine 8; ac, alveolus for canine; lae, labial edge; lie, lingual edge; pf, paracanine fossa; PC, upper postcanine.

**Fig 10:**
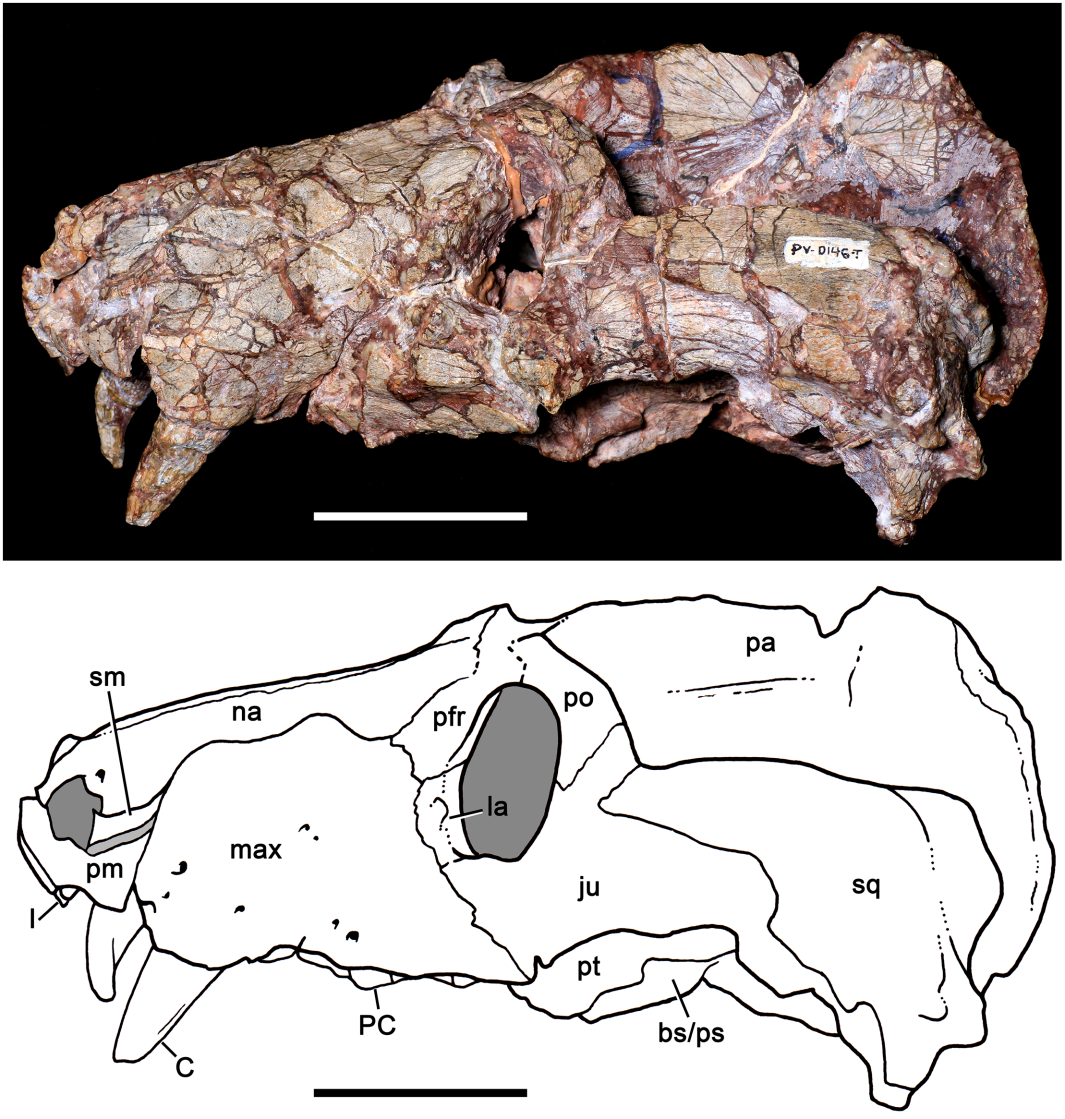
*Aleodon cromptoni* sp. nov. from southern Brazil. Specimen UFRGS-PV-0146-T, skull in lateral view with accompanying line drawing. Scale bar equals 50mm. Light gray indicates most affected areas with breakages. Abbreviations: bs/ps basisphenoid/parasphenoid; C, upper canine; I, upper incisor; ju, jugal; max, maxilla; na, nasal; pa, parietal; PC, upper postcanine; pfr, prefrontal; pm, premaxilla; po, postorbital; pt, pterygoid; sm, septomaxilla; sq, squamosal.

**Fig 11:**
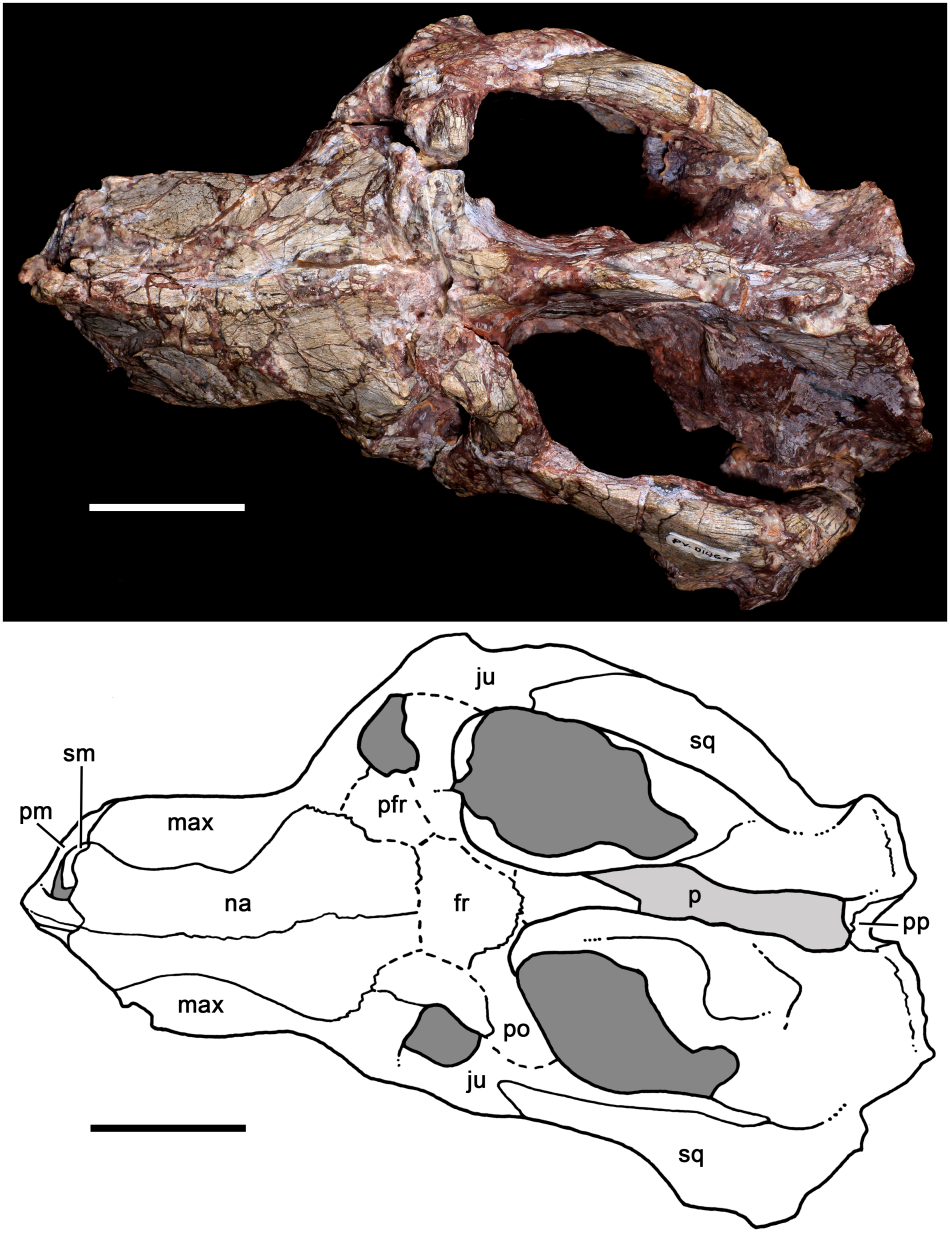
*Aleodon cromptoni* sp. nov. from southern Brazil. Specimen UFRGS-PV-0146-T, skull in dorsal view with accompanying line drawing. Scale bar equals 50mm. Light gray indicates most affected areas with breakages. Abbreviations: fr, frontal; ju, jugal; max, maxilla; na, nasal; pa, parietal; PC, upper postcanine; pfr, prefrontal; pm, premaxilla; po, postorbital; pp, postparietal; sm, septomaxilla sq, squamosal.

**Fig 12:**
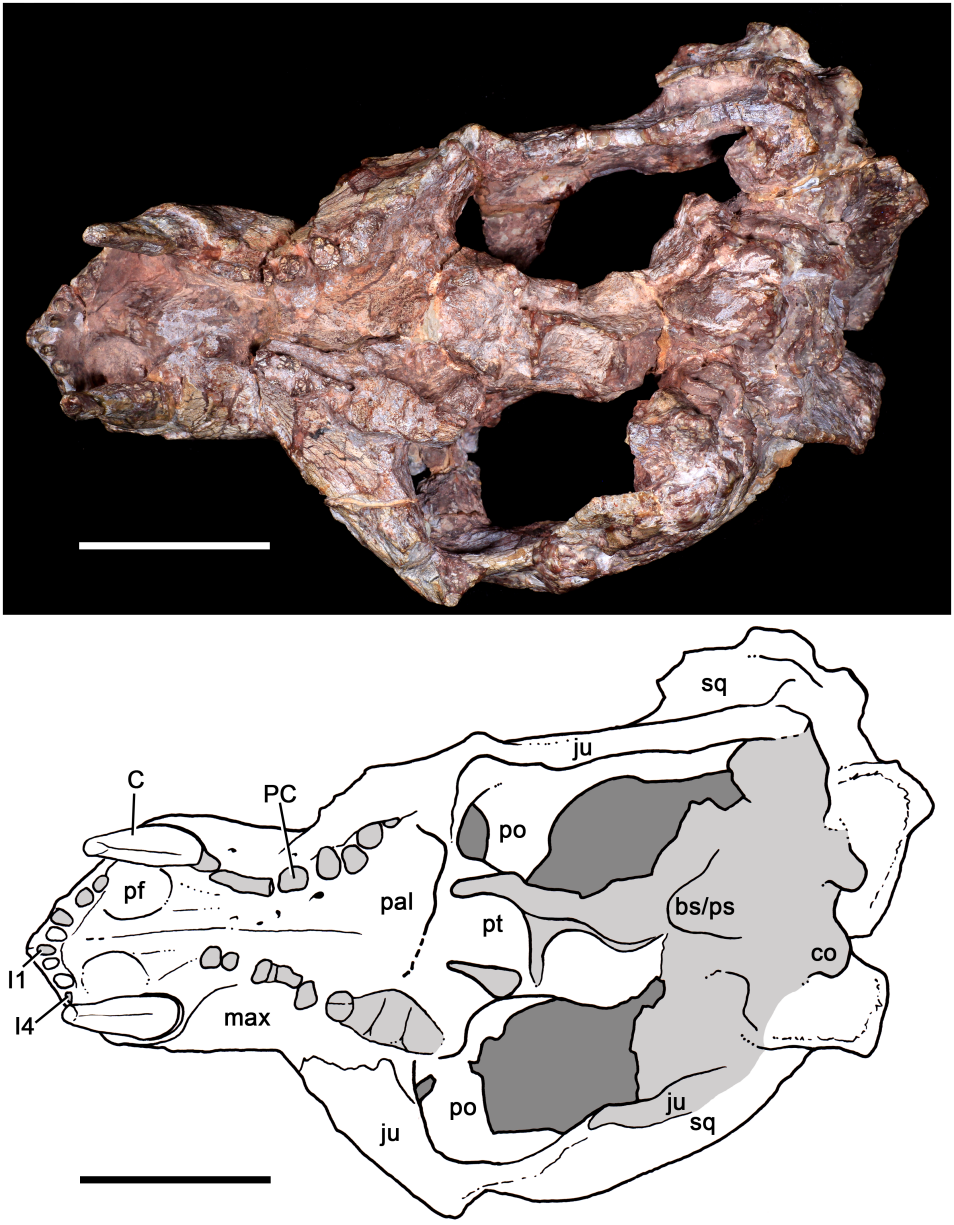
*Aleodon cromptoni* sp. nov. from southern Brazil. Specimen UFRGS-PV-0146-T, skull in ventral view with accompanying line drawing. Scale bar equals 50mm. Light gray indicates most affected areas with breakages. Abbreviations: bs/ps basisphenoid/parasphenoid; C, upper canine; co, condyle of exoccipital; I, upper incisor; ju, jugal; max, maxilla; pal, palatine; PC, upper postcanine; pf, paracanine fossa; po, postorbital; pt, pterygoid; sq, squamosal.

**Fig 13:**
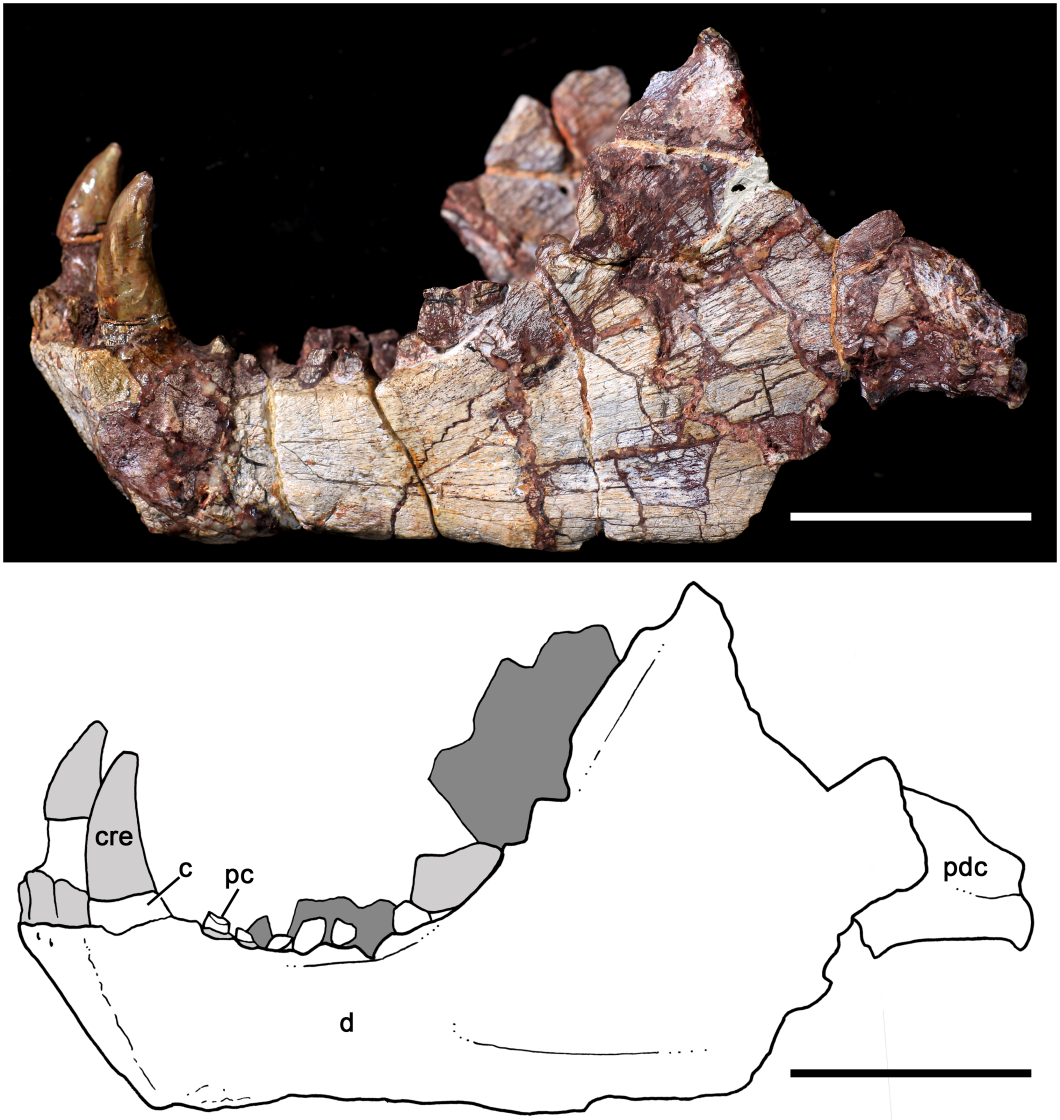
*Aleodon cromptoni* sp. nov. from southern Brazil. Specimen UFRGS-PV-0146-T, jaws in lateral view with accompanying line drawing. Scale bar equals 50mm. Abbreviations: c, lower canine; cre, canine reconstruction; d, dentary; pc, lower postcanine; pdc, postdentary bone complex. Dark gray indicates the right jaw and light gray indicates broken bone.

**Fig 14:**
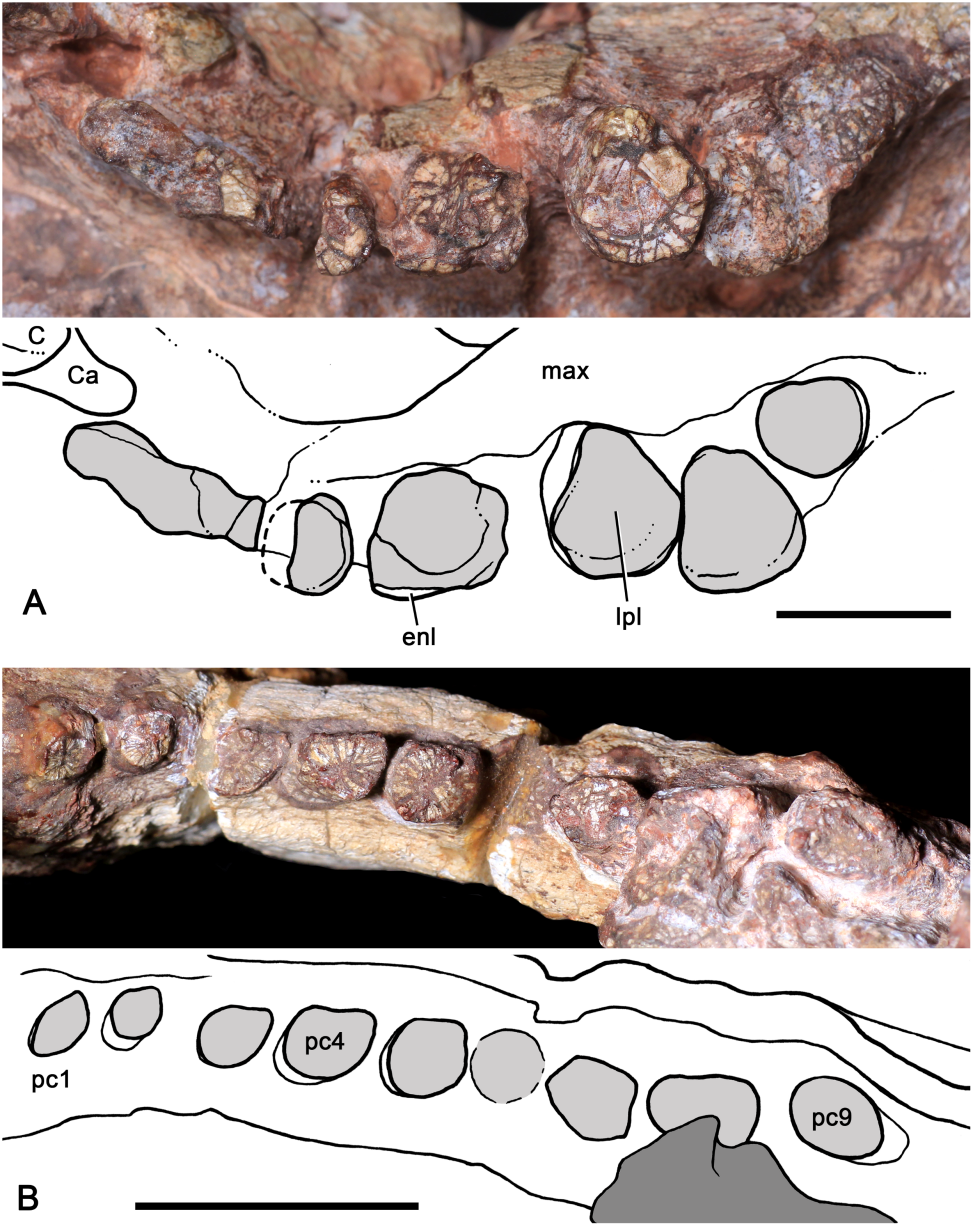
*Aleodon cromptoni* sp. nov. from southern Brazil. Specimen UFRGS-PV-0146-T, detail of left upper postcanine row (A) and left lower postcanine row, with accompanying line drawings. Scale bar equals 10mm. Abbreviations: C, upper canine; Ca, upper canine alveolus; enl, enamel layer; lpl, lingual platform; PC/pc, upper, lower postcanine.

**Fig 15:**
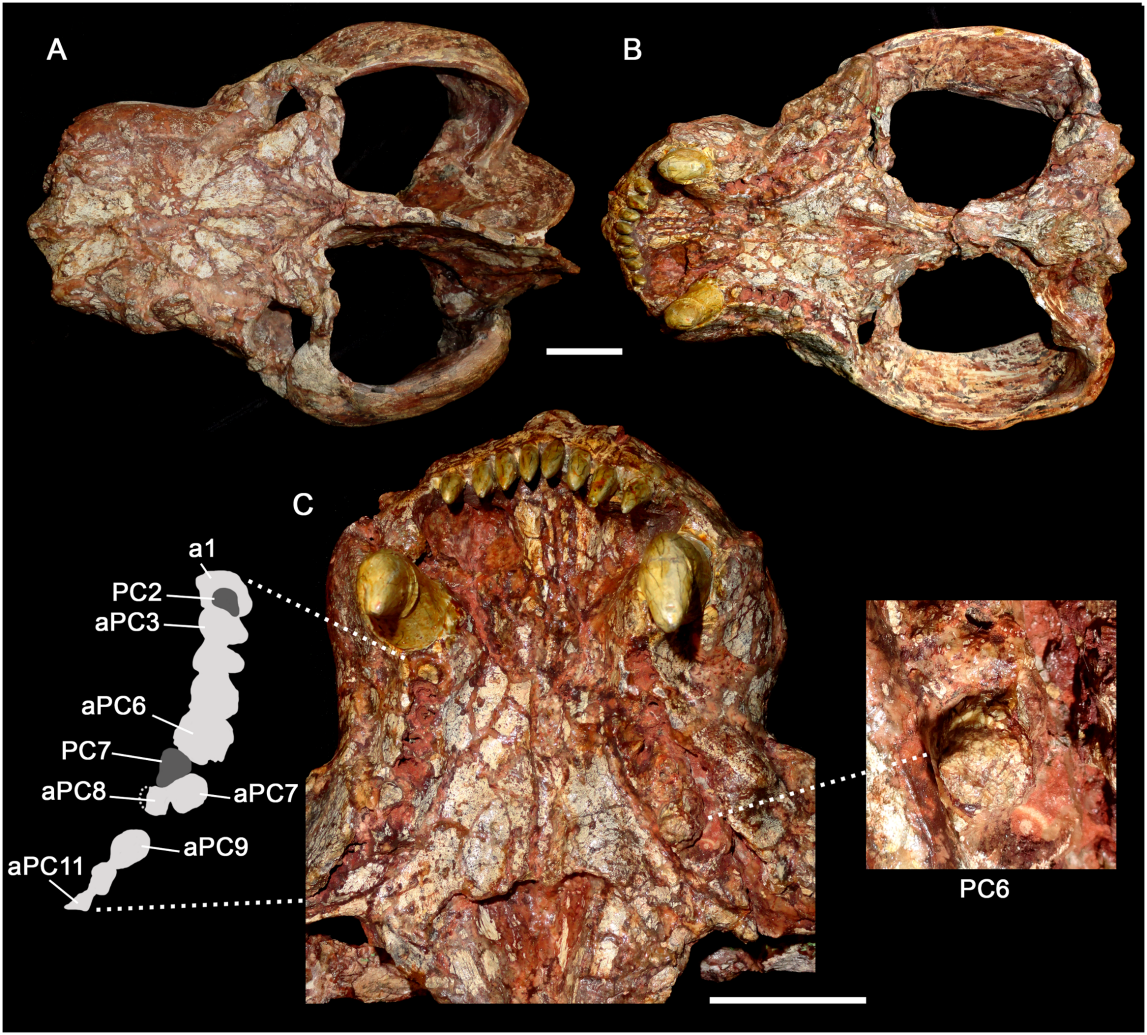
*Aleodon cromptoni* from southern Brazil. Specimen UFRGS-PV-0274-T in dorsal (A) and ventral (B) views, and detail of the snout in ventral view (C) with interpretative drawing of the right postcanine tooth row and detail of left PC6. Scale bar equals 50mm. Abbreviations: aPC, alveolus; PC, upper postcanine.

**Fig 16:**
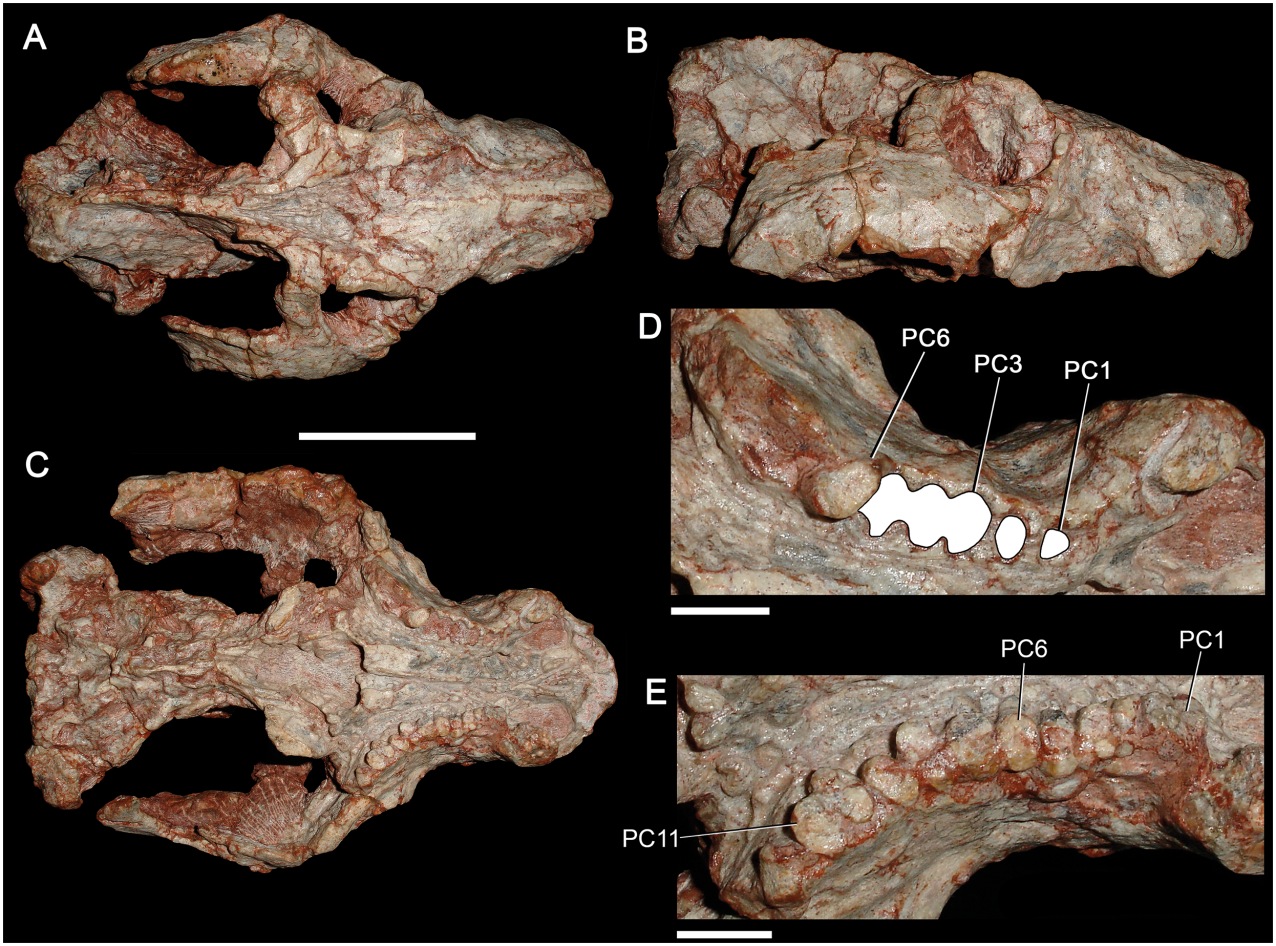
Specimen GSN EN-3 from the upper Omingonde Formation, Namibia, tentatively referred to *Aleodon cromptoni* sp. nov. Skull in dorsal (A), lateral (B), ventral (C) views, and detail of the right (D) and left (E) upper postcanine tooth rows in occlusal view. Alveoli of the right postcanine tooth row are highlighted in white. Scale bar equals 50mm in A-C and 10mm in D-E. Abbreviations: C, upper canine; PC, upper postcanine.

MCN-PV 10338, partial lower jaws with well-preserved dentition (Fig 5); UFRGS-PV-0071-T, anterior half of the skull with partial upper dentition (Fig 6); MMACR-PV-018-T, left maxilla with broken canine and nine alveoli for postcanine teeth (Fig 7); UFRGS-PV-0122-T, skull and partial jaws (Fig 8); UFRGS-PV-0125-T, distorted skull (Fig 9); UFRGS-PV-0146-T, skull (Figs 10–12), jaws (Figs 13 and 14), 26 presacral and four sacral vertebrae with some ribs, partial pelvis, left femur, two metapodials, and some indeterminate bones; UFRGS-PV-0274-T, skull (Fig 15). Specimens tentatively referred to this species include: GSN EN-3, complete skull with heavily worn teeth (Fig 16); MCP-PV-1695T, partially-prepared skull with incomplete dentition.

#### Locality and horizon

The holotype MPDC-501-117 was found in a railroad cut that crosses the Cria Farm (“Fazenda Cria”), about 300 meters north from the main house, in the municipality of Vale Verde (Fig 1). UFRGS-PV-0071-T and UFRGS-PV-0122-T were found in Sanga Pinheiro (also written as Sanga Pinheiros), UFRGS-PV-0125-T and MMACR-PV-018-T in Sanga Nicanor (also known as Sanga do Zé), and UFRGS-PV-0146-T in Sanga Pascual. Sanga Pinheiro, Sanga Nicanor, and Sanga Pascual outcrops are located about 10 to 20 kilometers south of the city of Candelária; the first two are in the Pinheiro region and the latter in the Bom Retiro region. MCN-PV 10338 was found in the Cortado site (also called Rincão da Porta), next to the highway RST-287 [41], municipality of Novo Cabrais (Fig 1). UFRGS-PV-0274-T lacks precise locality data. Based on its style of preservation it could possibly come from the classical Pinheiro region. The tentatively-referred specimen MCP-PV-1695T comes from the Porto Mariante 2 locality, municipality of Bom Retiro do Sul. All specimens come from the state of Rio Grande do Sul, Brazil.

All Brazilian specimens of *Aleodon cromptoni* are from the *Dinodontosaurus* AZ, Pinheiros-Chiniquá Sequence, Santa Maria Supersequence [35, 42] (Fig 2). Based on radiometric dating of the overlying unit (Santa Cruz Sequence; [43]) and tetrapod correlation with the Chañares Formation of Argentina (Ischigualasto-Unión Basin; [44–45]), the *Dinodontosaurus* AZ is considered to be Ladinian—early Carnian in age. The only potential non-Brazilian specimen of *A*. *cromptoni* is the isolated skull GSN EN-3 from the upper Omingonde Formation of Namibia [13]. The upper Omingonde Formation was previously correlated with the *Dinodontosaurus* AZ based on the shared presence of the dicynodont *Stahleckeria potens* [46].

#### Remarks on specimens

The holotype MPDC-501-117 was found in 1988 by the twin brothers Daniel (1930–2002) and Abraão (1930–2006) Cargnin, the renowned fossil-hunter priests of Rio Grande do Sul. For many years, MPDC-501-117 was listed as an indeterminate traversodontid cynodont in the exhibition of the Museu Padre Daniel Cargnin, located in the small town of Mata (municipality of Mata, Rio Grande do Sul). This material was unearthed at the railroad cut located a few kilometers from the train station Prof. Parreira, in the Vila Melos region. Specimens collected by the Cargnin brothers and also by the technician Valdor Costa (working at the UFRGS, under the direction of Prof. Mario Barberena) at this locality were previously catalogued as coming from the municipality of General Câmara. As was noted by Raugust [47], this area belonged to the 5° District of the municipality of General Câmara at the time, but in 1995 it became an independent district and was renamed municipality of Vale Verde (Fig 1). Drawings *in situ* of some specimens from this site (e.g., the rauisuchian UFRGS-PV-0152-T) are available in the field notes of Valdor Costa, deposited at the UFRGS.

UFRGS-PV-0122-T and UFRGS-PV-0274-T were previously listed as large specimens of *Chiniquodon theotonicus* by Abdala and Giannini [14], and UFRGS-PV-0146-T was described by Oliveira et al. [19] as *Chiniquodon* cf. *C*. *theotonicus*.

UFRGS-PV-0125-T is catalogued as having been found in Sanga Nicanor, Candelária, by Padre Daniel Cargnin in 1969. Historical data at the MMACR indicates that Sanga Nicanor refers to the old name for Sanga do Zé (located in the Pinheiro region), where specimen MMACR-PV-018-T was also found.

#### Fossil associations

In the same outcrop that yielded the holotype of *Aleodon cromptoni* were found: (1) the holotype skull of the traversodontid *Massetognathus ochagaviae* (UFRGS-PV-0255-T) [48], which is now lost; (2) an isolated osteoderm (UFRGS-PV-0285-T) and the partial skeleton of a rauisuchian (UFRGS-PV-0152-T), referred to *Prestosuchus chiniquensis* (see [49]); (3) at least two skulls of the dicynodont *Dinodontosaurus* sp. (UFRGS-PV-0128-T, UFRGS-PV-0228-T); and (4) additional fragmentary dicynodont and cynodont remains unidentifiable to genus.

At least five specimens of *Aleodon* come from Sanga Pascual, Sanga Pinheiro, and Sanga Nicanor (= Sanga do Zé). These outcrops are well-known for their fossil content (see [50–52]), including the dicynodonts *Dinodontosaurus pedroanum* and *Stahleckeria potens* [53–58], the cynodonts *Massetognathus ochagaviae* [59–60] (and at least one specimen identified as *M*. *pascuali* [61–63]), *Chiniquodon theotonicus*, and *Bonacynodon schultzi* [3, 14], indeterminate proterochampsians (*Chanaresuchus*/*Gualosuchus*), and the rauisuchian *Prestosuchus chiniquensis* [64–69]. The holotype of the procolophonian *Candelaria barbouri* comes from the Pinheiro region, without a more precise location [70].

The Cortado site (also called Rincão da Porta), which yielded MCN-PV 10338, has also yielded specimens of *Candelaria barbouri*, dicynodonts, and fragmentary cynodont material [41, 71]. Based on firsthand examination of this material by the lead author, we identify the synapsid specimens from this site as *Dinodontosaurus* sp. and *Massetognathus* sp. One specimen (UFSM 11079) from this locality, published as Cynodontia indet. [41], may belong to a new specimen of the probainognathian *Candelariodon barberenai*. The holotype of the archosauriform *Barberenasuchus brasiliensis* is also considered to be from this locality [52, 72].

### Description and comparisons

Descriptions of the known specimens of *Aleodon cromptoni* are presented below. The postcranial elements of specimen UFRGS-PV-0146-T are not described here, as they have been studied in detail by Oliveira et al. [19].

#### MPDC-501-117 (holotype)

This specimen consists of a partial right maxilla (Fig 3) bearing the base of the canine crown and the subsequent seven postcanines. The maxillary facial process is partially preserved, and is tall and dorsoventrally convex. There is also strong surficial convexity around the bulging canine root, followed by a conspicuous constriction delimiting a shallow concave surface above the postcanine row. The dorsal tips of the canine root and last three postcanine roots are exposed due to abrasion of the dorsal surface of the maxilla. A number of small vascular foramina are distributed across the lateral face of the maxilla. The majority is associated with the canine root, but a few larger foramina are also present above the postcanine tooth row. The alveolar edge is straight in lateral view, at the level of the canine; posteriorly, interdental processes are present as sharp projections that define successive ventrally-concave alveolar outlines for each postcanine. There is no maxillary platform, as in most non-gomphodont cynodonts (e.g., *Procynosuchus*, *Thrinaxodon*, *Lumkuia*, *Chiniquodon*, *Probainognathu*s, *Bonacynodon*, *Irajatherium*; [3, 32, 73–80]). In ventral view, the palatal process of the maxilla is partially preserved. It bears two large foramina, the larger one positioned medial to the level of PC1-PC2 and the smaller one medial to the level of PC3-PC4. Just medial to the canine root, the maxillary wall is broken, but there is clearly a space (the paracanine fossa) for housing the lower canine. This fossa is deep and is positioned anteromedial to the upper canine. The lateral suture for the palatal process of the palatine starts at the level of the PC4-PC5 contact and is strongly interdigitated. Medial and parallel to the postcanine tooth row, there is no longitudinal groove on the ventral surface of the maxilla as in *A*. *brachyrhamphus* and in contrast to the condition in most probainognathians (e.g., *Chiniquodon*, *Prozostrodon*; [32, 78, 81–82]) where this groove houses the tips of sectorial lower postcanines. In medial view, the maxillary surface is strongly concave, delimiting the wall of the nasal cavity.

The canine of MPDC-501-117 lacks most of its crown, but based on the cross-section of the preserved root it was a relatively large tooth, similar to other specimens of *A*. *cromptoni*. Its cross-section at base is ovoid, with the main axis slightly rotated in an anteromedial-to-posterolateral direction. The alveolus of the canine has a rounded pit at its posterior edge, which could represent a replacement pit. However, an erupting canine is not evident. The medial edge of the canine is offset lateral to the beginning of the postcanine tooth row.

The postcanine tooth row of MPDC-501-117 starts just posterior to the canine alveolus, without a diastema or evidence of resorbed teeth (Fig 3). There are seven postcanines, but this count is unlikely to reflect the original number of upper postcanines, based on breakage after PC7 and the condition seen in other specimens of *Aleodon* (see below). The postcanine crowns are in contact with each other and they diverge posterolaterally, forming a laterally concave tooth row. The general crown morphology consists of a bulbous main labial cusp that forms a sectorial crest and a lingual occlusal platform. They increase in absolute size posteriorly. However, the most evident variation in the teeth is that posteriorly, they exhibit greater expansion in transverse width than mesiodistal length. Consequently, in occlusal view, the postcanines go from being roughly circular anteriorly to transversely ovoid posteriorly. There is a kind of mesio-distal rudimentary interlocking among the postcanine teeth, where the convex distal edge of a preceding tooth contacts the lingually-offset convex mesial edge of the subsequent one (for a well-developed example of this, see the contact between PC5 and PC6 in Fig 3B). The tooth row shows no clear evidence of tooth replacement; most of the crowns are at the same height (with the exception of PC7), with a comparable degree of wear. There is a clear distinction between crown and root—a layer of enamel is visible labially and lingually on the former and it is also distinctly broader than the root. PC1 is the smallest of the series. It has a bulbous main cusp on its mesiolabial corner that bears a distal crest. The labial and mesial edges of the main cusp form a strong convex surface, whereas the distolingual edge is flat-to-concave and slopes gradually towards the lingual platform. The presence of a distal accessory cusp on the distal crest of the main cusp is not evident. A weak crest arises at the mesiolingual corner of the main cusp and descends to border the lingual platform. It bounds an oval concave surface restricted to the distolingual portion of the tooth. PC2 is similar in morphology to PC1 but the main cusp has a more labial position, with a convex labial edge and a slightly convex lingual edge that forms an abrupt angle with the lingual platform. The main cusp is mesiodistally asymmetrical with the distal crest more elevated than the mesial one, possibly indicating the presence of a worn-off accessory distal cusp. In this postcanine, the lingual platform extends across the mesiodistal length of the crown. It is slightly transversely broader in its distal portion. The lingual platform is bounded by a rim of enamel and the occlusal surface exposes the underlying dentine. Between PC3 and PC2 there is a considerable increase in size, whereas in the rest of the row this change is more gradual. PC3 has a main cusp with mesial and distal crests that in occlusal view form an almost 90° angle. The lingual platform is circular in outine in occlusal view, with a shallow fossa on its distolabial side. The PC4 and PC5 are similar in morphology to PC3 but the distolabial fossa on the lingual platform is deeper. PC5 seems to be slightly smaller than PC4, but this is an artifact, because most of the lingual enamel layer of PC5 is broken. PC6 is not well preserved. The crown has a more ovoid shape than the preceding teeth. The main cusp is bulbous and situated on the mesiolabial side where the lingual platform develops from the mesial to the distal positions of the crown. The last postcanine, PC7, is also badly preserved. The crown seems to be slightly nearer to the alveolar level, with no exposure of the root in labial view. Consequently, it could represent a more recently erupted postcanine, at the back of the series. In PC7, the lingual platform is more developed in comparison to the other postcanines.

In general, the postcanine morphotype of MPDC-501-117 is most similar to that of *Aleodon brachyrhamphus* among cynodonts ([20]; NHMUK PV R9390, UMZC T906) (Fig 4), differing considerably from that of other known taxa [3, 14, 32]. Although gomphodont cynodonts also possess transversely wide postcanines, the crown morphology in these taxa is fundamentally different: the upper postcanines have a conspicuous transverse crest, often with three discrete cusps, that divides or bounds the occlusal basin and opposes a transverse crest with two main cusps generally present on the lower postcanines (e.g., [80]).

#### MCN-PV 10338

This specimen consists of a partial mandible with the best preserved lower dentition for *A*. *cromptoni* (Fig 5). Both mandibular rami are broken, the left at the base of the coronoid process and the right somewhat anterior to that, at the level of pc9. The dentaries are fused at the symphysis. The symphysis is long and extends posteriorly to the fourth postcanine. It has a flat anterior surface that projects anteroventrally. The anteroventral edge of the symphysis and the ventral edge of the dentary form a conspicuous mentum. The horizontal ramus of the dentary is anteroposteriorly short and dorsoventrally tall, with a robust aspect. The alveolar border is almost straight whereas the ventral edge is gently concave. The base of the coronoid process is preserved on the left side. It is stout and its preserved portion has an angle of ~140° relative to the alveolar edge. Based on the preserved portion of left dentary, the masseteric fossa is relatively shallow and extends anteriorly to the level of the antepenultimate tooth. In medial view, the splenial is a flat and tall bone covering the Meckelian groove (Fig 5B). The groove is positioned below the mid-height of the dentary and runs parallel to the ventral edge of the dentary.

There are five partial incisors (left i1-i2 and right i1-i3) and the alveolus for an extra one (left i3) preserved in the lower jaw. The whole crown is not preserved for any of these incisors. The incisors are large with a mesiodistally flattened cross-section. The i1 and i2 are similar in size and slightly larger than i3, based on the preserved cross-sections. The incisors are tightly packed, and lack a diastema with the canine. As in the previous specimen, the canine is large, with an oval cross-section. The canine tip is acute and prominently recurved. The left and right canines are close to one another across the midline (separated by only 9mm), although this may be due in part to postmortem deformation. There is not a clear diastema with the postcanine tooth row, although this portion of the jaw is poorly preserved. The left mandibular ramus preserves 12 postcanines whereas the right ramus partially preserves nine teeth. The postcanines increase in size posteriorly and go from being circular (in the anterior half) to oval (in the posterior half) in occlusal view, with a short labial sectorial crest and a lingual platform with basin. Due to wear or breakage, a cuspidate sectorial labial edge is not observed in pc1 to pc9.

The left pc1 to pc3 only preserve the root. The left pc1 has no crown and the small portion of root seems to have been in the process of resorption, which also seems to be the case for the right pc1 and pc2. The left pc4 is circular in cross-section and exhibits an unusually tall root, which probably slipped out of the alveolus post-mortem. The shape of the left pc5 is distorted by a large, transverse crack running across it. The left pc6 and pc7 are the last circular-shaped teeth of the row. In these teeth, the crown is low, with elevated labial and lingual edges bordering a concave occlusal basin. This basin is not covered by enamel, although a thick layer of enamel covers the crown externally. The pc8 and pc9 are ovoid, with completely worn-off crowns. The enamel layer that covers the crown on the labial surface is very thin. The pc10 to pc12 are the best preserved elements, with nearly unworn crowns. They are oval in cross-section, with a low crown. The crown is conspicuously wider than the root, which is circular in cross-section. The labial crest is low with a main cusp a that is curved posteriorly and slightly laterally. There are three accessory cusps: a tiny mesial cusp b and two distal cusps, c and d (Fig 5D and 5E). The main cusp a is separated from cusp c by a deep notch. The lingual surface of the sectorial crest slopes towards the lingual platform gradually, without a distinctive step. This lingual surface and the occlusal platform have a thin layer of enamel, whereas the enamel that covers the crown externally is relatively thick. Accessory cusps on the lingual edge of the lingual platform are not evident. In lateral view, the last three postcanines have a similar cusp pattern to that of *Chiniquodon* (see Discussion), although the crown is lower and the cusps are more discrete. The right postcanines do not provide further details and their crowns are largely worn off.

#### UFRGS-PV-0071-T

This specimen consists of the anterior half of a skull without lower jaws (Fig 6). This specimen is poorly-preserved overall, having suffered from the calcitic replacement and secondary expansion typical of Santa Maria fossils. It is referred to *A*. *cromptoni* because of the presence of transversely-expanded postcanines, with a labial cusp and broad lingual platform (Fig 6B). The snout as preserved is similar to that of other *Aleodon* specimens. UFRGS-PV-0071-T is much smaller than the holotype and represents one of the smaller known specimens of *A*. *cromptoni* (58.6mm palatal length and 56.8mm muzzle length). Four upper incisors are preserved on each side of the snout. They are sub-conical, evenly spaced, posteroventrally angled, and decrease in size posteriorly. The right I2 and left I1 and I3 were in process of eruption at the time of the animal’s death, with I2 and I3 less erupted than I1. The canine is large and ovoid in cross section. There is a deep paracanine fossa positioned anteromedial to the canine. All of the postcanine crowns in this specimen are damaged, with numerous cracks across their surface, but the postcanine tooth row is somewhat better preserved on the right side of the skull. The right tooth row consists of seven postcanines plus an alveolus at the rear with an erupting tooth. The postcanines in this specimen are circular to oval in cross section, and their overall morphology indicates the presence of a lingual platform, which is clearly evident in the right PC5-6. The right PC5 exhibits an elevated labial crest and a very small accessory cuspule on the distolingual corner of the lingual platform. This cuspule is worn off apically, but can be distinguished from the rest of the crown by the presence of a short vertical notch. Cuspules of this kind would be quickly eroded during mastication and, therefore, are worn out in functional teeth. The postcanine tooth row is concave laterally and projects lateral to the subtemporal fenestra. The last postcanine is positioned posterior to the level of the anterior orbital edge and just posterior to the anterior edge of the subtemporal fenestra. The secondary palate is long, reaching the level of the last postcanines (Fig 6A). It is made up almost equally by the maxilla and palatine. The anterior half of the secondary palate is almost flat, outside of the conspicuous paracanine fossae, whereas the posterior half is transversely wider and slightly convex. Just medial to the last postcanine teeth there is a shallow concavity on the palate. The medial process of the palatine, which contacts the primary palate, projects posteromedially onto the pterygoid body by means of a triangular process. The pterygoid wings are wide and, based on the preserved portion, are shorter and less sharply pointed at tip than in *Chiniquodon* [14]. They are posteriorly and slightly ventrally projected. Along the midline, the pterygoids unite to form a sharp medial crest. The dorsal and lateral aspects of the skull are in need of futher preparation.

#### MMACR-PV-018-T

This specimen consists of a left maxilla with the root of the canine and nine postcanine alveoli (Fig 7). The size of this specimen is similar to UFRGS-PV-0071-T; together these represent the smallest known individuals of *A*. *cromptoni*. The maxilla is tall with a convex surface on the bulging canine root and a concave constriction posteriorly. There is a large maxillary foramen, positioned above the level of the alveolus for PC2. The canine root is oval in cross section and, as in other specimens of *A*. *cromptoni*, the postcanine row diverges posterolaterally. The first postcanine alveolus is oval. Posteriorly, the alveoli are transversely elongated until the alveolus of PC6, which is the transversely widest of the row. After this point, the PC8 and PC9 alveoli become transversely narrow and oval, with the main axis mesiodistally oriented (Fig 7). The presence of transversely narrow alveoli (or teeth) at the rear of the upper tooth row is also observed in specimens UFRGS-PV-0071-T (see above) and UFRGS-PV-0125-T (see below). This indicates the presence of a few sectorial postcanines in the last position of the tooth row, another feature convergent on the condition in gomphodonts like *Diademodon*. The alveolus of PC10 preserves only its mesial edge. It apparently housed a sectorial tooth, as did two preceding alveoli.

#### UFRGS-PV-0122-T

Although very poorly preserved, this specimen is important in representing the largest known individual of *Aleodon* (310mm basal skull length). It consists of the skull (Fig 8) and a complete right and partial left lower jaw. The preorbital region is shorter than the postorbital, and this does not appear to be due to deformation. The temporal region is wide, and bounded by a deep, prominently arched zygoma (Fig 8). The zygomatic arch is similar to that of UFRGS-PV-146T. Its ventral edge is strongly concave ventrally and at its anteroventral corner there is a marked angulation, as in *Chiniquodon* [14]. The parietal crest is tall and the skull roof tapers anteriorly. The secondary palate is also well-developed posteriorly, differing from gomphodont cynodonts. The precise number of incisors and postcanines is unknown. The upper canines are extremely large, with the preserved portion of the right crown being 46 mm long. The shape of most postcanines is indiscernible but a few ones from the right side clearly exhibit a circular cross-section in occlusal view, indicating a lingual platform. The first postcanine is positioned medially to the level of the canine, similar to other specimens (small and large) of *Aleodon*, and differing from *Chiniquodon*, in which the canine and first postcanines are aligned [73, 81–82]. The jaw is robust with an elongated symphysis, high horizontal ramus, and tall coronoid process (Fig 8). The angular process of the dentary is posteriorly broken and part of the post-dentary complex is preserved. The lower postcanines are not available.

#### UFRGS-PV-0125-T

This specimen consists of a laterally compressed, poorly preserved cranium (Fig 9) suffering from calcitic expansion. The majority of the dentition has fallen out, with the exception of the first three left postcanines. There are four alveoli for incisors, which decrease slightly in size posteriorly. The canine alveoli are large, as in other specimens of *A*. *cromptoni*. The left postcanine tooth row preserves the PC1 to PC3 plus five alveoli, and the right side preserves at least nine alveoli. The postcanine tooth row is laterally concave, with its posterior end angled posterolaterally even though the skull is compressed (unlike the condition described for UFRGS-PV-0146-T below). The PC1 and PC2 are smaller than PC3, peg-like in shape and without a diastema with the canine. Their crowns are poorly preserved. The PC3 is an intact but heavily worn crown. PC3 bears a low labial cusp and lingual platform and is transversely expanded and ovoid, similar to that of other *Aleodon* specimens. The last two alveoli in the right tooth row are markedly narrower in their transverse dimensions than the preceding ones, suggesting that these teeth may have been sectorial (Fig 9). In addition to postcanine morphology, identification of this specimen as *Aleodon* rather than as a gomphodont is supported by the great length of the secondary palate (terminating posterior to the postcanine tooth row), lack of maxillary platform, and the proportionally small size of the incisor alveoli. The zygomatic arch of this specimen (best preserved on the right side) and is extremely tall and robust, as in other large specimens of *Aleodon*. The dorsal profile of the skull is also similar to other *Aleodon* specimens, with a tall parietal crest at its posterodorsal edge.

#### UFRGS-PV-0146-T

Oliveira et al. [19] previously described this specimen in detail, albeit focusing on the postcranial elements. It was referred to *Chiniquodon* because of the presence of a relatively short snout, very deep zygomatic arch (Fig 10), angulation between the posterolateral portion of the maxilla and anteroventral margin of the zygomatic arch (Fig 10), and long secondary palate [19], following the taxonomic proposal of Abdala and Giannini [14]. With regard to the postcanines, they mentioned that because the postcanine crowns are broken, their presumable sectorial morphology could not be observed. The few differences observed between the postcranium of this specimen and that previously known for *Chiniquodon* [83] were considered to be attributable to intraspecific variation [19].

Although the postcanines of UFRGS-PV-0146-T are poorly preserved, they are not all broken (contra [19]) (see Figs 12 and 14). Several of the crowns are intact, albeit heavily worn, and exhibit the characteristic postcanine morphology of *Aleodon* (especially the left PC5; see Fig 14 and description below). The morphology of the dentition and its biostratigraphic (i.e., *Dinodontosaurus* AZ) and geographic provenance allow us to refer UFRGS-PV-0146-T to *A*. *cromptoni*. This specimen is larger than the holotype, but smaller than UFRGS-PV-0122-T (see S1 File).

The skull is slightly laterally compressed and bears several cracks that obscure the recognition of cranial sutures. The snout is shorter than the temporal region and the orbits face anterolaterally. Although deformed, the temporal fossae are clearly sub-rectangular, widening posteriorly. The zygomatic arch, postorbital bar, parietal and lambdoidal crests are robust. In lateral view, the profile of the skull is roughly triangular, with an elevated parietal crest that gradually slopes downwards anteriorly. The maxillary facial process is tall, with a prominent convexity produced by the canine root. The premaxilla is small and has a conspicuous ascending internarial process. The septomaxilla has a large lateral process extending posterodorsally between the nasal and maxilla. The zygomatic arch is composed of the squamosal and jugal. The jugal projects far posteriorly to reach the ventrolateralmost portion of the squamosal, longer than in most *Chiniquodon* specimens [14]. The squamosal projects anteriorly over the jugal, terminating below the posterior portion of the postorbital bar. The ventral edge of the zygomatic arch is highly concave, producing a prominent angulation at its anterior edge (below the postorbital bar) as in *Chiniquodon*. The deepest part of the zygomatic arch is in its posterior portion, which is as tall as the snout height at the middle of the tooth row. In ventral view, the secondary palate is long, extending to the level of the last postcanine. Based on a partial suture preserved on the left side, the lengths of the maxilla and palatine in the secondary palate are similar, as in other specimens of *A*. *cromptoni* (see above). The pterygoids are not well preserved and the primary palate and basicranial region are badly distorted. In posterior view, the occipital plate is triangular, with a deep, v-shaped notch separating the lambdoidal crest from the zygomatic arch.

The dental formula is 4I/3i, 1C/1c, 8PC/9pc. The upper incisors are subconical and slightly posteroventrally projected. The incisors are closely spaced. They increase in size posteriorly and there is a short diastema with the canine. The canine is a very large tooth (see S1 File) that is oval in cross section at the base. The upper and lower canines are recurved, although this is less evident in the somewhat-distorted left upper canine (Fig 10). There is no evidence of canine serrations. The postcanine tooth rows are not well preserved and slightly deformed, but it is evident that there is no diastema with the canine. The left tooth row is laterally concave and projects lateral to the subtemporal fossa, as in the holotype of *A*. *cromptoni*. On the other hand, the right tooth row seems to be straighter due to deformation of the maxillary body and zygomatic arch. The left tooth row preserves evidence of eight teeth, of which only four retain remnants of crowns. One in particular, the PC5, is largely intact and exhibits the diagnostic morphology of *Aleodon*, with a single labial cusp and large, transversely expanded lingual platform (Fig 14). The circular to ovoid cross-section of the crown, due to the development of the lingual platform, is also evident in PC3 to PC7 on the left side and the middle postcanines of the right side. On the right side, the precise number of teeth is uncertain due to poor preservation; parts of at least five crowns are preserved.

#### UFRGS-PV-0274-T

This specimen is a relatively large skull, with a total length of 277mm. The skull is dorsoventrally compressed and there are several cracks on its surface (Fig 15). Part of the zygomatic arch, all the incisors, and both canines are reconstructed with plaster and resin. It has the same features observed in the aforementioned specimens, including a short and wide snout, with a conspicuous bulging at the canine root. The secondary palate is long and slightly convex at its posterior portion. The postcanine tooth rows only preserve portions of the right PC2 and PC7 and left PC6. The right postcanine row includes a very mesiodistally short alveolus for PC1. The PC2 crown is broken with a circular cross section. The alveoli for PC3 to PC6 are oval with their main axis, transversely oriented. The PC7 preserves part of the crown. It is oval shaped, in occlusal view, with a lingual platform. It seems to be an erupting tooth but due to the poorly preserved portion of the maxilla this is uncertain. At this point, the maxilla is obliquely crushed and the posterior portion slightly shifted laterally on the anterior portior, so the exact orientation of these teeth is difficult to discern. For example, the alveolus for PC8 seems to be almost at the same tranverse level as the alveolus of PC7. The alveoli for PC7 and PC8 are transversely narrow and decrease in size posteriorly, indicating the presence of sectorial teeth at the back of the tooth row. On the left side, the postcanine row preserves two circular alveoli for PC1-PC2. The alveolus for PC3 is considerably larger transversely, indicating a conspicuous change in tooth size at this point. The following alveoli are also transversely wide until reaching the alveolus for PC7. The last alveoli are partially preserved and the total number in this side is unknown. This row preserves part of the crown of PC6. It is somewhat oval in cross-section and bears the lingual platform characteristic of *Aleodon*. As in the previously-described specimens and differing from large specimens of *A*. *brachyramphus*, there is no diastema between the canine and postcanine teeth.

## Discussion

### Assignment of the Brazilian specimens to *Aleodon*

MPDC-501-117 is referred to the genus *Aleodon* based on the presence of labiolingually-expanded postcanines with a tall main labial crest, dominated by a main cusp and a well-developed lingual platform in the preserved upper postcanine teeth. The lingual platform is not developed to the same degree in any other Triassic probainognathian, including chiniquodontids [14, 15, 78–79, 84], ecteniniids [85–87], probainognathids [3, 74], and prozostrodontians [3, 34, 78, 79, 88–91]. A posterior lower postcanine of *Candelariodon barberenai*, from the *Dinodontosaurus* AZ, was originally thought to be similar to *Aleodon* [92]; however, it has since been demonstrated that its dentition more closely resembles that of some prozostrodontians (e.g., *Prozostrodon*) and that the tooth in question is actually broken [93]. Another interesting taxon in this regard is the Middle Triassic *Cromptodon mamiferoides* (holotype PVL 3858) from Mendoza Province, Argentina [94]. It also has postcanines with a wide lingual cingulum, but unfortunately only the lower jaws are known. Nonetheless, the lower dentition of *Cromptodon* is clearly distinct from that of both *Aleodon* species. In *Cromptodon*, the lower postcanines have a sectorial labial crest with a conspicuous main cusp a, mesial cusp b and distal cusps c and d (cusp height = a>b>c>d), whereas in *Aleodon* the sectorial labial crest bears a conspicuous and bulbous main cusp, one very tiny mesial cusp, and two small distal cusps. The sectorial labial border of the lower postcanines of *Aleodon* is more similar to the morphology of *Chiniquodon* and ecteniniids, with the main cusp strongly posteriorly angled, than that of *Cromptodon*, in which the main cusps are more vertically positioned. In *Cromptodon*, the lingual cingulum is less developed, bears discrete cusps, and reaches its maximum width in the middle of the tooth row (pc3-pc4), then decreases in width in the posterior teeth. In contrast, in the well-preserved jaw MCN-PV 10338 of *Aleodon cromptoni* the lingual cingulum is the most conspicuous structure in all the postcanine teeth, being greatly expanded in both labiolingual and mesiodistal dimensions relative to the labial portion of the crown. The only known specimen of *Cromptodon* is extremely small (~27mm dentary length), contrasting considerably with the size sample for *Aleodon cromptoni*. Nonetheless, the presence of a canine-postcanine diastema in the former taxon would indicate a sub-adult or adult individual; therefore, the size difference between both taxa is notable and probably reflective of real taxonomic distinction.

The postcanine dentition of *Aleodon* is distinctive even when compared with gomphodont cynodonts. In the latter group, the upper postcanines bear a transverse crest, with a different position in each family, which is connected to the labial sectorial crest [80, 95–96]. The lower postcanines are usually subrectangular with a transverse crest, usually positioned on the mesial edge in traversodontids. Both upper and lower postcanine teeth develop conspicuous basins that, in conjunction with the crests, produce a mechanism for grinding [80, 96]. In *Aleodon*, the upper and lower postcanines have a sectorial labial crest and a lingual platform, capable of rudimentary crown-to-crown occlusion, without a transverse crest. Also, the upper and lower postcanine teeth of *Aleodon* exhibit a similar pattern of cusp and basin, differing from traversodontids in which the morphology of the upper postcanine teeth is clearly differentiated from that of the lowers.

The additional Brazilian specimens described above can also be referred to *Aleodon* based on the presence of postcanine teeth with a large lingual platform, and excluded from gomphodonts due to some combination of lengthy secondary palate, lack of tranverse crest on upper/lower postcanine teeth, and lack of maxillary platform. Although some of these specimens are poorly preserved, taken together they provide a nearly-complete view of various aspects of the osteology of *A*. *cromptoni*, such as dentition (MPDC-501-117; MCN-PV 10338, UFRGS-PV-0071-T, UFRGS-PV-0146-T), primary and secondary palates (UFRGS-PV-0071-T, UFRGS-PV-0125-T, UFRGS-PV-0274-T, UFRGS-PV-0146-T), dentary morphology (MCN-PV 10338, UFRGS-PV-0122-T, UFRGS-PV-0146-T), parietal and temporal morphology (UFRGS-PV-0122-T, UFRGS-PV-0125-T, UFRGS-PV-0146-T), and postcranial morphology (UFRGS-PV-0146-T).

### *Aleodon brachyrhamphus* and *A*. *cromptoni*

*Aleodon cromptoni* and *A*. *brachyrhamphus* are clearly closely-related taxa, and at present can only be distinguished based on features of the dentition. The publication of better preserved cranial specimens of *A*. *brachyrhamphus* (hopefully forthcoming) will be required to establish additional autapomorphies of these species. Here we discuss the key points of comparison currently available between *Aleodon cromptoni* and *A*. *brachyrhamphus*:

1. Although both species have a well-developed lingual platform on the upper and lower postcanine teeth, in *A*. *cromptoni* it is mesiodistally wider and labiolingually narrower than in *A*. *brachyrhamphus*. Consequently, in the latter taxon, the upper postcanine crowns are more transversely elongated.
2. Within the analyzed sample, the upper postcanine teeth of *A*. *cromptoni* have a distinct size increase between PC2 and PC3, as clearly shown in the holotype (Fig 4). By contrast, *A*. *brachyrhamphus* has a gradual increase in upper postcanine size between PC1 and PC4. Similar-sized specimens of both species (Fig 4) show different proportions in the absolute size of the postcanines. *A*. *cromptoni* has larger postcanine teeth in relation to *A*. *brachyrhamphus*, with the lingual platform two to three times wider than the sectorial labial crest in the middle and posterior postcanines.
3. The presence of an upper and lower diastema between canine and postcanine teeth is clearly evident in *A*. *brachyrhamphus*. This is related to the loss of the anteriormost postcanines during ontogeny, enlarging this space. In the holotype (UMZC T906) and referred (NHMUK PV R9390) specimens of *A*. *brachyrhamphus*, the diastema is conspicuous and the dentary exhibits the presence of resorbed bone in the shed alveoli. By contrast, *A*. *cromptoni* does not show an enlargement of the diastema during ontogeny. Both small (UFRGS-PV-0071-T; MMACR-PV-018-T) and large (MPDC-501-117, UFRGS-PV-0146-T, UFRGS-PV-0122-T) specimens lack a discrete space between the canine and postcanine tooth row, apparently independent of ontogenetic stage. Similar-sized specimens of *A*. *brachyrhamphus* (NHMUK PV R9390) and *A*. *cromptoni* (MPDC-501-117) show opposing conditions for this feature (Fig 4). In fact, in none of the specimens referred to *A*. *cromptoni* is there a conspicuous diastema. Therefore, it is possible that there are some differences in tooth replacement between these species, with limited or absent alveolar resorption in the anterior postcanine row of *A*. *cromptoni*.
4. The lower postcanines of the two *Aleodon* species are different in crown morphology. In *A*. *brachyrhamphus* (UMZC T906), the first three teeth are circular in occlusal view and they increase in width abruptly, with the fourth tooth considerably wider than the previous ones. From pc4 to pc7, they widen slightly and maintain almost constant width. They are more transversely elongated than in *A*. *cromptoni*, being about two times broader than long. However, in *A*. *cromptoni* the teeth remain circular in occlusal outline until pc5, and then have a less-developed lingual platform than in *A*. *brachyrhamphus*. The labial edge of the crown bears a main cusp in *A*. *brachyrhamphus*, whereas in *A*. *cromptoni* there is a sectorial crest with three discrete cusps descending in size posteriorly plus a tiny mesial one. The contact area between teeth of the same row is larger in *A*. *brachyrhamphus* than in *A*. *cromptoni*, due to the more elongated lingual platform in the former. As was mentioned in point (c), the smaller number of postcanines in *A*. *brachyrhamphus* would be related to the loss of anterior teeth, as clearly observed in UMZC T906, which has a large diastema and fewer postcanines than MCN-PV 10338.
5. In the holotype of *A*. *brachyrhamphus* (UMZC T906), only two lower incisors were described in each jaw [20], differing *a priori* from the condition of *A*. *cromptoni*, which has three incisors, a number common in probainognathians [32]. However, based on personal observations of UMZC T906 (by AGM), it seems to have three incisors in the left side and two in the right, with the left mediolabial border of the dentary broken off. Therefore, we assume that *A*. *brachyrhamphus* had three lower incisors, like *A*. *cromptoni*. Upper incisors are unknown in the holotype of *A*. *brachyrhamphus*. A referable specimen, NHMUK PV R9390, has four upper incisors, also as in *A*. *cromptoni*.
6. The upper postcanines of the *A*. *brachyrhamphus* specimen NHMUK PV R9390 have a longitudinal depression on the lingual face of the crown, such that there is a distinct ‘notch’ on the lingual platform (Fig 4A). This feature is more evident in postcanines in the mid-length of the row. The consistent presence of this character-state in *A*. *brachyrhamphus* specimens is uncertain, in large part because of poor preservation of the postcanine crowns in other specimens of this species. However, this feature is clearly not present in any specimen of *A*. *cromptoni*.
7. There is evidence of posterior sectorial teeth in the upper postcanine tooth row of the known sample of *A*. *cromptoni*, based on changes in shape of the alveoli of specimens UFRGS-PV-0071-T, UFRGS-PV-0125-T, and MMACR-PV-018-T. Unfortunately, the crown morphology of these teeth is unknown. The available lower jaws of *A*. *cromptoni* do not show such a conspicuous difference between transversely wide and sectorial teeth, especially in MCN-PV 10338 in which the postcanine row is well preserved (in specimen UFRGS-PV-0146-T the posterior portion of the lower tooth row is poorly preserved). In *A*. *brachyrhamphus* (e.g., NHMUK PV R9390) there are also sectorial postcanines at the rear of the upper tooth row. There is no clear evidence for lower sectorial teeth in the lower jaws of *A*. *brachyrhamphus*; although the posterior portions of the jaws in this taxon are generally poorly preserved.
8. Based on the size range present in the sample of *A*. *brachyrhamphus* from the Manda Beds (for those specimens deposited in the UMZC and NHMUK collections), it seems that the Brazilian *A*. *cromptoni* reached considerably larger skull size than its congener, as illustrated by specimens UFRGS-PV-0122-T, UFRGS-PV-0146-T, and MCN-PV 10338, and also by the tentatively-referred specimen GSN EN-3 from Namibia. In the sample of *A*. *brachyrhamphus* complete skulls are unavailable and, consequently, its total skull length is unknown. The holotype UMZC T906 represents one of the largest *A*. *brachyrhamphus* specimens; its jaw is smaller than the *A*. *cromptoni* jaw MCN-PV 10338 (see Fig 5), here considered a mid-sized specimen.

### Identification of the Namibian specimen GSN EN-3 as *A*. *cromptoni*

Abdala and Smith (2009) described an isolated skull with worn dentition (GSN EN-3) from the upper Omingonde Formation of Namibia as a specimen of *Aleodon* (Fig 16). This specimen was referred to *Aleodon* on the basis of its snout proportions, secondary palate length, and tooth morphology (specifically the labiolingually-expanded, ovoid-ellipsoid postcanine crowns). Abdala and Smith [13] did not identify this specimen to species (leaving it as *Aleodon* sp.), presumably because of its great geographic separation from the Manda Beds of Tanzania. The only possible difference they noted between this specimen and the Tanzanian material was the presence of a pineal foramen in UMZC T906 (holotype of *A*. *brachyrhamphus*), which was mentioned as present by Crompton [20]. However, they also noted that they could find no evidence of a pineal foramen when re-examining UMZC T906, and subsequent examination of that specimen by the authors of this paper (AGM and CFK) corroborates its absence.

The cranial morphology of GSN EN-3 is typical for *Aleodon*, with a tall, well-developed parietal crest and deep, robust zygomatic arches (Fig 16A and 16B), but the cranium alone does not exhibit any characters that permit identification as either *A*. *brachyrhamphus* or *A*. *cromptoni*. The postcanine tooth rows in this specimen are externally concave and point posterolaterally, terminating lateral to the subtemporal fossa in ventral view. The right postcanine tooth row preserves only one tooth (PC8), but also 11 alveoli. The left postcanine tooth row preserves 11 intact but badly worn crowns. The high degree of wear on these teeth makes species referral based on their morphology somewhat suspect, which is why we consider the following identification to only be tentative. With this noted, two features of the postcanine tooth row of GSN EN-3 are notable. One is the marked increase in tooth size between PC2 and PC3 (seen via alveolar dimensions on the right side) (Fig 16D and 16E). The other is the very broad, rounded lingual platform present in the best-preserved postcanine crowns (particularly the left PC6, 7, and 11). These are both characteristics that distinguish *A*. *cromptoni* from *A*. *brachyrhamphus*. Although it is possible that the Namibian material represents a third, endemic species of *Aleodon*, based on the above characters, the close geographic proximity of Rio Grande do Sul and Namibia during the Triassic, and the recent discovery of other characteristic elements of the *Dinodontosaurus* AZ in the upper Omingonde Formation (i.e., *Stahleckeria potens*; [46]), we consider it more likely that this specimen represents *A*. *cromptoni*.

### *Aleodon* versus *Chiniquodon*

Abdala and Giannini [14] revised the taxonomic composition of Chiniquodontidae, which at the time was restricted to the Middle-Late Triassic of Argentina and Brazil. They synonymized *Probelesodon* [73, 75, 82, 84] and the large-bodied taxon *Belesodon* [97] with the genus *Chiniquodon* [97], a taxon initially described from the Santa Maria Formation of southern Brazil. Previously stated differences between these taxa were explained as ontogenetic factors. Subsequently, material referred to *Chiniquodon* sp. was recognized in the *Santacruzodon* AZ of southern Brazil [98] and the upper Omingonde Formation of Namibia [13], and a new species, *C*. *kalanoro*, was described from the basal “Isalo II” beds of Madagascar [15]. As a result, the Chiniquodontidae currently includes: *Chiniquodon theotonicus* from the Chañares Formation of Argentina and *Dinodontosaurus* AZ of Brazil, *C*. *sanjuanensis* from the Ischigualasto Formation of Argentina, and *C*. *kalanoro* from basal “Isalo II” beds of Madagascar. Consequently, this genus spans a remarkably broad geographic and temporal range, extending across much of Gondwana and minimally from the Carnian to the Norian. *Aleodon* was excluded from the Chiniquodontidae by Abdala and Giannini [14], in contrast to the previous proposals of Hopson [26] and Hopson and Kitching [32].

The features diagnosing Chiniquodontidae include (see [14]): (a) robust zygomatic arch, flared laterally; (b) angulation between the ventral edge of the maxillary zygomatic process and the anteroventral margin of the jugal; (c) pterygoid flanges greatly elongated, ending in a thin projection; (d) long secondary osseous palate; and (e) postcanines with backwardly recurved main cusps, lacking cingula or with tiny lingual cingular cusps. The new described specimens of *Aleodon* clearly demonstrate that both *Chiniquodon* and *Aleodon* possess the same general cranial morphology, because they share the aforementioned traits (a-d), differing only in the dental feature (e). *Aleodon* is characterized by having non-sectorial postcanine teeth bearing a conspicuous, basined lingual platform, not sharply-recurved sectorial teeth throughout the postcanine row. The posteriormost upper postcanines of *Aleodon* are more sectorial in shape and bear recurved main cusps, however. The morphology of these teeth adds additional support for a close relationship between these taxa, as previously proposed by Hopson and Kitching [32] and Ruta et al. [33]. It is noteworthy how congruent the skull and jaw morphologies are between *Chiniquodon* and *Aleodon* despite their disparate dental forms.

**Fig 17:**
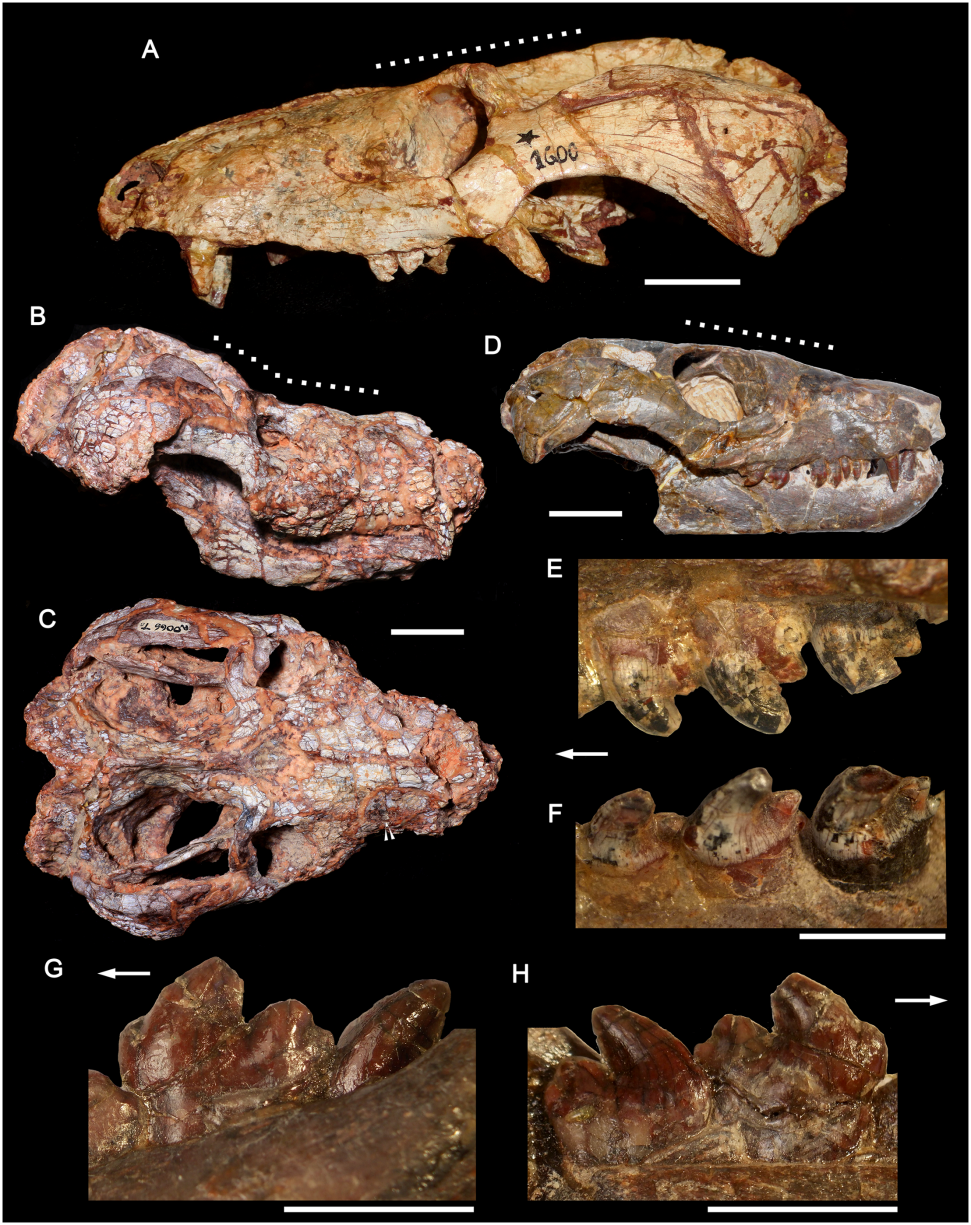
*Chiniquodon* from South America. Skull of specimen MCP-PV-1600-T (holotype of *Probelesodon kitchingi*) in lateral view (A). Skull and articulated jaws of specimen UFRGS-PV-1331-T (ex UFRGS-PV-0066-Tg) in lateral (B) and dorsal (C) views, and of specimen PVL 4444 in lateral view (D). Detail of posterior upper left postcanines of PVL 4674 in labial (E) and ventrolingual (F) views. Details of posterior lower left postcanines of PVL 4444 in labial (G) and lingual (H) views. Note the absence of a lingual platform. The arrow points to the mesial side. The dotted lines indicate the shape of the dorsal profile of the skull. Scale bar equals 20mm in A-D and 5mm in E-H.

Skull morphology is fairly consistent among the specimens referred to *A*. *cromptoni*, in which the skull length ranges from ~13cm (estimated from UFRGS-PV-0071-T) to 31cm (UFRGS-PV-0122-T). They have a short, wide snout, large zygomatic arches, robust jaws, and large canines (even in the smallest specimen). By contrast, the sample of specimens referred to *Chiniquodon theotonicus* is noticeably more heterogeneous. Specimens from the Chañares Formation from Argentina are usually smaller in size and more slenderly built, with long, narrow snouts. This morphotype is also observed in at least one specimen that comes from the *Dinodontosaurus* AZ, the holotype of *Probelesodon kitchingi* (MCP-PV-1600-T; [81, 82]) (Fig 17). Most other specimens of *Chiniquodon* from Brazil are poorly preserved [14, 97] and several referred specimens are still unprepared and in concretions. Independent of size, this morphotype bears a short snout and robust zygomatic arches (UFRGS-PV-0066-T, UFRGS-PV-1331-T (ex UFRGS-PV-0066-Tg by Abdala and Giannini [14]), with a marked concave outline between the parietal crest and the roof of the snout in lateral view (see dotted line in Fig 17). In contrast, the sample from the Chañares Formation and the specimen MCP-PV-1600-T from the *Dinodontosaurus* AZ have a more straight dorsal outline in lateral view, slightly tapering anteriorly (Fig 17). This outline in the *Chiniquodon* sample from Brazil is not considered a product of postmortem processes because it appears in several specimens, with different sizes and states of preservations. In both cases, robust or gracile forms, all the postcanine teeth are sectorial (Fig 17), distinguishing *Chiniquodon* from *Aleodon*. Further preparation of the extensive Brazilian sample of “*Chiniquodon*” would be greatly beneficial towards resolving the *Chiniquodon*-*Belesodon*-*Probelesodon* complex, and permit testing whether the aforementioned heterogeneity is attributable to taxonomy, ontogeny, and/or sexual dimorphism.

The referred sample for *Aleodon cromptoni* includes specimens reaching skull lengths greater than any known for *Chiniquodon*. The specimens UFRGS-PV-0274-T (27.7cm) and UFRGS-PV-122-T (31cm), both cited as the largest *Chiniquodon* individuals by Abdala and Giannini [14], are here considered to belong to *A*. *cromptoni*. Therefore, the largest specimen currently retained in *Chiniquodon* is the holotype of *Belesodon magnificus* (GPIT/RE/7112) at 26cm. The remaining specimens from Brazil and Argentina range from ~5 to ~20cm, with the most common size range between 10 and 15cm (see [14]).

### Phylogenetic placement of *Aleodon cromptoni*

**Fig 18:**
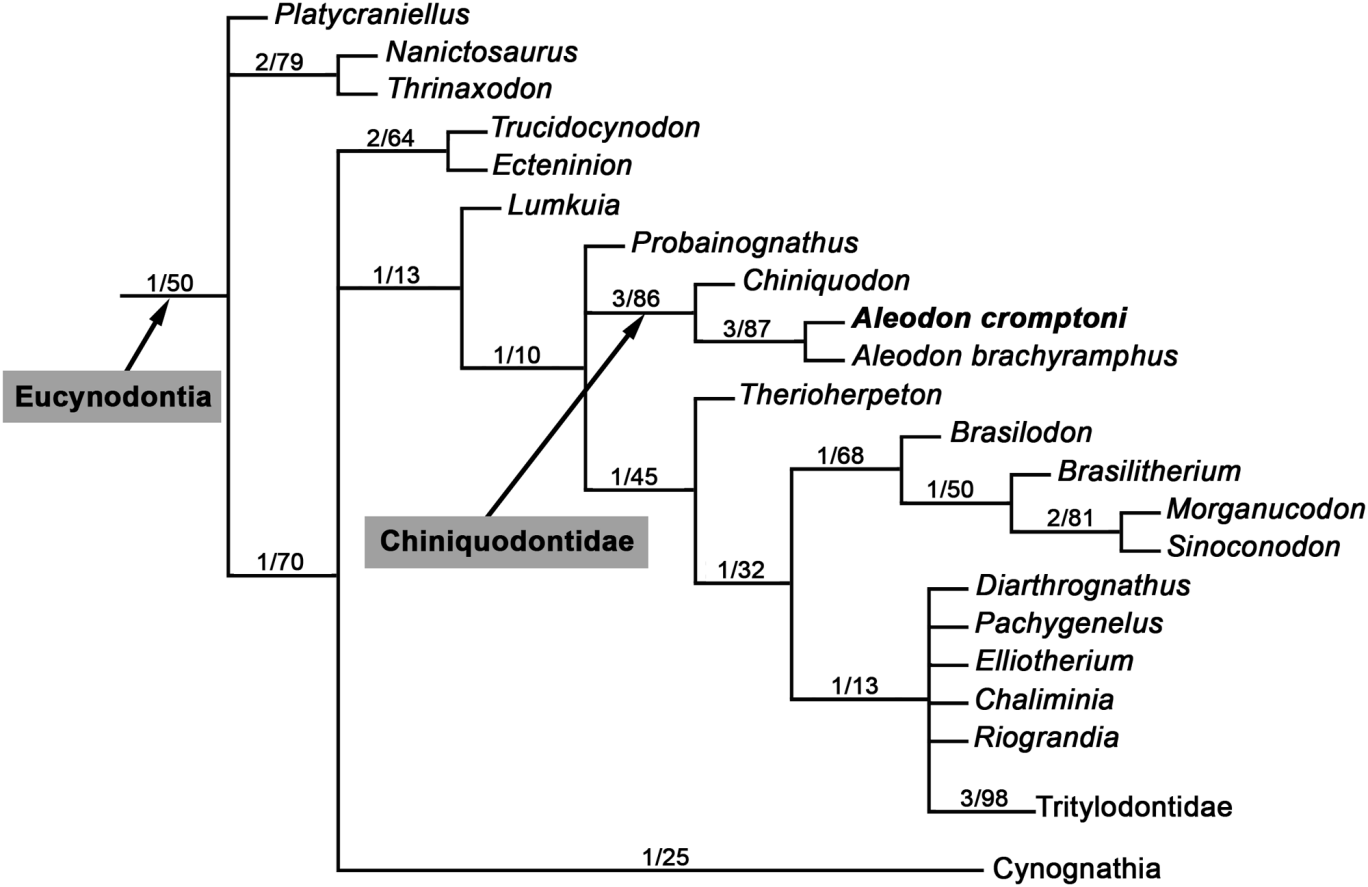
Phylogenetic relationships. Strict consensus tree of 75 most parsimonious trees, showing the phylogenetic position of *Aleodon cromptoni* sp. nov. within Chiniquodontidae. The numbers at nodes indicate Bremer support and Bootstrap values, respectively.

The cladistic analysis resulted in 75 most parsimonious trees of 529 steps (Ci = 0.35, Ri = 0.72). A selected part of the strict consensus tree is shown in Fig 18 (see S1 File for the complete tree), depicting *Aleodon cromptoni* as sister-taxon of *A*. *brachyrhamphus*, within Chiniquodontidae. Both species are supported by three ambiguous sinapomorphies: tooth-to-tooth contact because of widened postcanines (ch. 108(3)), bucco-lingually expanded upper postcanines (ch. 110(3)), and well developed lingual cingulum in lower postcanines (ch. 124(2)). Chiniquodontidae, including *Chiniquodon* plus *Aleodon* species, is supported by four ambiguous synapomorphies: large extranasal process of the premaxilla but not contacting nasal (ch. 2(1)), suborbital angulation between maxilla and jugal (ch. 26(1)), posterolateral end of maxilla forms right angle ventral to jugal contact (ch. 49(1)), and length of the secondary palate longer than the anterior border of orbit (ch. 52(2)). Although the inter-relationships of probainognathians are not the scope of the present contribution, the clade Chiniquodontidae and the placement of the new species (*A*. *cromptoni*) are well-supported within Probainognathia, with high Bremer support values (see Fig 18).

### Biostratigraphic implications

**Fig 19:**
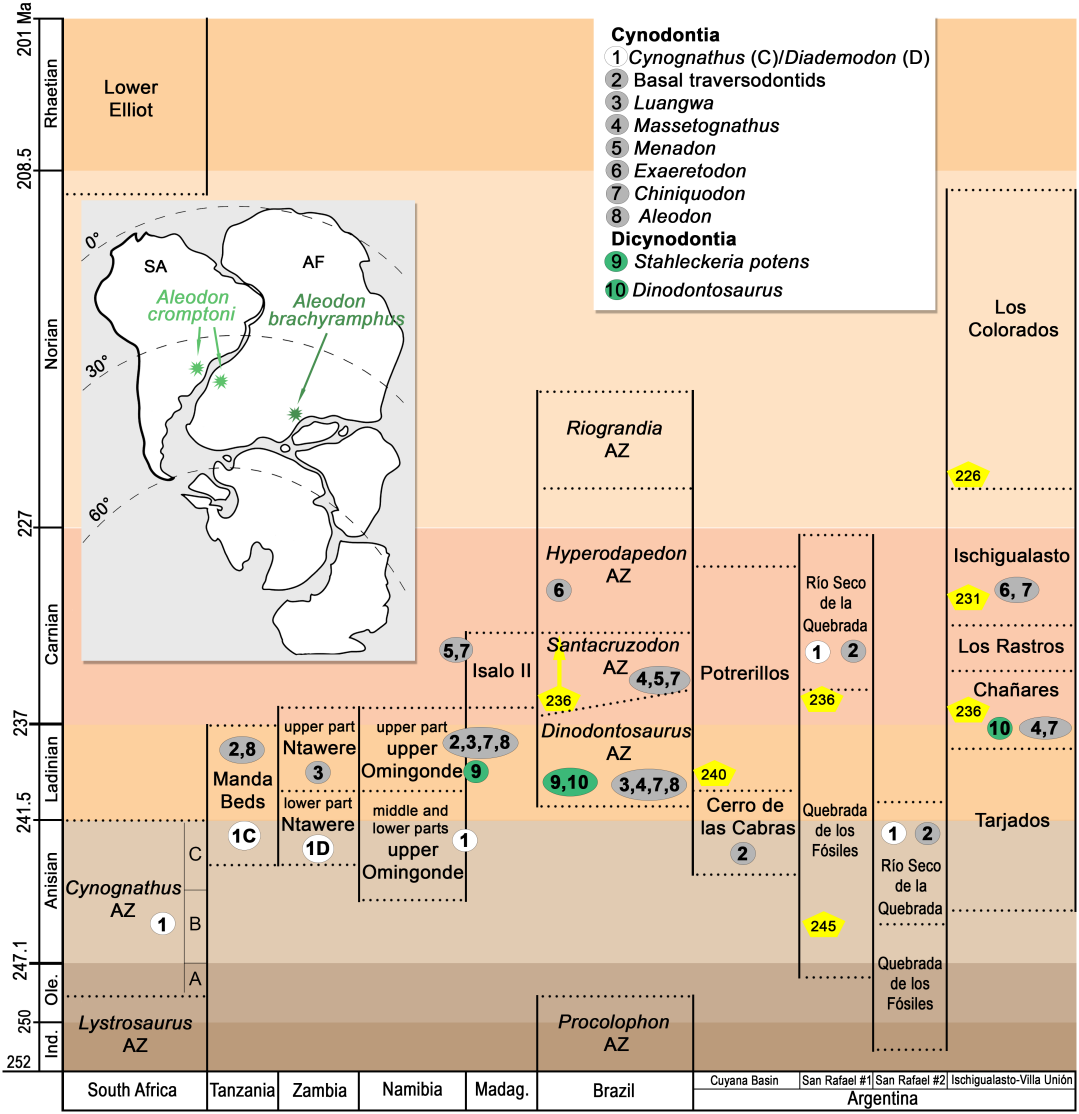
Biostratigraphy of selected Gondwanan Triassic units. Relevant therapsid content considering radiometric data and problematic implications among Gondwanan Triassic units, and accompanying paleomap (modified from Abdala and Smith [13]) during the Middle Triassic with the location of *Aleodon cromptoni* in southern Brazil and Namibia and *A*. *brachyramphus* in Tanzania. Abbreviations: AF, Africa; Ind., Induan; Madag., Madagascar; Ole, Olenekian; SA, South America.

In recent years, radioisotopic dating from various continental formations has substantially altered our understanding of Triassic chronology and the relative ages of the major tetrapod clades. In particular, these dates have cast doubts on the reliability of vertebrate biostratigraphy for establishing the age of Triassic faunal assemblages. However, interbasinal correlations were historically made at very rough levels, often establishing equivalency based on "family"-level taxa. More recently, new fieldwork and re-evaluation of historic specimens has revealed significant lower-level taxonomic overlap between cynodont faunas in South America and southern Africa (Fig 19). We discuss these biostratigraphic issues below, establishing context for the importance of *Aleodon* in Brazil.

#### South American correlations

Tetrapod associations from South America classically thought to represent the Middle Triassic (Anisian-Ladinian) are those from the Río Seco de la Quebrada Formation (Puesto Viejo Group; e.g., [10, 99–101]), Cerro de Las Cabras Formation (Cuyo Basin; e.g., [94, 101, 102]) and Chañares Formation (Ischigualasto-Villa Unión Basin; e.g., [44, 45, 103, 104]) from western Argentina and the *Dinodontosaurus* and *Santacruzodon* AZs (Santa Maria Supersequence; Paraná Basin; e.g., [35, 51, 52]) from southern Brazil. Recently, however, radioisotopic dates from the Chañares Formation have placed its typical vertebrate faunal association (from the upper half of the Lower Section; sensu [44]) in the early Carnian (236.1±0.6 Ma; [45]). Concomitantly, preliminary results from the *Santacruzodon* AZ (Santa Cruz Sequence) have also recovered an early Carnian age (236±1.5 Ma; [43]). These new radiometric data fit well into a unified biostratigraphic framework when the Paraná and Ischigualasto-Villa Unión basins are compared, as there are strong resemblances in synapsid and archosauromorph content between the *Dinodontosaurus* AZ and the Chañares Formation (e.g., [51, 52]). The *Santacruzodon* AZ of Brazil is situated between the *Dinodontosaurus* and overlying *Hyperodapedon* AZs [18, 35, 96]; the latter AZ has been biostratigraphically correlated with the Ischigualasto Formation in western Argentina, which is dated as late Carnian/early Norian, 231.4±0.3 to 225.9±0.9 Ma (e.g., [105]).

Although the new dates from Chañares and the *Santacruzodon* AZ shift the age of these faunas from Middle (Ladinian) to Late (Carnian) Triassic, the effects of this change on tetrapod correlation are relatively minor. The taxa such as traversodontid cynodonts, rhynchosaurs, and rauisuchians that dominate these assemblages were previously known from Carnian deposits, so a younger age for these faunas is not particularly surprising; the major novelties are the implications for the diversification of dinosauromorphs (e.g., [45]). By contrast, dates produced by Ottone et al. [106] for the Quebrada de los Fósiles Formation (San Rafael Group, Argentina) require a substantial overhaul of our understanding of Triassic biochronology if accurate. Ottone et al. [106] provided a SHRIMP ^238^U/^206^Pb age of 235.8±2.0Ma from an ignimbrite blow at the top of the Quebrada de los Fósiles Formation, placing the tetrapod association of the younger Río Seco de la Quebrada Formation in the beginning of the Carnian. A similar age had been obtained by Valencio et al. [107] based on a ^40^K/^40^Ar dating of 232±10Ma from an ignimbrite at the middle section of the Río Seco de la Quebrada Formation. This proposal is extremely problematic in terms of biochronology, because the Río Seco de la Quebrada Formation is the only South American unit bearing cynodonts (*Cynognathus crateronotus* and *Diademodon tetragonus*; [6, 9, 10]) typical of the *Cynognathus* AZ of the Karoo Basin in South Africa, an assemblage traditionally considered Early to Middle Triassic (Olenekian-Anisian) [7, 108–111]. *Cynognathus crateronotus* and *Diademodon tetragonus* have been widely-utilized in biostratigraphic correlations, since they achieved a broad Gondwanan distribution (Argentina, South Africa, Namibia, Zambia, Antarctica) during the Triassic and are relatively abundant in the fossil record [8, 10, 109, 110, 112]. Based upon the new radiometric dating from San Rafael Group and the traditionally older inferred age of the *Cynognathus* AZ of the Karoo (although see [113]), Ottone et al. [106] suggested two possible scenarios: (a) that the classical *Cynognathus* AZ of South Africa is much younger than previously thought or (b) that the biochron of *Cynognathus* and *Diademodon* is longer than previously thought, and thus their records in Argentina reflect the persistence of this fauna for nearly 20 million years. Both of these scenarios are highly problematic for Triassic faunal correlation (Fig 19). Other studies dating the top of the lower section of the Quebrada de los Fósiles Formation produced an age of ~245Ma [114, 115]. Based on these dates, the upper section of the Quebrada de los Fósiles Formation would have a span of about 9Ma (i.e., almost all of the Ladinian) [115].

The other tetrapod assemblage from the Triassic of Argentina is the traditional “Río Mendoza local fauna” of the Cerro Bayo of Potrerillos (Mendoza Province) [94, 101] referred to the Cerro de Las Cabras Formation (Uspallata Group, Cuyo Basin [102]; it was originally assigned to the Rio Mendoza Formation, see also [116]). The Cerro de Las Cabras Formation includes the dicynodont *Vinceria andina*, the traversodontid cynodont *Andescynodon mendozensis*, and the probainognathian cynodont *Cromptodon mamiferoides* [94, 101]. Due to the shared presence of *Vinceria*, this association was correlated with the Río Seco de la Quebrada Formation of the Puesto Viejo Group (e.g., [117–118]), with both considered as Anisian in age. Martinelli et al. [10] suggested that the tetrapod association of the Cerro de las Cabras Formation may prove to be somewhat younger than the Puesto Viejo association (as also suggested by [119]) based on the phylogenetic relationships of the traversodontids from Cerro de las Cabras and Puesto Viejo [96, 120] in combination with the presence of *Cynognathus* and *Diademodon* in the latter unit. Nevertheless, based on dating from the Cuyana Basin [121–122], the tetrapod association from the Cerro de Las Cabras Formation does appear to be Middle Triassic, possibly Anisian, in age (Fig 19).

Based upon the dates from the Puesto Viejo Group [106] and those from the Cerro de Las Cabras Formation (Cuyana Basin; [121–122]), biostratigraphic correlations between both units are quite problematic and do not fit with previous faunistic interpretations (e.g., [10, 117, 123]), especially because of the occurrence of *Cynognathus* and *Diademodon* in the supposed early Carnian Río Seco de la Quebrada Formation. As was briefly commented on by Sues [124] and Martinelli and Soares [4], the tetrapod composition of the Río Seco de la Quebrada Formation is highly bizarre when compared to other basins from South America (i.e., Ischigualasto-Villa Unión Basin in Argentina and Paraná Basin in Brazil). Even if the hypothesis of Ottone et al. [106] is accepted, in which the biochron of *Cynognathus* and *Diademodon* is longer than the African one, the absence of both genera in other well-sampled basins of Argentina and Brazil of early Carnian age would be a remarkably aberrant distribution (Fig 19). Also, the “evolutionary grade” of the entire tetrapod fauna, not only the cynodonts, is much more derived in the Carnian-Norian formations of the Ischigualasto-Villa Unión and Paraná basins. It is possible that the San Rafael Group may be younger than the typical *Cynognathus* AZ of the Karoo, but its proposed position as coeval to the Chañares Formation in Argentina and the *Dinodontosaurus* AZ in Brazil makes little sense in the evolutionary scenario of Triassic tetrapod communities. Determination of the exact stratigraphic provenance of *Cynognathus* and *Diademodon* within the Puesto Viejo Group, their stratigraphic correlation with the traversodontid *Pascualgnathus*, and new radiometric data are urgently needed to elucidate these biostratigraphic issues.

#### Brazilian *Aleodon* and African biostratigraphy

With regard to the continental African Triassic assemblages, several biostratigraphic problems have been also highlighted (e.g., [13]). The Karoo Supergroup has traditionally been used as the standard for correlating Permo-Triassic units worldwide, due to its vast temporal span, the abundance of collected fossils, and their taxonomic diversity (e.g., [111]). In particular, the *Lystrosaurus* and *Cynognathus* AZs of the Karoo have been fundamental to our understanding of the evolution of Early and Middle Triassic communities after the end-Permian mass extinction. More recently, studies on the *Cynognathus* AZ have permitted the recognition of three subzones based initially on temnospondyl amphibian taxa [110, 125] and subsequently reinforced by other faunal elements [126]. For cynodonts, the oldest Subzone A includes *Cynognathus*, *Langbergia*, and indeterminate cynodonts with allotherian-like teeth [127] and has traditionally been considered upper Olenekian. Subzone B corresponds to the ‘classical’ *Cynognathus* AZ and besides *Cynognathus* and *Diademodon* also includes *Trirachodon*, *Bolotridon* and *Lumkuia* [32, 109] and has traditionally been considered lower Anisian. Finally, in the youngest Subzone C [126, 128–130] the dominant cynodont is *Cricodon* [126], with rarer records of *Cynognathus* and *Diademodon*. This subzone has traditionally been considered upper Anisian. Thus, the *Cynognathus* AZ as a whole has been thought to span from the upper Olenekian (late Early Triassic in age) to the upper Anisian (mid Middle Triassic in age).

Other well-studied African basins with Middle Triassic tetrapod assemblages include the Manda Beds of the Ruhuhu Basin, Tanzania [20–21], the upper Omingonde Formation of the Otiwarongo Basin, Namibia [13, 131–133], and the Ntawere Formation of the Luangwa Basin, Zambia [11, 134–135]. Among cynodonts, *Cynognathus*, *Diademodon* and trirachodontids have been used for correlations among these rocks; although the non-Karoo basins from continental Africa exhibit a combination of taxa that makes it difficult to straightforwardly correlate these units (e.g., [13, 113]).

The discovery of the genus *Aleodon* in the *Dinodontosaurus* AZ of Brazil bears directly on these biostratigraphic questions. *Aleodon brachyrhamphus* comes from the Lifua Member of the Manda Beds in Tanzania [20–21] where it is found in association with gomphodont cynodonts: the trirachodontid *Cricodon metabolus* [20] and the traversodontids *Scalenodon angustifrons* [20], *Mandagomphodon hirschsoni*, and *M*. *attridgei* [120, 136] (Fig 19). Recently, new stratigraphically controlled collections have subdivided the Lifua Member into lower and mid-upper sections, but these faunal associations hold: the higher section includes *Aleodon*, trirachodontids and traversodontids, whereas the lower includes *Cynognathus* (e.g., [112, 137]).

In Namibia, in the upper Omingonde Formation, GSN EN-3 (the specimen here tentatively referred to *Aleodon cromptoni*; Fig 16) was reported exclusively associated with traversodontids (*Luangwa* sp. and an indeterminate form) and *Chiniquodon* sp. in the upper levels of the unit. Significantly, *Aleodon* in Namibia is not found in association with *Cynognathus* and *Diademodon*, because these taxa occur only in the middle and lower portions of the upper Omingonde Formation [13]. Abdala and Smith [13] suggested the distribution of these taxa indicates that the middle and lower levels can be correlated with the *Cynognathus* AZ of the Karoo Basin and that the upper level can be correlated with the upper portion of the Ntawere Formation in Zambia (based on the shared presence of the traversodontid *Luangwa*; [11, 135]) and part of the Santa Maria Formation in Brazil (based on *Luangwa sudamericana* and *Chiniquodon theotonicus*; [12]). In addition, the presence of *Stahleckeria potens* [13] and a large rauisuchian [138] in the upper Omingonde Formation strengthens its relationships with the *Dinodontosaurus* AZ of southern Brazil.

The presence of *Aleodon* in Brazil permits a biostratigraphic correlation with the upper levels of the Manda Beds in Tanzania [112, 137] and the upper Omingonde Formation in Namibia, and its association with the basal traversodontid *Luangwa sudamericana* extends this correlation to the upper horizons of the Ntawere Formation in Zambia (Fig 19), as was previously argued by Abdala and Sá-Teixeira [12].

#### *Aleodon cromptoni*, *Luangwa sudamericana* and the *Dinodontosaurus* AZ

**Fig 20:**
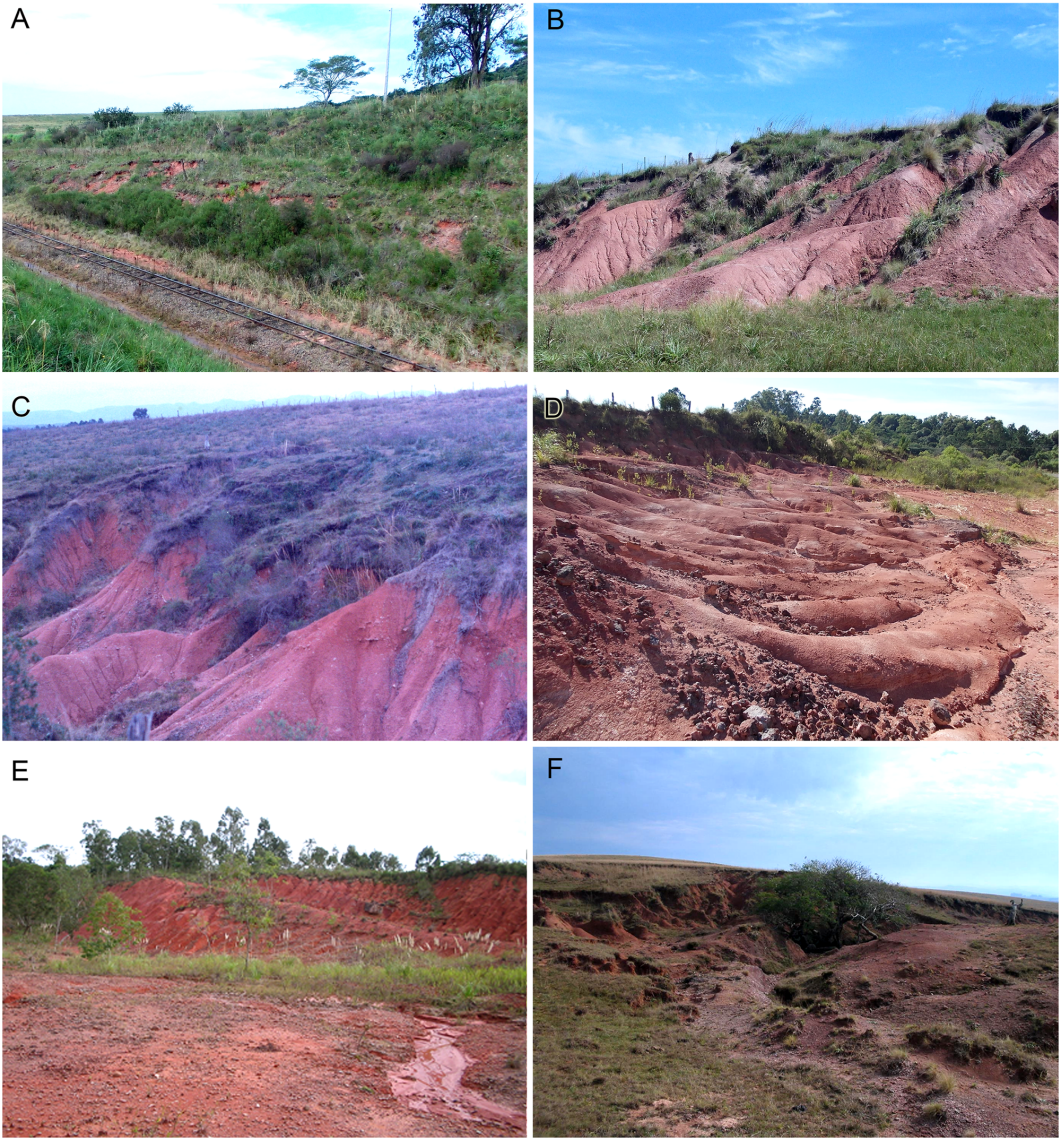
Selected outcrop of the *Dinodontosaurus* AZ of the state of Rio Grande do Sul, Brazil (see Fig 1 for geographical location of the outcrops). A, Vale Verde (in 2016); B, Sanga Menezes in Bom Retiro region (in 2015); C, Sanga Pinheiro (in 1996); D, Cortado Site (in 2013); E, Posto site of Dona Francisca (in 2005); F, Sanga Baum in Chiniquá region (in 2000).

The *Dinodontosaurus* AZ of the Pinheiros-Chiniquá Sequence, Santa Maria Supersequence [35, 42], is presumably its oldest faunal association [51] with several outcrops in Rio Grande do Sul (Figs 1 and 20). Below this sequence the Triassic fossil content is confined to the *Procolophon* AZ of the Sanga do Cabral Supersequence of Early Triassic age [139–140], both separated by a marked disconformity from the Gondwanides II paroxysm [42]. The *Dinodontosaurus* AZ, a denomination coined by Lucas [141], was traditionally known as the Therapsid Cenozone, restricted to the Alemoa Member of the Santa Maria Formation and placed below the Rhynchosauria Cenozone (defined as the *Hyperodapedon* AZ in [141]). Although many of the taxonomic and biostratigraphic proposals advanced by Lucas [137] have not stood up to subsequent scrutiny (see comments in [49, 142]), this terminology was maintained in latter contributions (e.g., [34, 35, 52]). Originally, the Therapsid Cenozone was established based on the Chiniquá and Pinheiros Local Faunas [51, 143–144] including several outcrops with a diverse fauna dominated by the dicynodont *Dinodontosaurus* and the cynodonts *Massetognathus* and *Chiniquodon* (see [52]). Other, less abundant components of the Chiniquá and/or Pinheiro regions (see typical outcrops in Fig 20) are the dicynodont *Stahleckeria* [57, 58, 145, 146], the cynodont *Traversodon* [97, 147], and the archosauriforms *Prestosuchus*, *Spondylosoma*, and indeterminate proterochampsians [65, 69, 148–150]. In recent decades, several new outcrops were discovered yielding typical components of the *Dinodontosaurus* AZ such as Cortado [41, 151], Linha Várzea [152], and Dona Francisca (Posto outcrop) sites [54, 153–154]. Langer et al. ([52], see also [155]) provided a detailed account of taxa and localities of the *Dinodontosaurus* AZ. In Table 3 (see also S1 File) we update this information including new records and taxonomic reappraisals.

**Table 3:** Occurrence of taxa and main localities of the *Dinodontosaurus* AZ in the state of Rio Grande do Sul, southern Brazil (see Suppl. Info. for references of each occurrence).

	Cynodontier Sanga	Weg Sanga	Baum Sanga	Rincão do Pinhal	Linha Várzea	Dona Francisca (Posto Site—Antonini Bortolin Site)	Cortado	Pinheiro region	Bom Retiro region (Sanga Pascual / Sanga Hintz)	Porto Mariante 1	Vale Verde	Indet. Locality
Taxon	West region (Chiniquá área)			Central region				East region				
**DICYNODONTIA**												
***Dinodontosaurus* sp.**	**X**	**X**	**X**	**X**	**X**	**X**	**X**	**X**	**X**	**X**	**X**	
***Stahleckeria potens***			**X**					**X**	**X**			
**CYNODONTIA**												
***Massetognathus* (*M*. *ochagaviae*, *M*. *pascuali*, *M*. sp.)**				**X**	**X**	**X**	**X**	**X**	**X**		**X**	
***Traversodon stahleckeri* /? *T*. *major***	**X**		**X**									
***Protuberum cabralensis***				**X**			**X**					
***Luangwa sudamericana***						**X**			**X**		**X**	**X**
***Chiniquodon theotonicus***	**X**	**X**	**X**	**X**	**X**			**X**				
***Aleodon cromptoni***							**X**	**X**	**X**		**X**	
***Bonacynodon schultzi***								**X**				
***Candelariodon barberenai***							**X**	**X**				
***Protheriodon estudianti***						**X**						
PROCOLOPHONIA												
***Candelaria barbouri***							**X**	**X**				
ARCHOSAUROMORPHA												
***Brasinorhynchus mariantensis***										**X**		
**Proterochampsidae indet.**								**X**				
***Barberenasuchus brasiliensis***							**X**					
***Archeopelta arborensis***			**X**									
***Decuriasuchus quartacolonia***						**X**						
***Prestosuchus chiniquensis***	**X**	**X**	**X**		**X**	**X**		**X**	**X**		**X**	
***Spondylosoma absconditum***	**X**		**X**									

**Fig 21:**
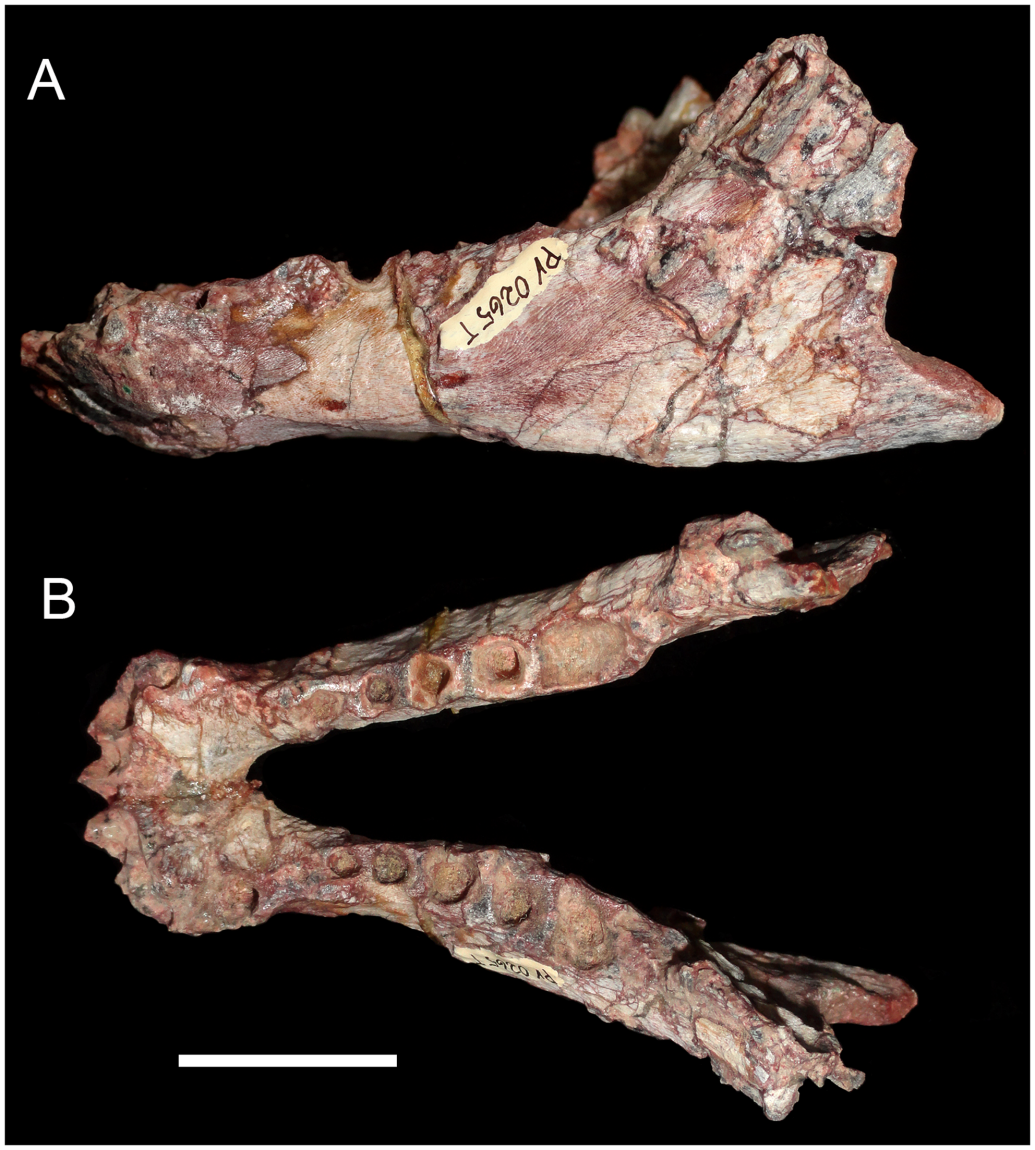
Specimen UFRGS-PV-0265-T of *Luangwa* sp. (Traversodontidae) from Vale Verde locality, Rio Grande do Sul State, Brazil. Lower jaws in lateral (A) and dorsal view (B). Scale bar equals 20mm.

The recognition of *Luangwa sudamericana* in the Triassic of Brazil, closely related to *Luangwa drysdalli* from Zambia, opened new queries about the temporal significance of this taxon [12] and its age and possible inclusion into the *Dinodontosaurus* AZ. This taxon suggested the occurrence of a hidden faunal association, older than the known typical fauna of the *Dinodontosaurus* AZ. Problematically, the precise location of the holotype specimen of *L*. *sudamericana* is unknown, but comes from an area between Vera Cruz and Candelária cities [12]. After that contribution, there were brief reports of new *Luangwa* material coming from the Dona Francisca region (Antonini Bortolin site; [156–157]), but these specimens remain unpublished. In addition, the specimen UFRGS-PV-0265-T (Fig 21; see description in S1 File) can be referred to *Luangwa* and it comes from the same outcrop as the holotype of *Aleodon cromptoni*. Another possible specimen deposited in the UFRGS-PV collection that can be referred to *Luangwa* is UFRGS-PV-0140-T, found in the Sanga Hintz (Bom Retiro region, Candelária), but this specimen needs further preparation to be certain of this referral. As previously mentioned, at least one specimen of *A*. *cromptoni* (UFRGS-PV-0146-T) comes from Sanga Pascual, located nearby Sanga Hintz.

The “Mariante rhynchosaur” (from Porto Mariante 1 locality), recently formally named as *Brasinorhynchus mariantensis* by Schultz et al. [158], was also considered a species that suggests the existence of an older, poorly sampled fauna [50, 52, 158–159]. This assumption is supported by the inclusion of *Brasinorhynchus mariantensis* within typical Middle Triassic stenaulorhynchine rhynchosaurs, forming a clade with *Stenaulorhynchus stockleyi* [160] from the Manda Beds (Tanzania) [158].

Taking into consideration the new Brazilian *Aleodon* and the already known *Luangwa sudamericana* and *Brasinorhynchus mariantensis*, there appears to be evidence for an older faunal assemblage in the Pinheiros-Chiniquá Sequence, mixed within the traditional *Dinodontosaurus* AZ. However, there are several problems with this idea: (1) several records from traditional outcrops lack stratigraphic control; therefore, the stratigraphic and taxonomic characterizations of two possible subzones or zones become impossible. For example, the lack of this information precludes the recognition of two subzones or fossiliferous levels in the same outcrop. (2) The geographical locations of several specimens are not always precise or known. (3) Although the fossiliferous sites are located in a fairly restricted region of the state of Rio Grande do Sul, lateral correlations are impossible to perform and there is no absolute dating at fossiliferous localities. (4) Several tetrapod specimens referred to classical taxa (e.g., *Dinodontosaurus*, *Massetognathus*, *Chiniquodon*) are often partially preserved and/or not prepared, hampering the recognition of diagnostic features. Traditionally, the presence of any *Dinodontosaurus*-like kannemeyeriiform remains was enough to place strata in the *Dinodontosaurus* AZ, but these specimens may not be robustly referable to *Dinodontosaurus* on a strict apomorphy basis. (5) The taxonomic referral of numerous *Dinodontosaurus* AZ specimens remains controversial, as should be evident from the *Aleodon* case detailed here.

The association of *Aleodon* and *Luangwa*, their relationship with other Gondwanan faunas (see Fig 19), and the stenaulorhynchine affinities of *Brasinorhynchus* are likely indicative of the occurrence of an older subzone within the *Dinodontosaurus* AZ, although this is admittedly based on a ‘stage of evolution’ argument. This older subzone can be characterized by the presence of *Aleodon*-*Luangwa*-*Brasinorhynchus*. However, with available stratigraphic data we cannot determine their precise temporal span. Langer et al. (Fig 2 in [52]) suggested that the Sanga Baum (Chiniquá region) and the Sanga Pascual and Bom Retiro area (see S1 File for references of these localities) could have a younger faunal association than the remaining *Dinodontosaurus* AZ localities (e.g., Cynodontier Sanga, Weg Sanga, Pinheiro region, Dona Francisca, Cortado sites) due to the presence, in the former, of the toothless dicynodont *Stahleckeria potens*. However, due to the aforementioned problems this hypothesis is difficult to test.

For example, in the Pinheiro region, without precise biostratigraphic control, *Aleodon* is associated with *Dinodontosaurus*, *Massetognathus*, *Chiniquodon*, and *Prestosuchus* (see S1 File). Therefore, for this locality the possibilities are: (1) both subzones are represented and the fossil sample is mixed; (2) the biochron of the taxa is not strictly bounded and the *Dinodontosaurus* AZ spans from the Ladinian to the early Carnian with a succession of forms that do not necessarily define two clearly distinctive subzones.

In Chiniquá region, there is no record of *Aleodon*, *Luangwa*, or *Brasinorhynchus* at the moment. Of note, in that region was found the doswelliid *Archeopelta arborensis*, which is phylogenetically closer to *Tarjadia ruthae* [161] from the lower member of the Chañares Formation (e.g., [44, 162]).

In the area of Dona Francisca (Figs 1 and 20) there is a predominance of *Dinodontosaurus*, *Massetognathus*, and the rauisuchians *Decuriasuchus* and *Prestosuchus* [154, 163–164]. At present there is no record of *Aleodon* and there are mentions of possibly *Chiniquodon* and *Luangwa* based only on abstract meetings [156–157] and have not been confirmed by thorough description.

Outwardly the genus *Dinodontosaurus* seems to be present in most localities (e.g., [41, 54, 152]), although several specimens merit further taxonomic analysis. With the taxonomic base at hand, this suggests a lengthy temporal span of the genus. In fact, a critical aspect we noted is that during fieldwork prospections the presence of dicynodont elements (skulls and/or postcranial elements) is ambiguously thought as indicative of the *Dinodontosaurus* AZ.

**Fig 22:**
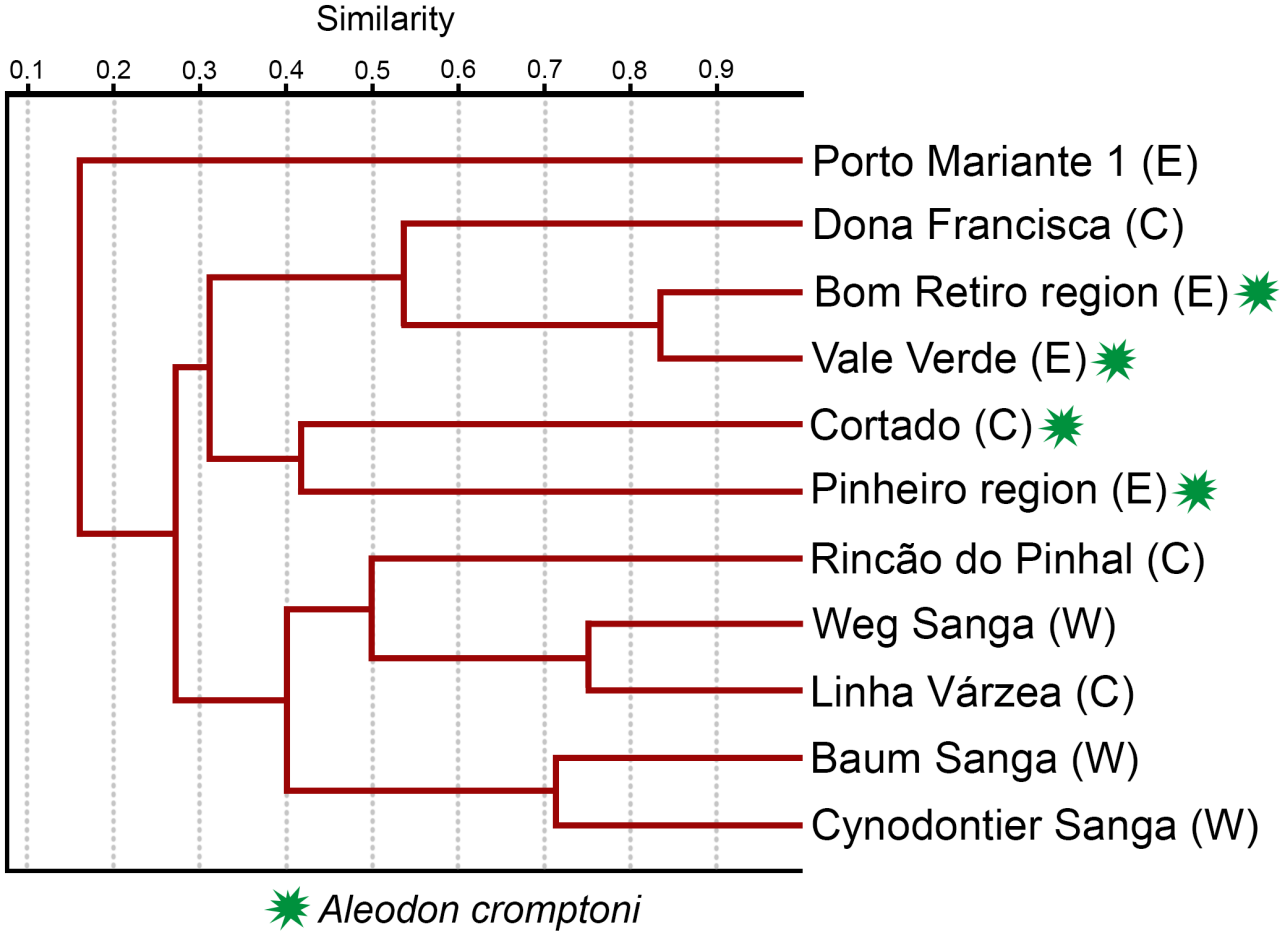
Dendrogram of main localities of the state of Rio Grande do Sul yielding fossil tetrapods of the *Dinodontosaurus* AZ (see data matrix in S1 File). Cophenetic correlation coefficient = 0.8474. (W), and (C), and (E) refers to the West, Central, and East portions, respectively, of the state of Rio Grande do Sul.

The distribution of taxa in Table 3 visually suggests that the taxa of the *Dinodontosaurus* AZ are randomly distributed and that there is a predominance of taxa indicative of a presumably older age (i.e., *Aleodon*, *Luangwa*, and *Brasinorhynchus*) in the east portion of the Triassic outcrops. Although not conclusive, it would suggest that at these areas the two subzones appear in the same outcrops or region, and the lack of stratigraphic control of collected fossils biases the recognition of both fossil assemblages. The obtained dendrogram, based on the analysis of 11 localities versus 19 taxa (S1 File), shows two main clusters, with two main sub-clusters in each one (Fig 22). The outcrops of the Chiniquá region are clustered together with Rinção do Pinhal and Linha Várzea sites, by the absence of *Aleodon* and *Luangwa*. The position of Porto Mariante 1 on the dendrogram is due to its incomplete fossil record. Cortado site and Pinheiro region are clustered together, being more similar than Dona Francisca, Bom Retiro region, and Vale Verde, which are clustered together. Particularly, Cortado site and Pinheiro region have the higher number of taxa from the center and east regions. As aforementioned, the dissimilarity of the west and east regions is conspicuous, and localities from the central region seems to represent “transitional” faunas. This dissimilarity would be an artifact result of selective effort for paleontological collections and thus a bias in this analysis. However, if the *Dinodontosaurus* AZ includes in fact mixed faunal elements of two biozones (or subzones) the present analysis would be indicating that these are more likely to be represented in the central and east outcrops, due to the shared presence of *Aleodon* and *Luangwa*, two cynodonts that are supposed to indicate an older age. Moreover, the amount of unpublished, unprepared specimens, and the lack of accurate geographical and stratigraphical data are also issues that hamper a better resolution of the similarities among Triassic outcrops in Rio Grande do Sul. In addition, the Triassic rocks in the center of the state of Rio Grande do Sul have different thickness along their east-west distribution. The younger portions of the Triassic package and its greater thickness are located in the central portion of the state [165]. To the east and west, the Triassic package thins out and the basal most portions (i.e., oldest rocks) of the Santa Maria Supersequence are exposed. Consequently, there are several outcrops of the *Dinodontosaurus* AZ on the east and west sides (see Figs 1 and 22 and Table 3); however, in the west side, the basal most portions are partially covered by the Guará Formation (e.g., [42, 165]).

#### *Dinodontosaurus* and *Santacruzodon* AZs

The *Santacruzodon* AZ was recently included within the Santa Cruz Sequence (see historical and geological review in [35]), being considered “intermediate” between the *Dinodontosaurus* (older) and the *Hyperodapedon* (younger) AZs. Representative outcrops of this AZ are geographically confined to the east portion of the Triassic rocks, in the municipalities of Venâncio Aires, Santa Cruz do Sul, and Vera Cruz [35, 98]. The fossil content of the *Santacruzodon* AZ is limited when compared to other assemblages from the Santa Maria Supersequence. This is represented by the traversodontids *Santacruzodon hopsoni* [93], the most common form, *Menadon besairiei* [18], and *Massetognathus* sp. [98], the probainognathians *Chiniquodon* sp. [98] and *Santacruzgnathus abdalai* [3], the archosauriform *Chanaresuchus bonapartei* [166], the rauisuchian *Dagasuchus santacruzensis* [167], and few unpublished fragmentary cranial remains of tusked dicynodonts, compared in morphology to *Dinodontosaurus* (Pers. Obs.).

**Fig 23:**
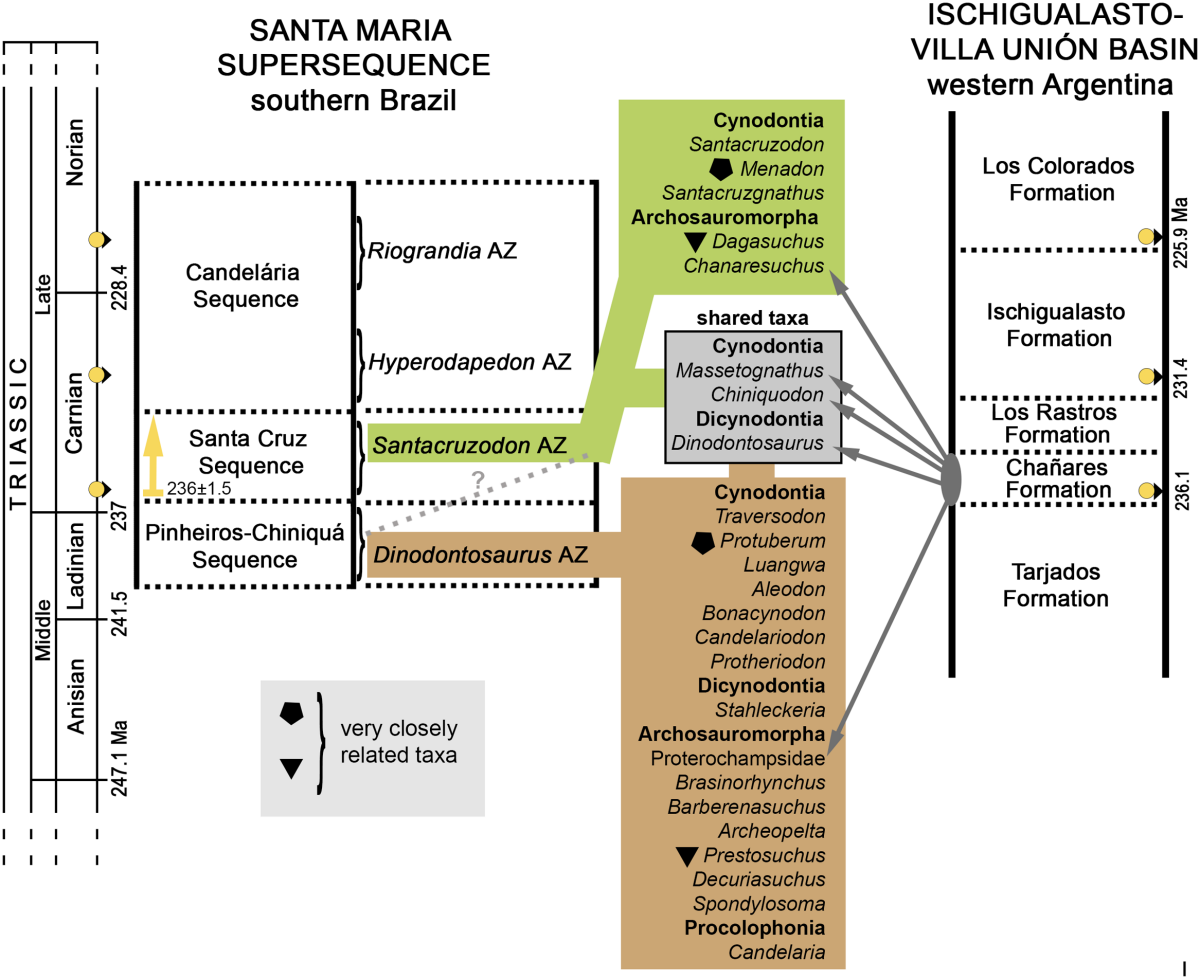
Biostratigraphy of the *Dinodontosaurus* and *Santacruzodon* AZs. Tetrapod fossil comparisons and shared taxa with the Chañares Formation of western Argentina. The ages of the column follow Gradstein et al. [36]. The radiometric dating of 236.1, 231.4 and 225.9 Ma on the Ischigualasto-Villa Unión Basin column correspond to the first half of the Chañares Formation [45], the base of the Ischigualasto Formation, and the base of Los Colorados Formation [105], respectively, and the one in the *Santacruzodon* AZ follows Philipp et al. [43].

The shared presence of *Massetognathus* sp., *Chiniquodon* sp., and *Chanaresuchus*; the close phylogenetic relationship of *Santacruzodon* with *Massetognathus* [120, 168] and of *Dagasuchus* with *Prestosuchus* [167]; and the presence of *Dinodontosaurus*-like specimens are significant points that do not clearly demonstrate the distinctiveness of the *Santacruzodon* AZ compared to the *Dinodontosaurus* AZ (Fig 23). In addition, the traversodontid *Menadon*, apparently restricted to the *Santacruzodon* AZ in Brazil (and also recorded in Madagascar), also has a close phylogenetic relationship with *Protuberum*, from the *Dinodontosaurus* AZs, being positioned basal to the former taxon in some phylogenetic hypotheses (e.g., [120, 168]). To this scheme, the age obtained for the *Santacruzodon* AZ as early Carnian (236±1.5 Ma; [43]) is the same that the typical faunal association of the lower section of the Chañares Formation [44, 45]. However, traditional correlations have been done directly between the *Dinodontosaurus* AZ with the Chañares Formation (e.g., [51, 143]). The taxa recovered in both Brazilian AZs and the Chañares Formation have an interdigited pattern and only further detailed taxonomic studies and stratigraphically controlled specimens, mainly from Brazil, will elucidate these issues summarized in Fig 23. As such, the standard idea that the *Santacruzodon* AZ (and the Santa Cruz Sequence as a whole) is a clearly distinctive tetrapod assemblage from the *Dinodontosaurus* AZ requires reevaluation with new paleontological data.

## Conclusions

We describe a new species of *Aleodon*, *A*. *cromptoni*, based on several specimens from the *Dinodontosaurus* AZ of the Pinheiros-Chiniquá Sequence, Santa Maria Supersequence of Rio Grande do Sul, Brazil. One specimen, GSN EN-3, from the upper Omingonde Formation of Namibia is also tentatively referred to this species. *Aleodon cromptoni* is clearly differentiated from *A*. *brachyrhamphus* from the Manda Beds of Tanzania [20] on the basis of its upper and lower postcanine dentition, and includes individuals that reached larger skull sizes (up to 31cm skull length) than the known sample of the Tanzanian species. Inclusion of this taxon in a phylogenetic analysis provides further evidence for close relationship between *Aleodon* and *Chiniquodon*, as originally proposed by Hopson and Kitching [25], and we include both taxa in the family Chiniquodontidae. Both genera share a very similar cranial morphology despite conspicuous differences in their dentition. The recognition of *Aleodon* in Brazil, hidden in most collections with cynodonts from the *Dinodontosaurus* AZ, and the comparisons with the known sample of *Chiniquodon* from Argentina and Brazil brings to light taxonomic problems with the latter genus that require additional study. The presence of *Aleodon cromptoni* in the *Dinodontosaurus* AZ reinforces its similarity to the upper Omingonde Formation of Namibia, which also share the dicynodont *Stahleckeria potens* [46] and the cynodonts *Luangwa* sp. and *Chiniquodon* sp. [13], making this one of the most strongly-correlated Gondwanan Triassic faunal associations. Biostratigraphic issues in South America are still controversial and further taxonomic refinements and new radiometric and geological data are required to resolve the biostratigraphy for the Middle-Late Triassic of Gondwana.

## Supporting information

S1 File In this file are included phylogenetic analysis details, additional figures, description of *Luangwa* (UFRGS-PV-0265-T), a table with references of fossil occurrences in different localities of the *Dinodontosaurus* AZ of Brazil, comments on fossiliferous localities, the data matrix of the cluster analysis, and Jaccard similarity indices. (DOCX)

S2 File Modified data matrix. (TXT)
