# Supplementary material for: The African cynodont Aleodon (Cynodontia, Probainognathia) in the Triassic of southern Brazil and its biostratigraphic significance
Source: PLoS One. 2017 Jun 14;12(6):e0177948. doi: 10.1371/journal.pone.0177948 (PMC5470689; doi:10.1371/journal.pone.0177948)
Supplement: S1 File — (DOCX) [file pone.0177948.s001.docx]

Supporting Information 1 for Martinelli et al. “**The African cynodont *Aleodon* (Cynodontia, Probainognathia) in the Triassic of southern Brazil and its biostratigraphic significance**”

In this file are included the following sections: (1) Phylogenetic analysis; (2) additional figures; (3) description of *Luangwa*, specimen UFRGS-PV-0265-T**;** (4) table with references of fossil occurrences in different localities of the *Dinodontasaurus* AZ of Brazil (Table1); (5) comments on fossiliferous localities (i.e., outcrops); (6) the data matrix of the cluster analysis (Table 2), and Jaccard similarity indices (Table 3).

**(1) Phylogenetic Analysis**

**(a)** Changes in character-states for some taxa of the data matrix of Ruta et al. (2013):

**Character 25.** Zygomatic arch dorsoventral height: slender (0), moderately deep (1), very deep (2). *Chiniquodon*, 0→1. *Aleodon brachyramphus*, ?→1.

**Character 26.** Infraorbital process: absent (0), suborbital angulation between maxilla and jugal (1), descendant process of jugal (2). *Aleodon brachyramphus*, 2→1.

**Character 44.** Incisive foramen: absent (0), not closed (1), posteriorly closed by maxilla (2), completely enclosed by premaxilla (3). *Probainognathus*, 3→2.

**Character 61.** Boss/crest anterior to the interpterygoid vacuity: reduced or absent (0), well developed (1). *Aleodon brachyramphus*, 1→?.

**Character 108.** Postcanine occlusion: absent (0), unilateral without forming a consistent pattern between upper and lower teeth (1), precise unilateral occlusion (2), tooth-to-tooth contact because of widened postcanines (3). *Aleodon brachyramphus*, ?→1.

**Character 111.** Posterior postcanines with strongly curved main cusp: absent (0), present (1). *Aleodon brachyramphus*, ?→1.

**Character 113.** Lower postcanine roots: single (0), incipiently bifurcated (1), divided (2), divided with multiple cusps (3). *Aleodon brachyramphus*, ?→0.

**Character 114.** Position of upper transverse cusp row on crown: on anterior half of crown (0), midcrown almost to posterior margin (1), at posterior margin (no posterior cingulum) (2). *Aleodon brachyramphus*, ?→(-).

**Character 115.** Central cusp of upper transverse row: absent (0), midway between buccal and lingual cusps (1), closer to lingual cusp (2), close to labial cusp (3). *Aleodon brachyramphus*, 0→(-).

**Character 120.** Upper anterior transverse (cingulum) ridge: low (0), high (1). *Aleodon brachyramphus*, ?→(-). *Chiniquodon*, ?→(-). *Probainognathus*, ?→(-).

**Character 123.** Number of lower cusps in transverse row: 1 (0), 2 (1), 3 or more (2). *Aleodon brachyramphus*, 1→(-).

**Character 124.** Lingual cingulum in lower postcanines: absent (0), small (1), well developed (2). *Aleodon brachyramphus*, ?→2. *Probainognathus*, 1→0.

**(b)** Full strict consensus tree of 75 MPTs:

**(2) Additional figures**


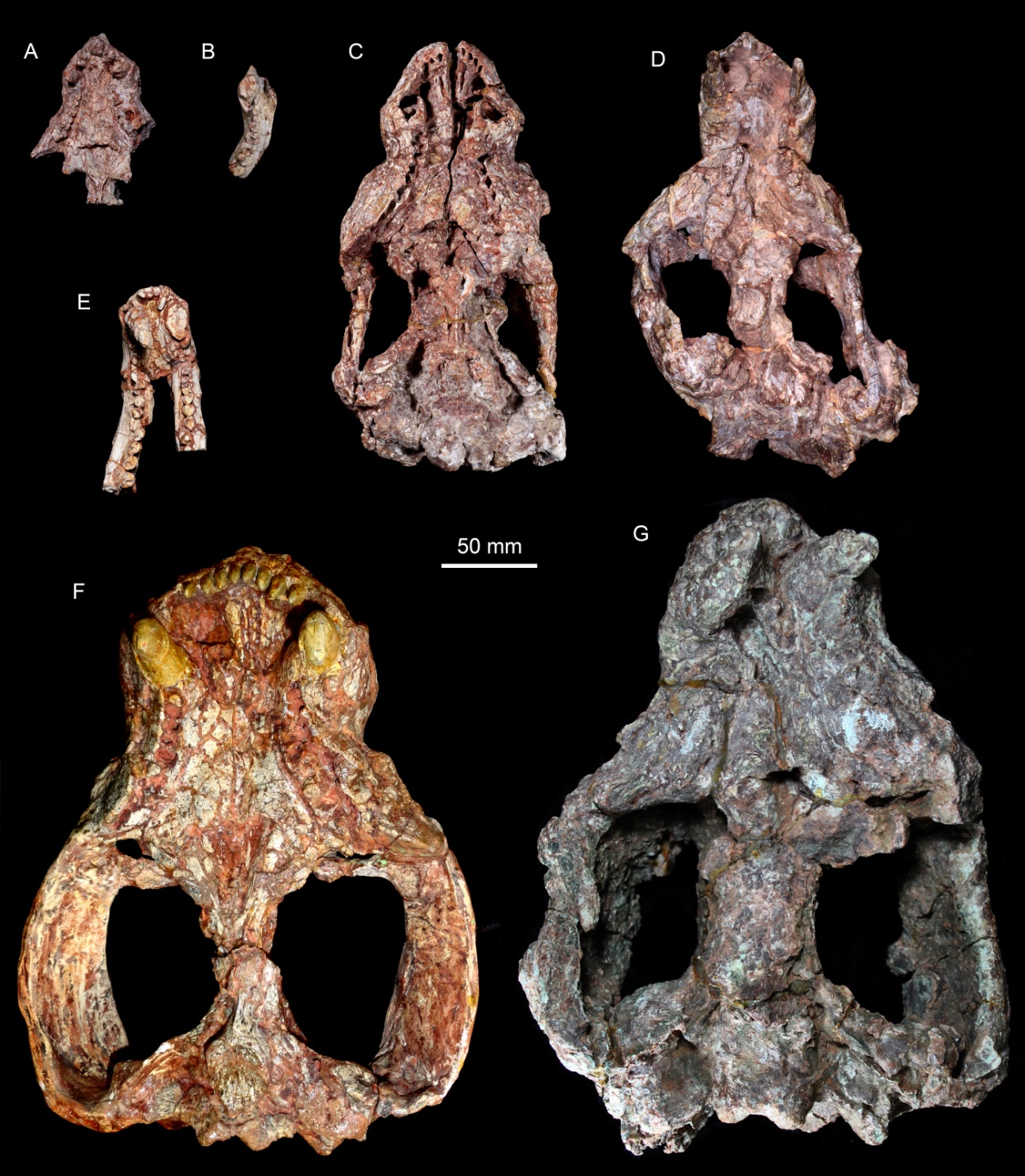


**Fig 1. Selected specimens of *Aleodon cromptoni* sp. nov. from Brazil at the same scale. A**, snout UFRGS-PV-0071-T; **B**, maxilla MPDC-501-117 (holotype); **C**, skull UFRGS-PV-0125-T; **D**, skull UFRGS-PV-0146-T; **E**, lower jaws MCN-PV 10338; **F**, skull UFRGS-PV-0274-T; **G**, skull UFRGS-PV-0122-T. The skull specimens are in ventral view and the jaws in dorsal view.


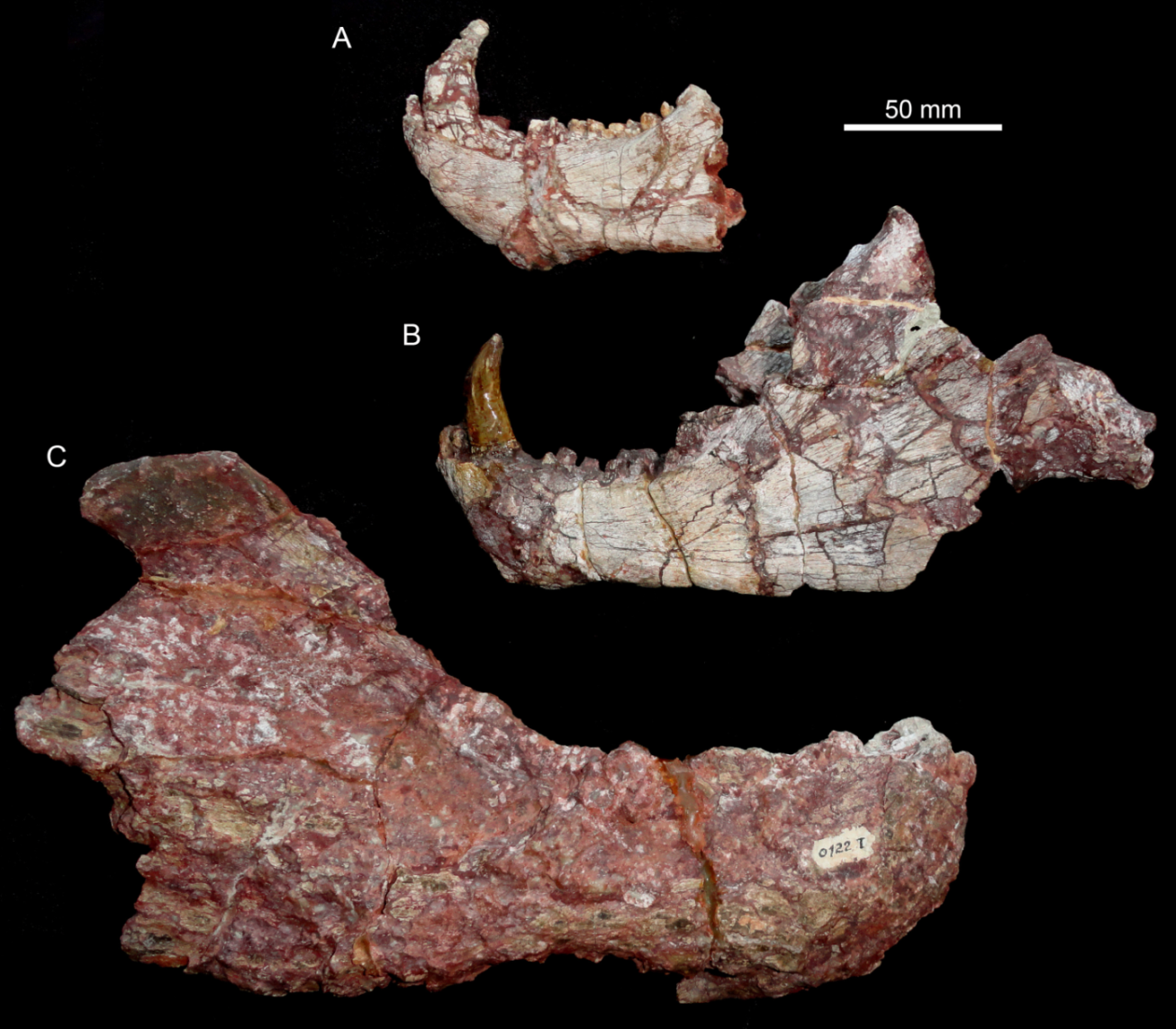


**Fig 2. Selected lower jaws of *Aleodon cromptoni* sp. nov. from Brazil at the same scale, in lateral view.** **A**, MCN-PV 10338; **B**, UFRGS-PV-0146-T; **C**, UFRGS-PV-0122-T.


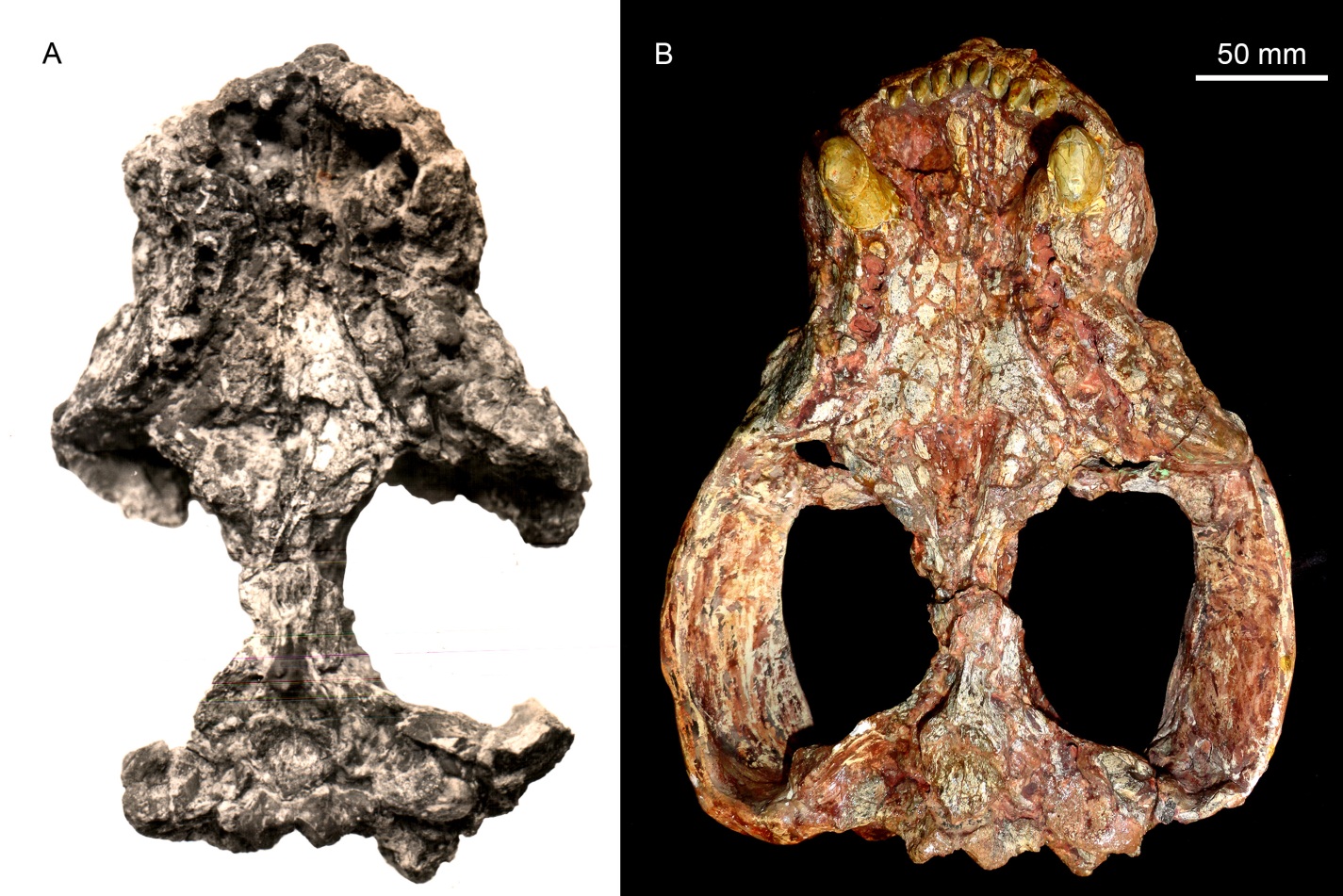


**Fig 3. *Aleodon cromptoni* from Brazil.** Historical photograph of specimen UFRGS-PV-0274-T before (A) and after preparation (B), with reconstruction of zygomatic arches, incisors, and canines.

**
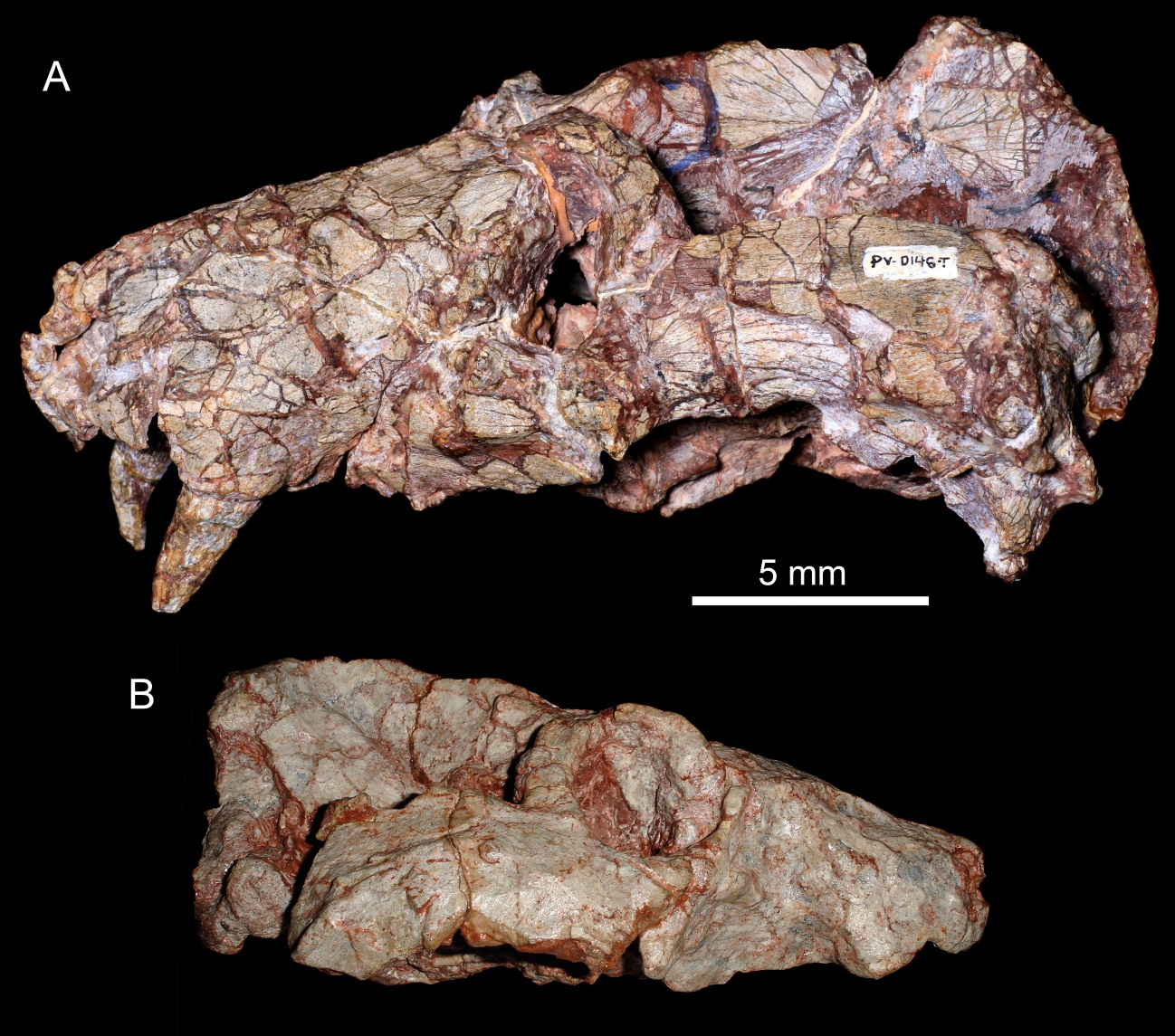
**

**Fig 4. Comparison of *Aleodon* from Brazil and Namibia.** Skull of referred specimens of *A. cromptoni*, UFRGS-PV-0146-T (A) from *Dinodontosaurus* AZ and GSN EN-3 (B) from the upper Omingonde Formation in lateral view.

**(3) Description of UFRGS-PV-0265-T, specimen referred to *Luangwa***

UFRGS-PV-0265-T comes from the same outcrop as the holotype of *Aleodon cromptoni*. It is made up of a set of edentulous lower jaws. The dentaries are preserved with the exception of the dorsal portion of the coronoid process (anteroposterior length: 75 mm from tip to angle of the dentary). The symphysis is fused to the level of the lower canine and the dentary remains low anterior to the coronoid process, which is anteroposteriorly short. The masseteric fossa reaches the level of the penultimate postcanine anteriorly. The posteriorly projected angular process bears a shallow medial fossa that spans the entire length of the process itself and continues anteriorly for approximately the same length, ventral to the postdentary trough, similar to that described for *Luangwa drysdalli* by Kemp (1980). The angular process is posteriorly projected. The lateral profile of the jaws resembles *Luangwa* (Kemp, 1980; Abdala and Sá-Teixeira, 2004) more than that of other known traversodontids (e.g., *Massetognathus*, *Santacruzodon*, *Exaeretodon*; Romer, 1967; Abdala and Ribeiro, 2003; Liparini et al., 2013). The dentition was not preserved, but it is possible to count three procumbent incisor alveoli, one canine (the right canine root is partially preserved), and six postcanines alveoli, fewer than the typical 7-10 for *Luangwa* (Liu and Abdala, 2014). There is no distinct diastema between the canine and postcanine or incisor, as in *L. sudamericana* (Abdala and Sá-Teixeira, 2004). The postcanine alveoli are progressively larger posteriorly in the tooth row, the last alveolus being larger and more quadrangular than the rest. We refer UFRGS-PV-0265-T as *Luangwa* sp., being probably a new specimen of *L. sudamericana*.


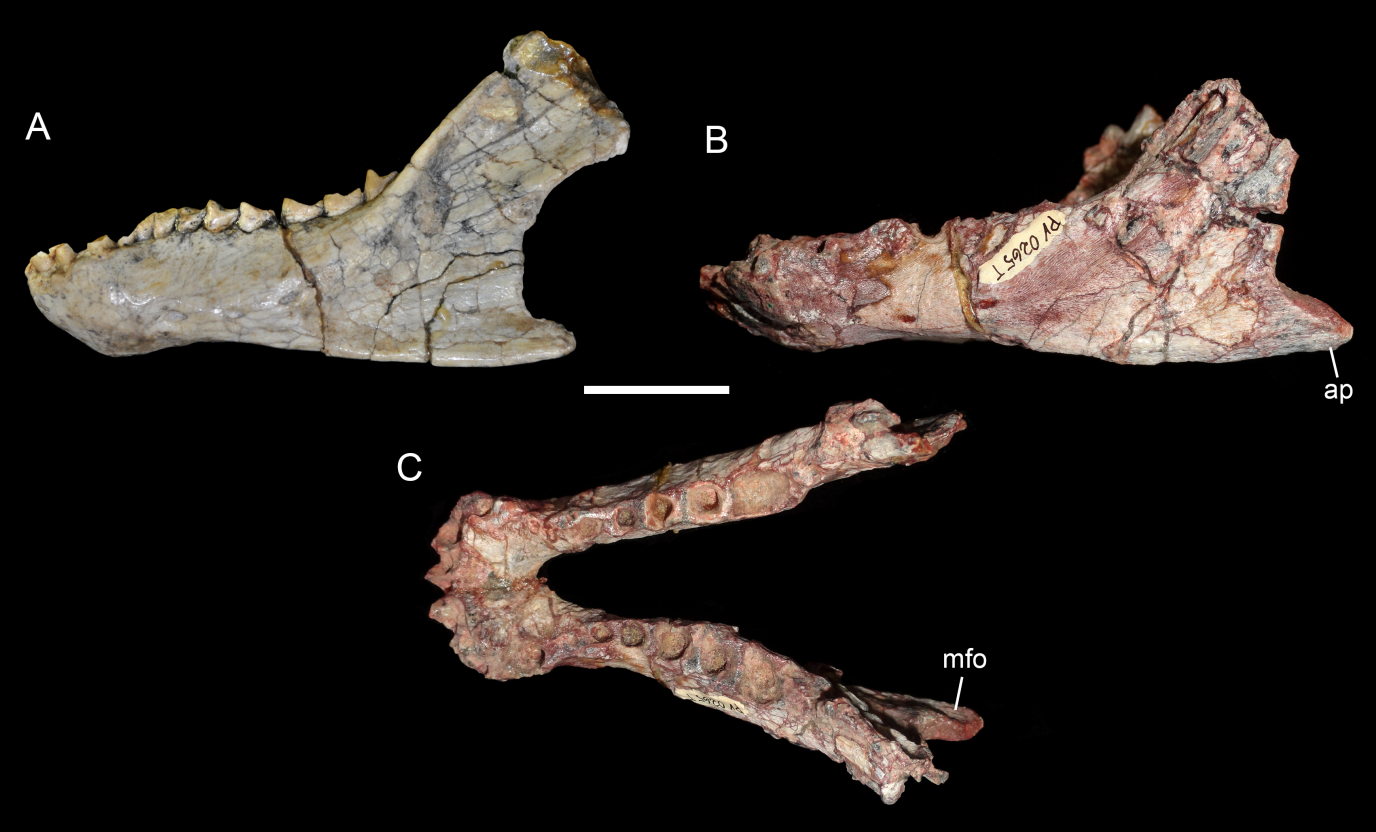


**Fig 5. *Luangwa* from Rio Grande do Sul, Brazil.** Left lower jaw of the holotype MCP-PV-3167 of *Luangwa sudamericana* in lateral view (A) and lower jaws in lateral (B) and dorsal views (C) of specimen UFRGS-PV-0265-T of *Luangwa* sp. from Vale Verde locality. Scale bar equals 20mm. Abbreviations: ap, angular process of dentary; mfo, medial fossa.

**(4) Table 1.** **Main references of the occurrence of taxa into the main localities of the *Dinodontosaurus* AZ in the state of Rio Grande do Sul, southern Brazil.** Abbreviation: Pro., Procolophonia; (#), locality of holotype; (#*), locality of neotype.

|  |  | **Cynodontier Sanga** | **Weg Sanga** | **Baum Sanga** | **Rincão do Pinhal** | **Linha Várzea** | **Dona Francisca (Posto Site and Antonini Bortolin Site)** | **Cortado** | **Pinheiro region** | **Bom Retiro region (Sanga Pascual / Sanga Hintz)** | **Porto Mariante 1** | **Vale Verde** | **Indet. Locality** |
| --- | --- | --- | --- | --- | --- | --- | --- | --- | --- | --- | --- | --- | --- |
|  | **Taxon** | **West region - Chiniquá area** | | | **Central region** | | | | **East region** | | | |  |
| **Dicynodontia** | ***Dinodontosaurus* sp.** | Huene, 1935; Lucas and Harris, 1996 | Huene, 1935; Lucas and Harris, 1996 | Huene, 1935; Lucas and Harris, 1996 | Machado, 1992 | Da-Rosa et al., 2005 | Machado, 1992 | Da-Rosa et al., 2004 | Cox, 1965; Machado, 1992 | Machado, 1992 | Machado, 1992 | Machado, 1992 |  |
|  | ***Stahleckeria potens*** |  |  | (#) Huene, 1935 |  |  |  |  | Romer and Price, 1944; Lucas, 1993 | Peruzzo and Araújo-Barberena, 1995; Lucas, 2002; Vega-Dias et al., 2005 |  |  |  |
| **Cynodontia** | ***Massetognathus* (*M. ochagaviae*, *M. pascuali,* *M.* sp.)** |  |  |  | (#*) Liu et al., 2008 | Da-Rosa et al., 2005 (AGM Pers. obs. of UFSM 11230, 11534) | Pavanatto et al., 2016; MCN-PV 2293 | Da-Rosa et al., 2004 (AGM Pers. obs. UFSM11096, 11162) | Barberena, 1974; Teixeira, 1995; Bertoni-Machado et al., 2008 | Teixeira, 1987 |  | (#) Barberena, 1981b |  |
|  | ***Traversodon stahleckeri* / *?T. major*** | (#) Huene, 1936; Barberena, 1981a |  | Huene, 1936; Liu and Abdala, 2014 |  |  |  |  |  |  |  |  |  |
|  | ***Protuberum cabralense*** |  |  |  | Reichel et al., 2009 |  |  | (#) Reichel et al., 2009 |  |  |  |  |  |
|  | ***Luangwa sudamericana* / *L. sp.*** |  |  |  |  |  | Da Silva and Cabreira, 2009; Hanich et al., 2013; MCN-PV 3599 |  |  | UFRGS-PV-0140-T (AGM and TPM Pers. obs.) |  | UFRGS-PV-0265-T (This study) | Abdala and Sá-Teixeira, 2004 |
|  | ***Chiniquodon theotonicus*** | (#) von Huene, 1936; Abdala and Giannini, 2002 | Huene, 1936 | Huene, 1936 | Teixeira, 1979, 1982 (holotipo de *P. kirchingi*) | Da-Rosa et al., 2005 (AGM Pers. obs. UFSM 11428, 11244) |  |  | Abdala and Giannini, 2002 |  |  |  |  |
|  | ***Aleodon cromptoni*** |  |  |  |  |  |  | MCN-PV 10338 (This study) | UFRGS-PV-0071-T, UFRGS-PV-0122-T, UFRGS-PV-0125-T, MMACR-PV-018-T (This study) | UFRGS-PV-0146-T (This study) |  | MPDC-501-117 (This study) |  |
|  | ***Bonacynodon schultzi*** |  |  |  |  |  |  |  | Martinelli et al., 2016 |  |  |  |  |
|  | ***Candelariodon barberenai*** |  |  |  |  |  |  | Da-Rosa et al., 2004 (AGM Pers. Obs. UFSM11079) | Oliveira et al., 2011 |  |  |  |  |
|  | ***Protheriodon estudianti*** |  |  |  |  |  | Bonaparte et al., 2006 |  |  |  |  |  |  |
| **Pro.** | ***Candelaria barbouri*** |  |  |  |  |  |  | Da-Rosa et al., 2004 | Price, 1946 |  |  |  |  |
| **Archosauromorpha** | ***Brasinorhynchus mariantensis*** |  |  |  |  |  |  |  |  |  | (#) Schultz et al., 2016 |  |  |
|  | **Proterochampsidae indet.** |  |  |  |  |  |  |  | Dornelles, 1992, 1995 |  |  |  |  |
|  | ***Barberenasuchus brasiliensis*** |  |  |  |  |  |  | (#) Mattar, 1987; Langer et al., 2007 |  |  |  |  |  |
|  | ***Archeopelta arborensis*** |  |  | (#) Desojo et al., 2011 |  |  |  |  |  |  |  |  |  |
|  | ***Decuriasuchus quartacolonia*** |  |  |  |  |  | (#) França et al., 2011 |  |  |  |  |  |  |
|  | ***Prestosuchus chiniquensis*** | Huene, 1942 | (#) Huene, 1942 | Lacerda et al., 2016 |  | Unpublished specimens at UFSM Collection (Pers. obs. AASD) | Mastrantonio et al., 2013 |  | UFRGS-PV-0022-T, UFRGS-PV-0065-T (VDPN and AGM Pers. obs.) | Barberena, 1978 |  | Nesbitt, 2011; Raugust, 2014 |  |
|  | ***Spondylosoma absconditum*** | Huene, 1942 |  | (#) Huene, 1942; Langer, 2004 |  |  |  |  |  |  |  |  |  |
|  | **Number of taxa/locality** | 5 | 3 | 7 | 4 | 4 | 6 | 7 | 10 | 6 | 2 | 5 |  |

**(5) Comments on fossiliferous localities from the *Dinodontosaurus* AZ:**

1. See meaning of the word **Sangas** in Beltrão (1965), Langer et al., (2007), and Martinelli et al., (2016).
2. The **Chiniquá region** (=Xiniquá, =Rincão dos Colorados, =Colorados, =“Barrocas”) is located in the municipality of São Pedro do Sul (Fig 6). The Chiniquá outcrops were intensely worked since the beginning of 20^th^ century (see references in the Table 1, Zingano and Cauduro, 1959, and Beltrão, 1965) (Fig 7). The most fossiliferous sangas at that region are: **Sanga Cynodontier** (=Cynodont Sanga, =Sanga dos Cinodontes, =Sanga Béles, =Sanga de Theotônio Béles Xavier, =Sanga north to the house of Theotônio Béles Xavier), **Sanga Baum** (=Sanga da Árvore), **Sanga Weg** (=Sanga do Caminho, =Sanga da Estrada). This region has several other outcrops that were denominated: Rote Sanga (=Sanga Vermelha), Sanga Leere (=Sanga Vazia), Sanga Cesar (=Sanga César), and Sanga Suld-Weg (=Sanga south to Sanga Weg, =Sanga ao sul da Sanga Weg). The location of some of these outcrops is likely imprecise since the topology of the region changes drastically due to anthropic activities (see Beltrão, 1965; Dassie, 2014; Lacerda et al., 2016).


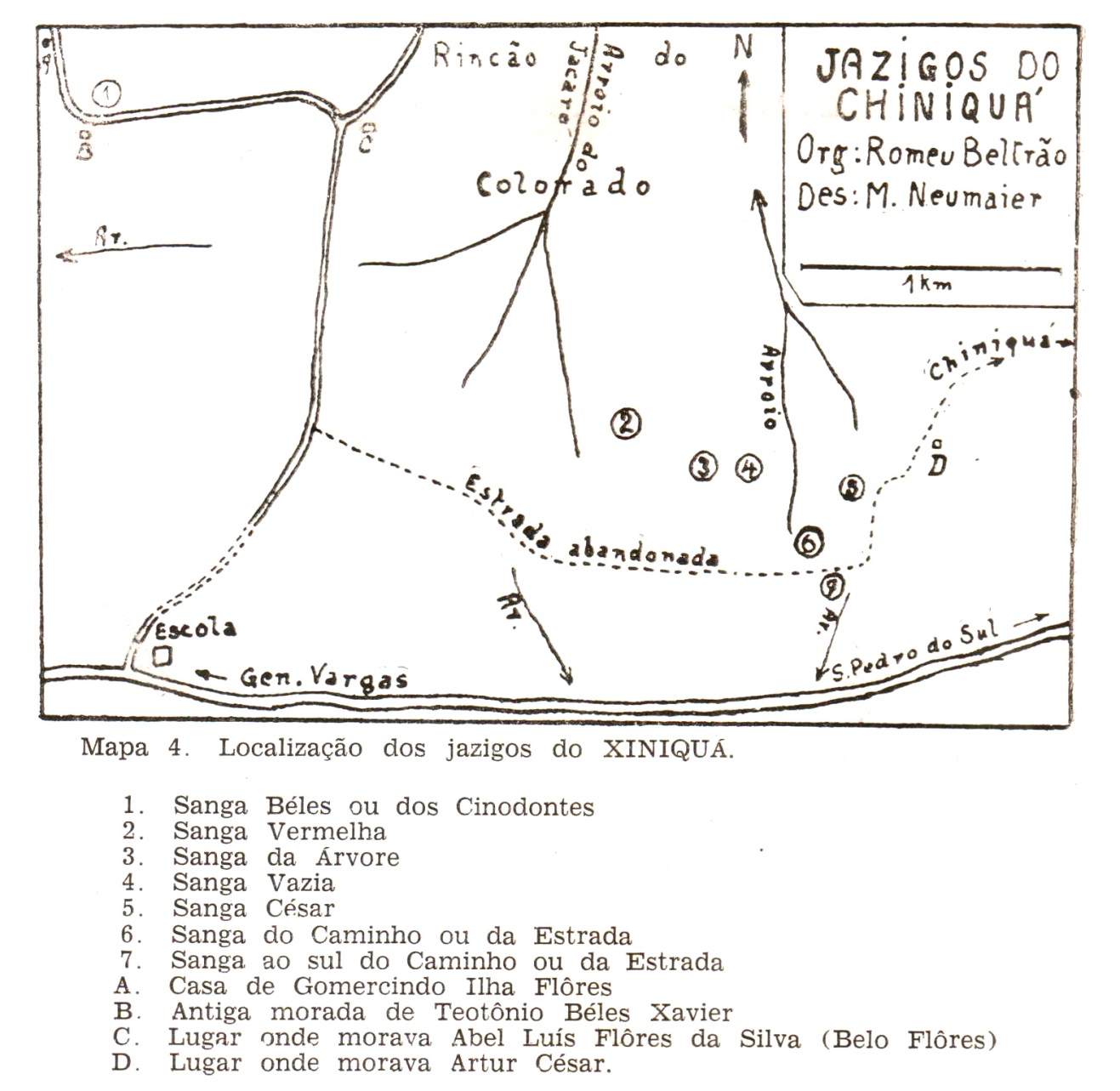


**Fig 6.** Map taken from Beltrão (1965:pag. 88) showing the sangas from which most specimens published by von Huene (1935, 1936, 1942) came from, in the Chiniquá region, municipality of São Pedro do Sul.


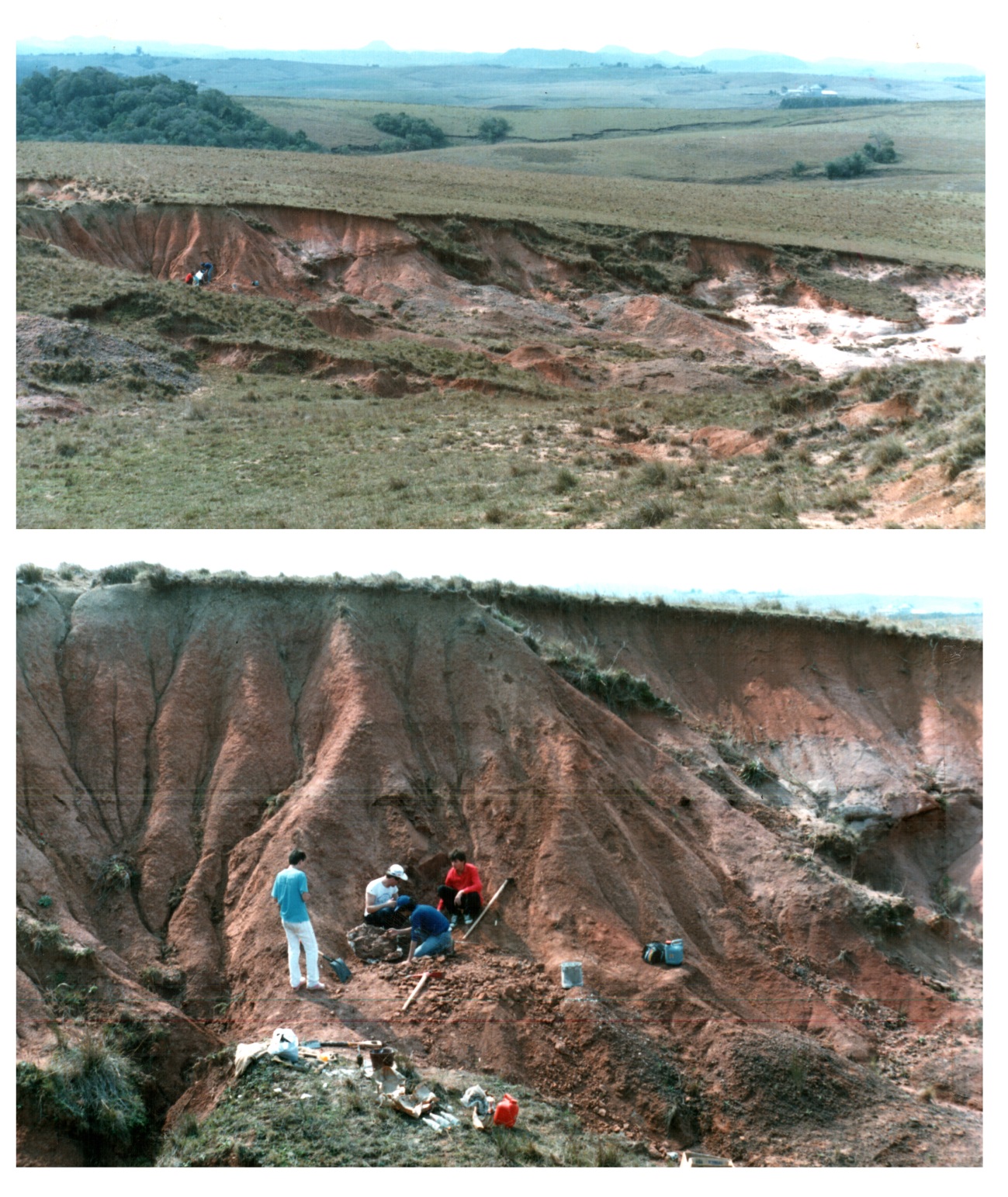


**Fig 7.** General views of outcrops in Chiniquá region in 2000.

1. The **Dona Francisca** region refers to **Posto site** (Figs 8-9) and **Antonini Bortolin site**. The Posto site comprises a large outcrop located behind and lateral to the Gas Station at the entrance of Dona Francisca city (Franca et al., 2011; Mastrantonio et al., 2013; Pavanatto et al., 2016). At present, all specimens collected at Bortolin site remains formally unpublished and were only mentioned in abstract meetings (Silva and Cabreira, 2009; Hanich et al., 2012, 2013).


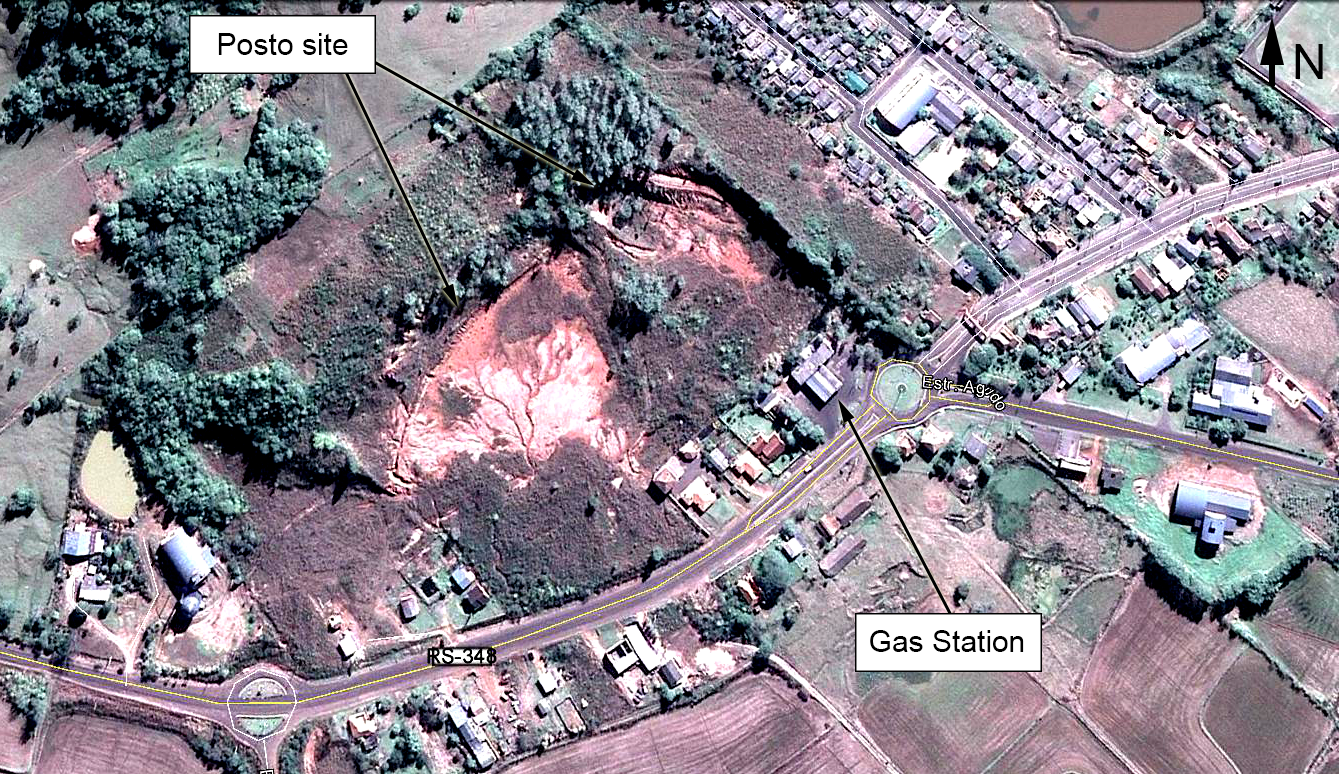


**Fig 8.** Posto site with Dona Francisca town at the right top (taken from Google Earth, photograph 2009).


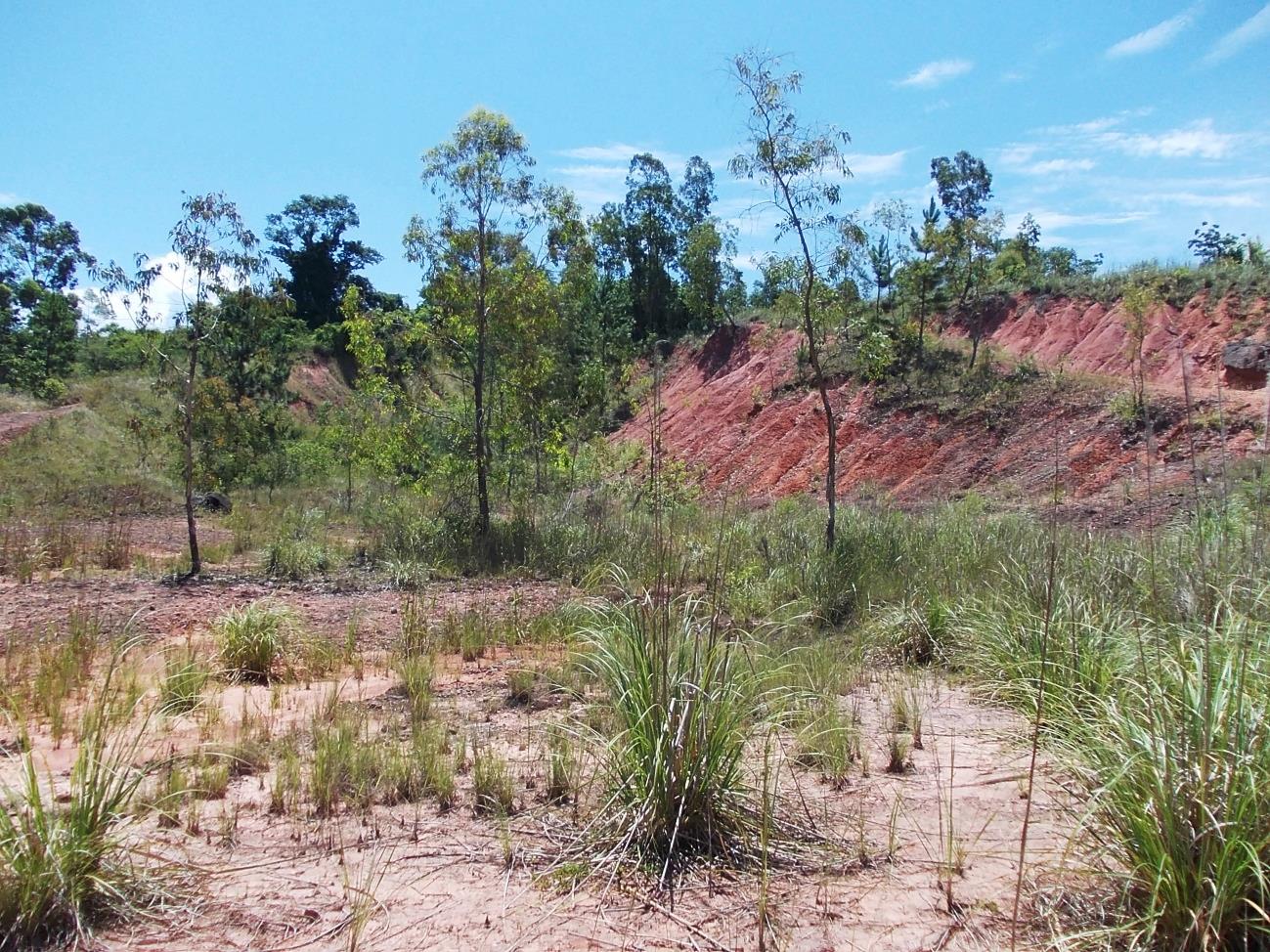


**Fig 9.** West portion of Posto site in Dona Francisca, in 2013.

1. The **Linha Várzea site** (Fig 10) includes two nearby outcrops at the borders of the rural road that connect the Highway RST-287 (km 188) to Vila Paraíso do Sul, in the municipality of Paraíso do Sul (Da-Rosa et al., 2005).


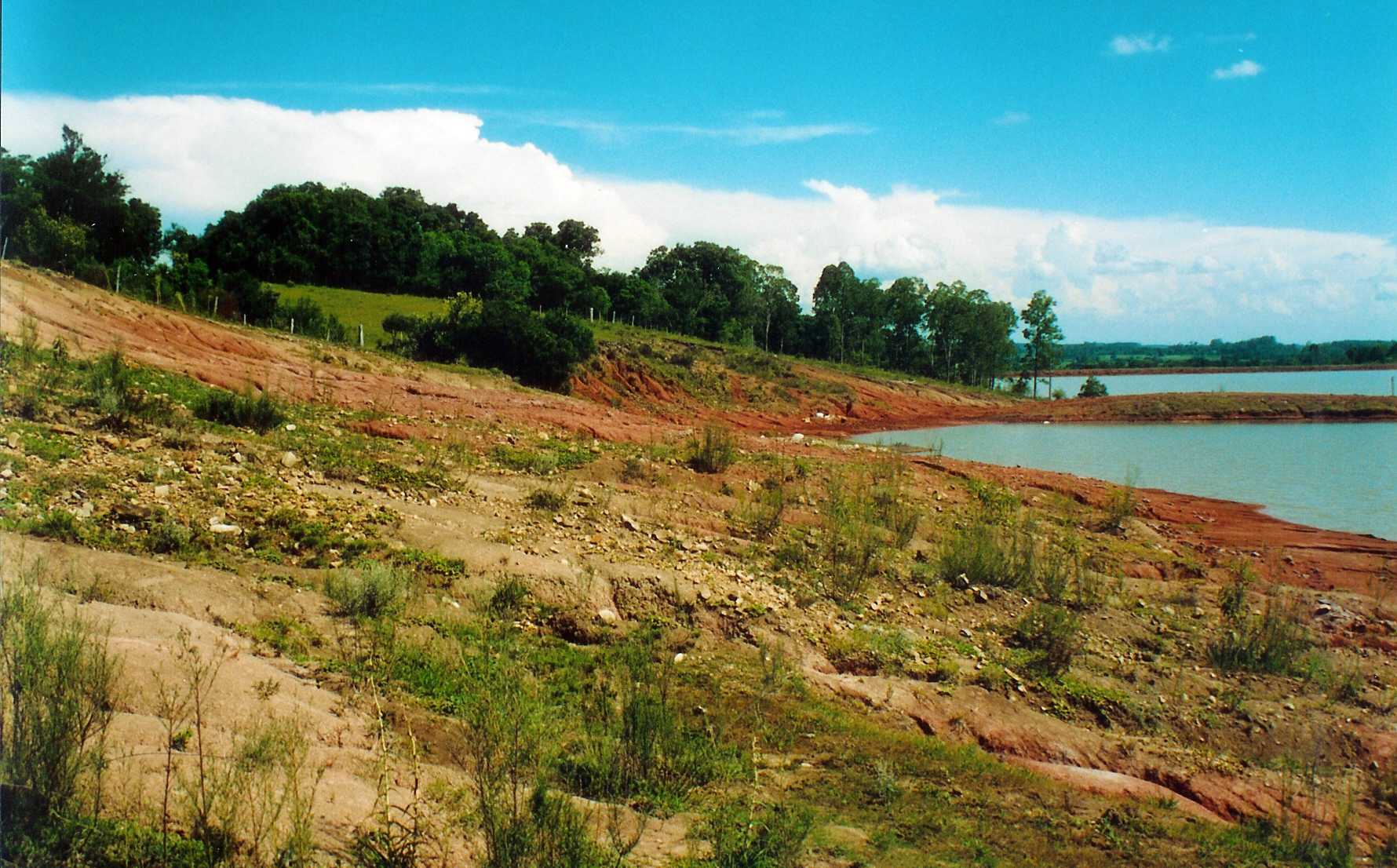


**Fig 10.** Photograph of Linha Várzea outcrop in 2002.

1. The **Rincão do Pinhal** **site** (Fig 11) is located next to the Highway BR-287, west to the Paraíso do Sul city.


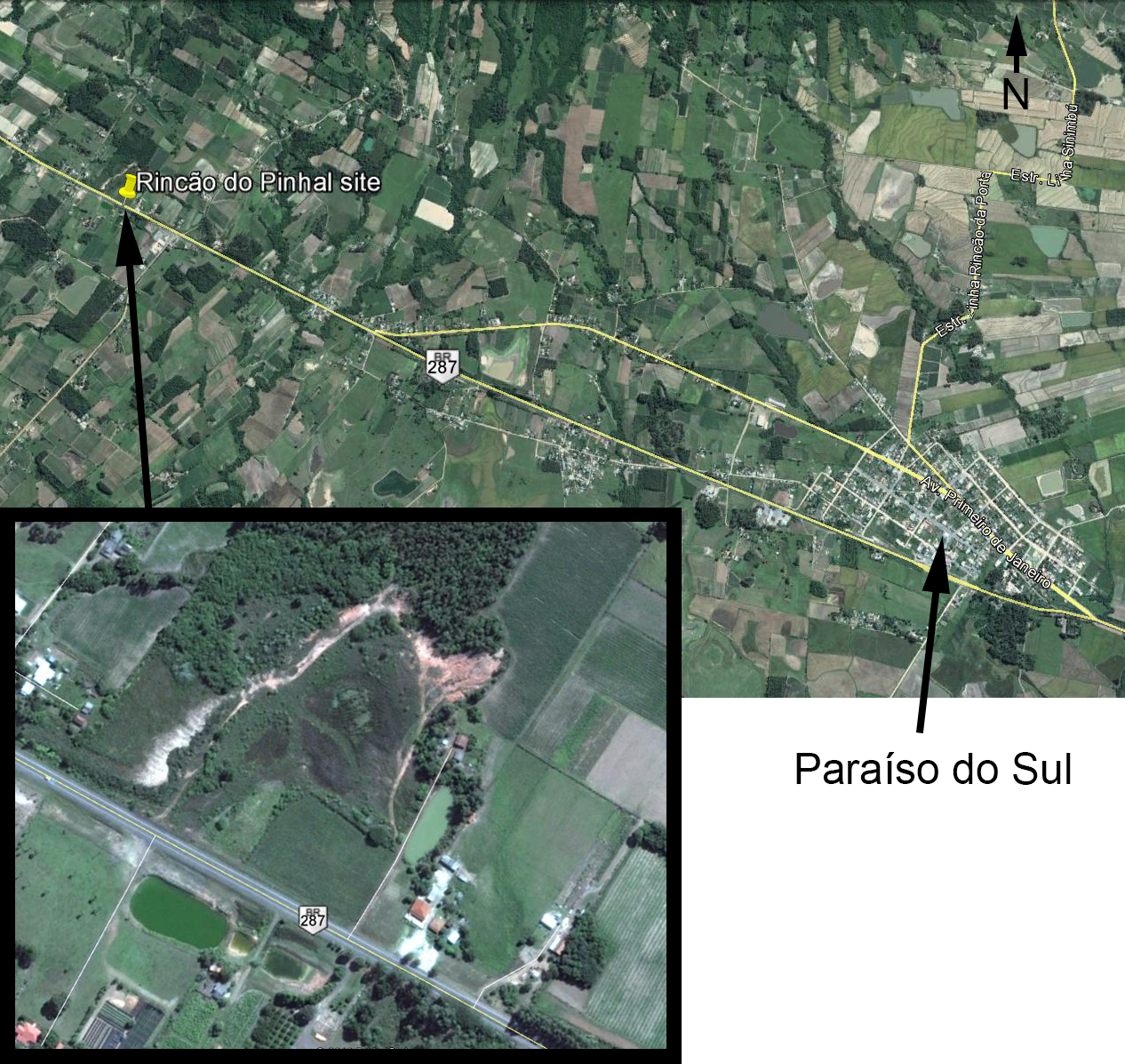


**Fig 11.** Rincão do Pinhal site in the municipality of Paraíso do Sul (taken from Google Map, photograph of 2009).

1. The **Cortado site** (Fig 12) includes at least two outcrops at the borders of the Highway RST-287 (Da-Rosa et al., 2004), municipality of Novo Cabrais. It is also known as **Rincão da Porta** in some catalog books. Langer et al. (2007) suggested that Rincão da Porta is a nearby site of Cortado Site. Accordingly to historical data, both sites refer to the same outcrop, and both outcrops are located in between Rincão da Porta and Cortado towns.


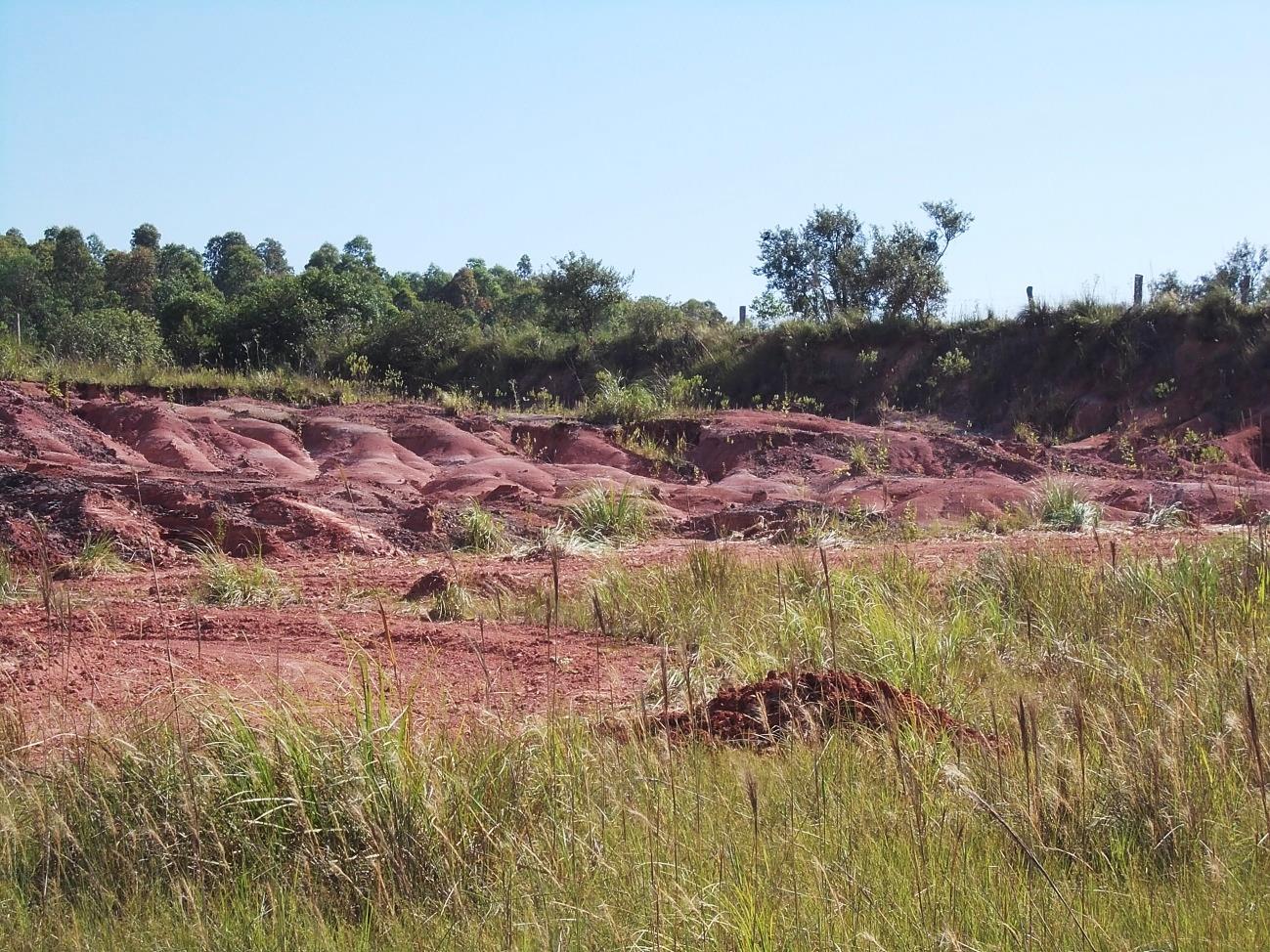


**Fig 12.** Cortado site, outcrop at the north border of the road, in 2013.

1. The **Pinheiro region** (=Pinheiros, =Vila Pinheiros) refers to the sangas cropping out near the Pinheiro town, about 12km south to the city of Candelária, in the municipality of Candelária (Figs 13-14). Due to the proximity with Bom Retiro town (see below), old contributions considered both areas (i.e., Pinheiro and Bom Retiro) as being part of the Pinheiros Fauna (see Barberena, 1977; Barberena et al., 1985; Schultz et al., 2000). The most fossiliferous sangas in the Pinheiro region are: Sanga Pinheiro [=Sanga Pinheiros, =Sanga dos Fósseis (Fossil´s Sanga), =Sanga Carlos, and possibly =Sanga do Forno (“Furnace´s Sanga”)] (Fig 13), Sanga Belmiro (=Sanga Lili), Sanga Nicanor (=Sanga do Zé, Rincão do Simeão), Sanga da Divisa, Sanga Janguta, Sanga do Ribeiro, among others. The location of some of these outcrops is likely imprecise since the topology of the region changes drastically due to anthropic activities (see Beltrão, 1965; Langer et al., 2007; Martinelli et al., 2016) and correspond to local, non-formal names. Mentions of these Sangas/localities appear, for example, in catalog labels/books of several specimens/museums (also in the references in Table 1 and references cited in main text).


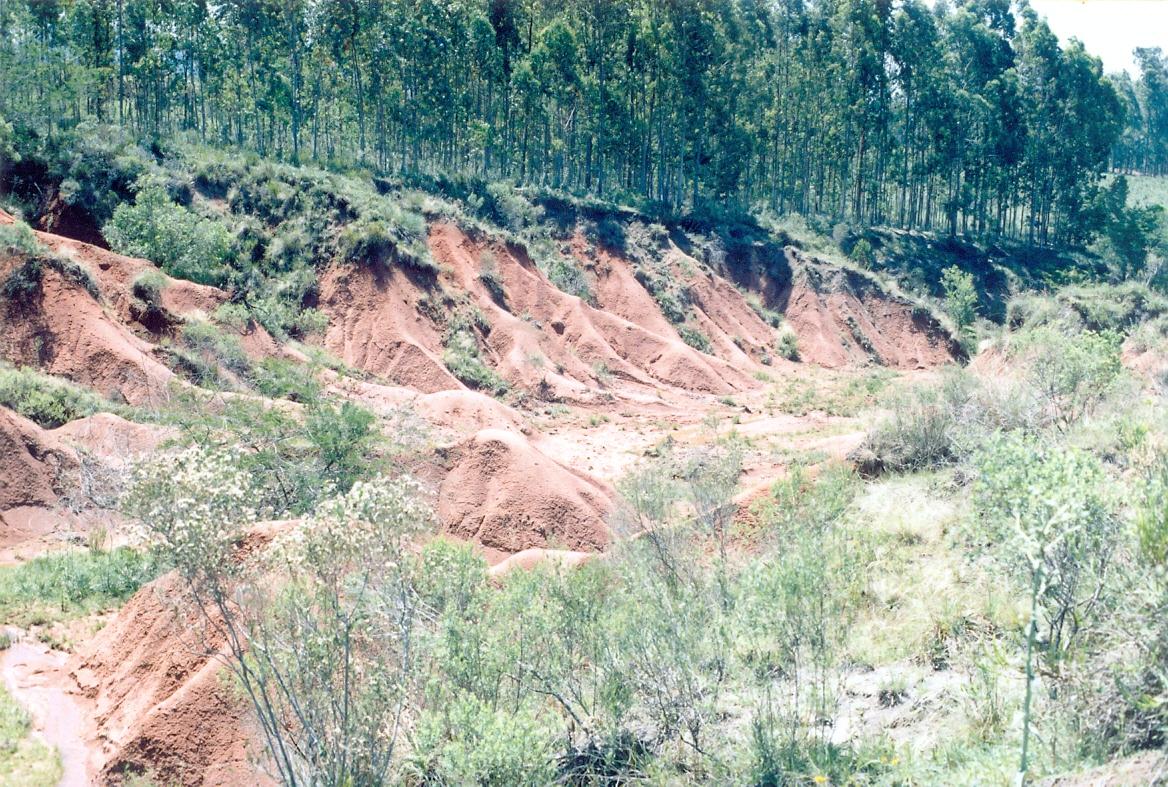


**Fig 13.** Photograph of Sanga Pinheiro outcrop in 1999.


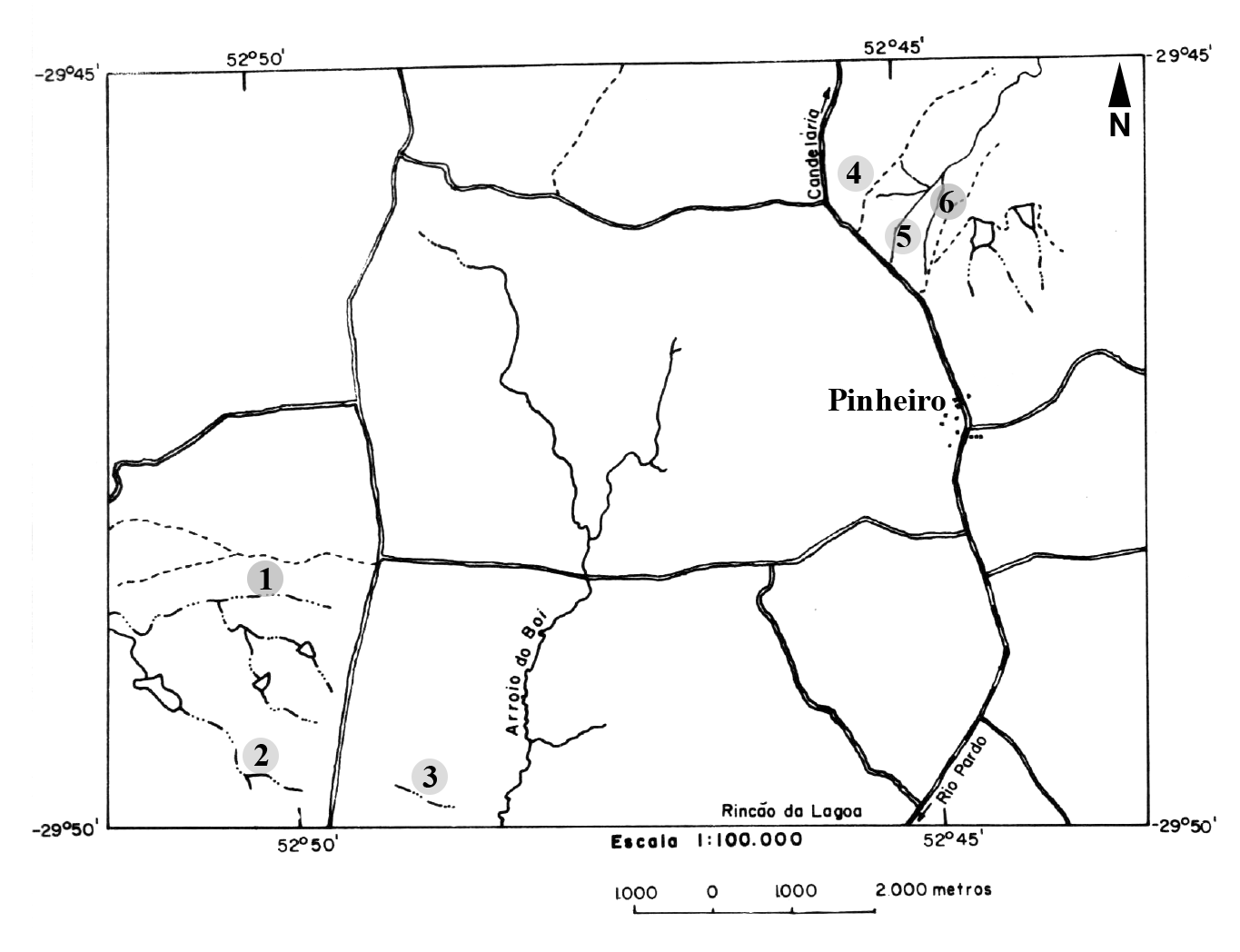


**Fig 14.** Map with sangas according to Barberena (1977:115, modified), in the municipality of Candelária. 1= Sanga Hintz, 2= Sanga Pascual, 3= Sanga do Ribeiro, 4= Sanga 1 and Sanga 2, 5= Sanga da Divisa, 6= Sanga Pinheiro.

1. The Bom Retiro town is located about 10km southwest of the Pinheiro town, in the municipality of Candelária. The main sangas at the **Bom Retiro region** are: **Sanga Hintz** and **Sanga Pascual**. They were referred as Sanga I and Sanga II, respectively, by Price but Barberena (1977) proposed the mentioned names after the surname of the owners of the properties where the outcrops are located. Other fossiliferous sites from this region are: Sanga Menezes (Fig 15) and Cerro dos Gomes.


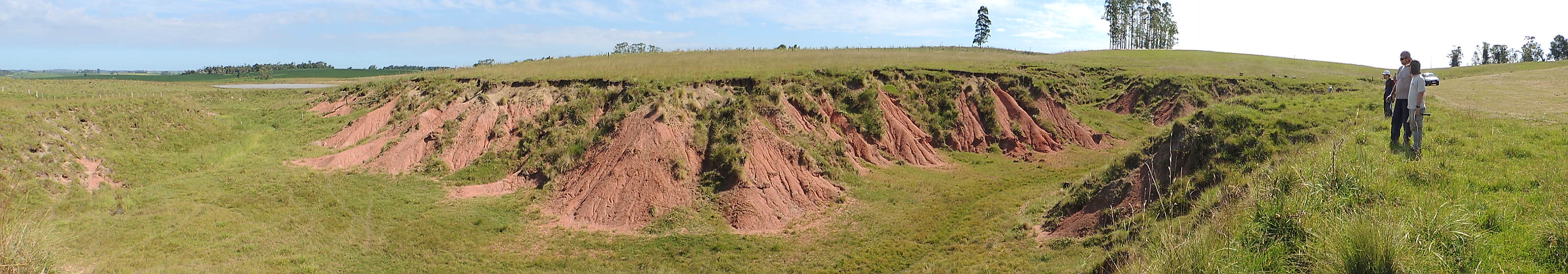


**Fig 15.** Outcrops in Sanga Menezes in the Bom Retiro region, municipality of Candelária. Taken in 2014.

1. Other places in the municipalities of Candelária, Rio Pardo and Cachoeira do Sul with mention of fossils of the *Dinodontosaurus* AZ in catalog books are: Rincão dos Bois (located south to Pinheiro town), Campo (or Sanga) do Caranguejo (located south of Pinheiro town), Sanga Erotilde, Oveiras, Capão do Valo, among others.
2. The **Porto Mariante 1 site** (Fig 16) is located on the north side of the Highway RS-240 near the town of Porto Mariante, about 28km east of Venâncio Aires city, in the municipality of Bom Retiro do Sul (it is different from the Bom Retiro town in Candelária). Nearby this site, there is another site known as Porto Mariante 2, which has yielded several specimens of *Dinodontosaurus* (see Schultz et al., 2016) and a record of *Aleodon* (specimen MCP-PV-1695T).


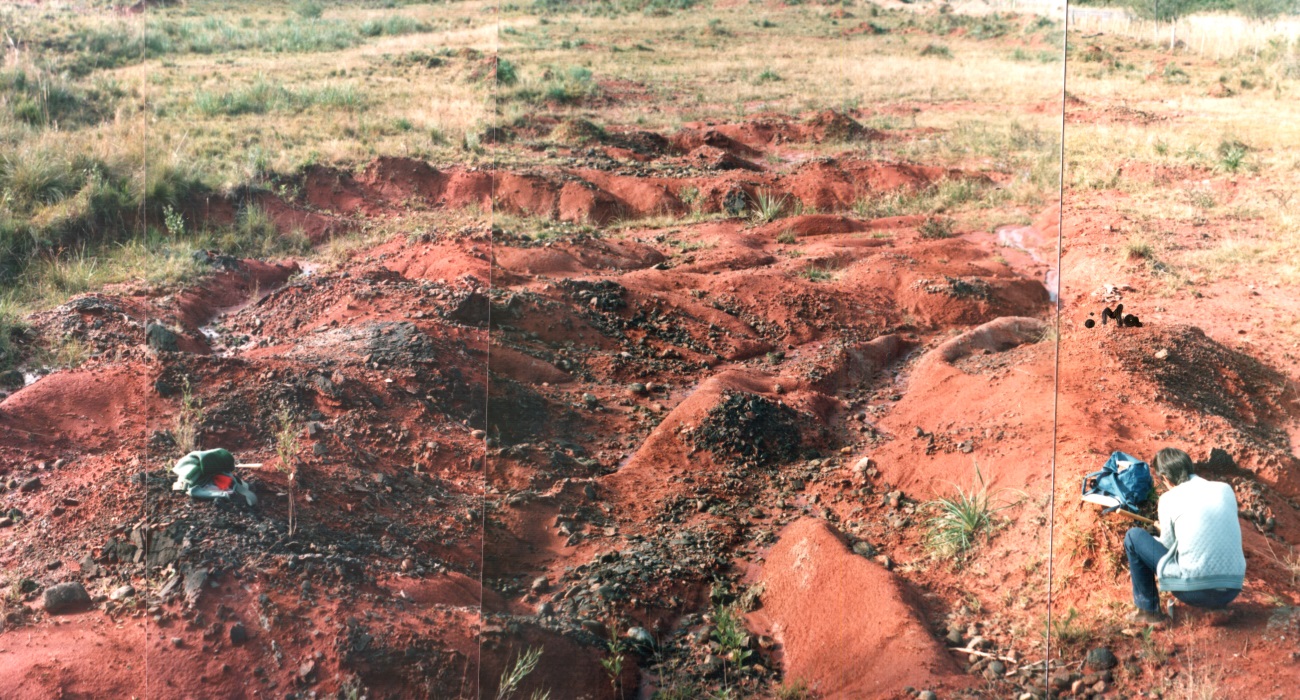


**Fig 16.** Photograph of Porto Mariante 1 site. Photograph taken in 1998.

1. The **Vale Verde site** (Figs 17-18) is here referred to outcrops on and nearby the railroad that cross the Cria Farm (“Fazenda Cria”) in the municipality of Vale Verde. This site was also mentioned in the literature or catalog book as: (a) outcrop located a few kilometers from the train station Prof. Parreira, in Vila Melos (=Vila Melo); (b) or simply General Câmara locality (see main text). The presence of other fossiliferous outcrops in the region is likely probable, being also cataloged as “General Câmara”; therefore, some historical findings can be difficult to be located properly.


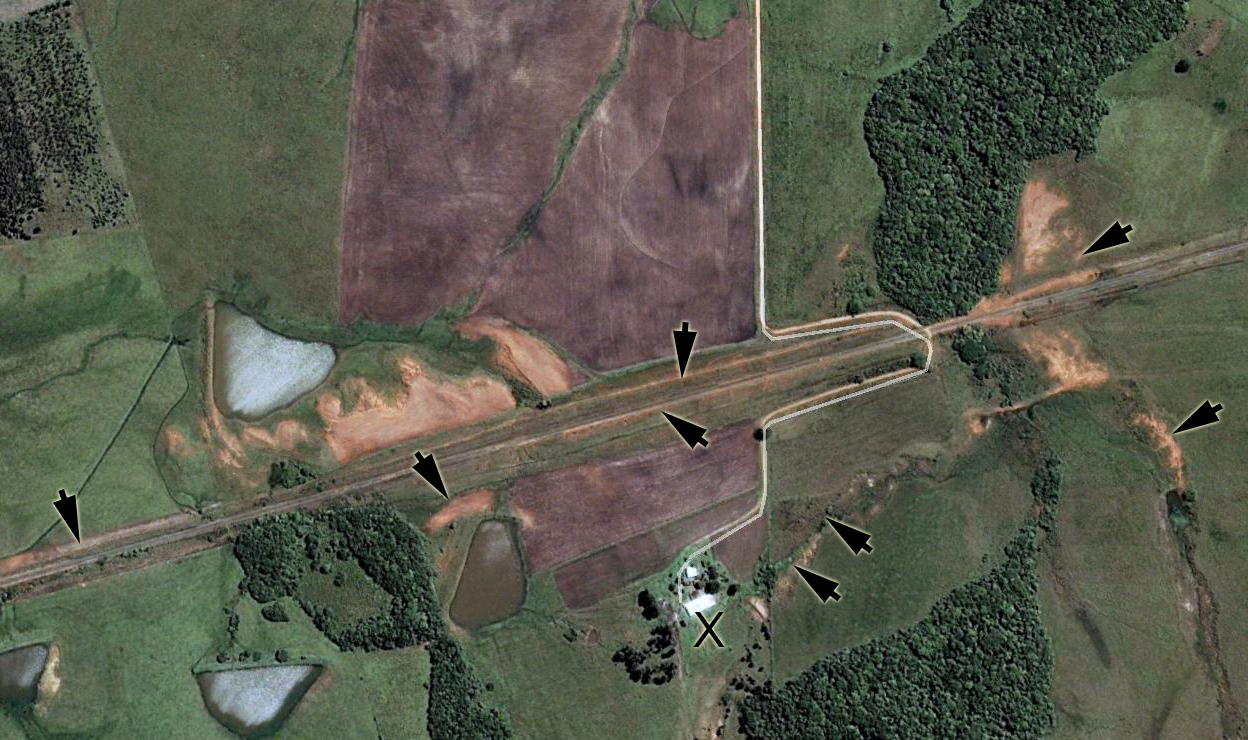


**Fig 17.** The arrows indicates the main outcrops in Vale Verde site in the Cria Farm (the “X” indicates the house of the farm). Taken from Google Earth (photograph of 2002).


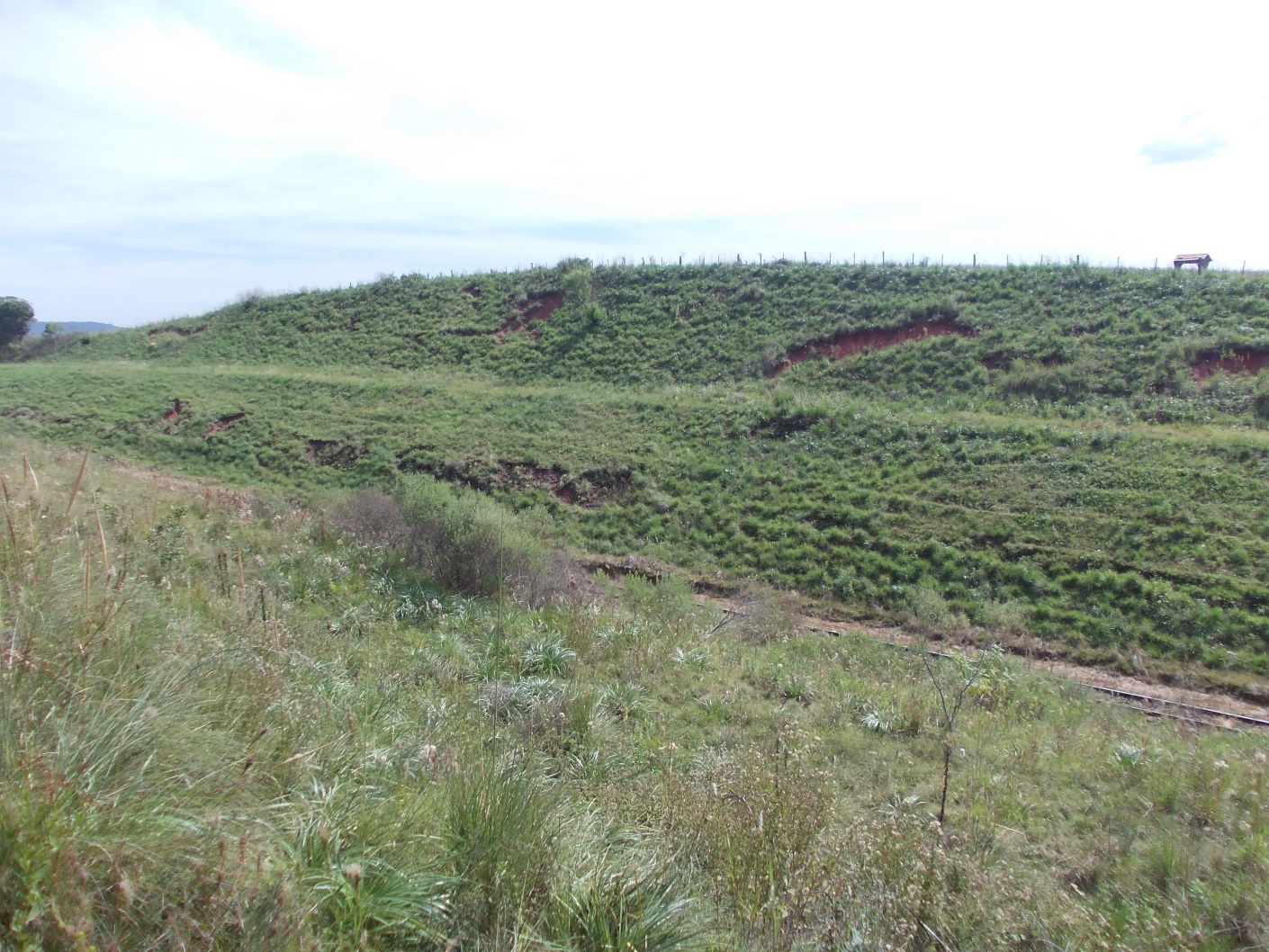


**Fig 18.** Vale Verde outcrop, next to railroad, mostly covered by vegetation. Photograph taken in 2016.

**(6) Table 2. Matrix for cluster analysis including taxa and main localities of the *Dinodontosaurus* AZ of southern Brazil.** The codifications refer to 0=absence and 1=presence of a taxon into a locality.

|  | Cynodontier Sanga | Weg Sanga | Baum Sanga | Rincão do Pinhal | Linha Várzea | Dona Francisca (Posto Site - Antonini Bortolin Site) | Cortado | Pinheiro region | Bom Retiro region (Sanga Pascual / Sanga Hintz) | Porto Mariante 1 | Vale Verde |
| --- | --- | --- | --- | --- | --- | --- | --- | --- | --- | --- | --- |
| *Dinodontosaurus* sp. | 1 | 1 | 1 | 1 | 1 | 1 | 1 | 1 | 1 | 1 | 1 |
| *Stahleckeria potens* | 0 | 0 | 1 | 0 | 0 | 0 | 0 | 1 | 1 | 0 | 0 |
| *Massetognathus* (*M. ochagaviae*, *M. pascuali,* *M.* sp.) | 0 | 0 | 0 | 1 | 1 | 1 | 1 | 1 | 1 | 0 | 1 |
| *Traversodon stahleckeri* / *?T. major* | 1 | 0 | 1 | 0 | 0 | 0 | 0 | 0 | 0 | 0 | 0 |
| *Protuberum cabralense* | 0 | 0 | 0 | 1 | 0 | 0 | 1 | 0 | 0 | 0 | 0 |
| *Luangwa sudamericana* / *L.* sp. | 0 | 0 | 0 | 0 | 0 | 1 | 0 | 0 | 1 | 0 | 1 |
| *Chiniquodon theotonicus* | 1 | 1 | 1 | 1 | 1 | 0 | 0 | 1 | 0 | 0 | 0 |
| *Aleodon cromptoni* | 0 | 0 | 0 | 0 | 0 | 0 | 1 | 1 | 1 | 0 | 1 |
| *Bonacynodon schultzi* | 0 | 0 | 0 | 0 | 0 | 0 | 0 | 1 | 0 | 0 | 0 |
| *Candelariodon barberenai* | 0 | 0 | 0 | 0 | 0 | 0 | 1 | 1 | 0 | 0 | 0 |
| *Protheriodon estudianti* | 0 | 0 | 0 | 0 | 0 | 1 | 0 | 0 | 0 | 0 | 0 |
| *Candelaria barbouri* | 0 | 0 | 0 | 0 | 0 | 0 | 1 | 1 | 0 | 0 | 0 |
| *Brasinorhynchus mariantensis* | 0 | 0 | 0 | 0 | 0 | 0 | 0 | 0 | 0 | 1 | 0 |
| Proterochampsidae indet. | 0 | 0 | 0 | 0 | 0 | 0 | 0 | 1 | 0 | 0 | 0 |
| *Barberenasuchus brasiliensis* | 0 | 0 | 0 | 0 | 0 | 0 | 1 | 0 | 0 | 0 | 0 |
| *Archeopelta arborensis* | 0 | 0 | 1 | 0 | 0 | 0 | 0 | 0 | 0 | 0 | 0 |
| *Decuriasuchus quartacolonia* | 0 | 0 | 0 | 0 | 0 | 1 | 0 | 0 | 0 | 0 | 0 |
| *Prestosuchus chiniquensis* | 1 | 1 | 1 | 0 | 1 | 1 | 0 | 1 | 1 | 0 | 1 |
| *Spondylosoma absconditum* | 1 | 0 | 1 | 0 | 0 | 0 | 0 | 0 | 0 | 0 | 0 |

**Table 3. Jaccard similarities indices.**

|  | **Cynodontier Sanga** | **Weg Sanga** | **Baum Sanga** | **Rincão do Pinhal** | **Linha Várzea** | **Dona Francisca** | **Cortado** | **Pinheiro region** | **Bom Retiro region** | **Porto Mariante 1** | **Vale Verde** |
| --- | --- | --- | --- | --- | --- | --- | --- | --- | --- | --- | --- |
| **Cynodontier Sanga** | 1 | 0,6 | 0,71429 | 0,28571 | 0,5 | 0,22222 | 0,090909 | 0,25 | 0,22222 | 0,16667 | 0,25 |
| **Weg Sanga** | 0,6 | 1 | 0,42857 | 0,4 | 0,75 | 0,28571 | 0,11111 | 0,3 | 0,28571 | 0,25 | 0,33333 |
| **Baum Sanga** | 0,71429 | 0,42857 | 1 | 0,22222 | 0,375 | 0,18182 | 0,076923 | 0,30769 | 0,3 | 0,125 | 0,2 |
| **Rincão do Pinhal** | 0,28571 | 0,4 | 0,22222 | 1 | 0,6 | 0,25 | 0,375 | 0,27273 | 0,25 | 0,2 | 0,28571 |
| **Linha Várzea** | 0,5 | 0,75 | 0,375 | 0,6 | 1 | 0,42857 | 0,22222 | 0,4 | 0,42857 | 0,2 | 0,5 |
| **Dona Francisca** | 0,22222 | 0,28571 | 0,18182 | 0,25 | 0,42857 | 1 | 0,18182 | 0,23077 | 0,5 | 0,14286 | 0,57143 |
| **Cortado** | 0,090909 | 0,11111 | 0,076923 | 0,375 | 0,22222 | 0,18182 | 1 | 0,41667 | 0,3 | 0,125 | 0,33333 |
| **Pinheiro region** | 0,25 | 0,3 | 0,30769 | 0,27273 | 0,4 | 0,23077 | 0,41667 | 1 | 0,45455 | 0,090909 | 0,36364 |
| **Bom Retiro region** | 0,22222 | 0,28571 | 0,3 | 0,25 | 0,42857 | 0,5 | 0,3 | 0,45455 | 1 | 0,14286 | 0,83333 |
| **Porto Mariante 1** | 0,16667 | 0,25 | 0,125 | 0,2 | 0,2 | 0,14286 | 0,125 | 0,090909 | 0,14286 | 1 | 0,16667 |
| **Vale Verde** | 0,25 | 0,33333 | 0,2 | 0,28571 | 0,5 | 0,57143 | 0,33333 | 0,36364 | 0,83333 | 0,16667 | 1 |

**Bibliography**

Abdala, F., Giannini, N.P., 2002. Chiniquodontid cynodonts: systematic and morphometric considerations. Palaeontology 45, 1151–1170. DOI: 10.1111/1475-4983.00280

Abdala, F., Ribeiro, A.M., 2003. A new traversodontid cynodont from the Santa Maria Formation (Ladinian–Carnian) of southern Brazil, with a phylogenetic analysis of Gondwanan traversodontids. Zoological Journal of the Linnean Society 139, 529–545. DOI: 10.1111/j.1096-3642.2003.00096.x

Abdala, F., Sá-Teixeira, A.M., 2004. A traversodontid cynodont of African affinity in the South American Triassic. Palaeontologia Africana 40, 11–22.

Barberena, M.C., 1974. Contribuição ao conhecimento dos cinodontes gonfodontes (Cynodontia, Tritylodontoidea) do Brasil. Tese para obtenção do título de Livre Docente inédita. Ministério de Educação e Cultura, Universidade Federal do Rio Grande do Sul.

Barberena, M.C., 1977. Bioestratigrafia preliminar da Formação Santa Maria. Pesquisas 7, 111–129.

Barberena, M.C., 1978. A huge thecodont from the Triassic of Brazil. Pesquisas 9, 62–75.

Barberena, M.C., 1981a. Novos materiais de *Traversodon stahleckeri* da Formação Santa Maria (Triássico do Rio Grande do Sul). Pesquisas 14, 149–162.

Barberena, M.C., 1981b. Uma nova espécie de *Massetognathus* (*Massetognathus ochagaviae*, sp. nov.) da Formação Santa Maria, Triássico do Rio Grande do Sul. Pesquisas 14, 181–195.

Barberena, M.C., Araújo, D.C., and Lavina, E.L., 1985. Late Permian and Triassic tetrapods of southern Brazil. National Geographic Research 1, 5–20.

Beltrão, R., 1965. Paleontologia de Santa Maria e São Pedro do Sul, Rio Grande do Sul, Brasil*.* Boletim do Instituto de Geociências UFSM 2, 5–114.

Bertoni-Machado, C., Soares, M.B., Kislowski, F.F., Dentzien-Dias, P.C., 2008. Uma peculiar tafocenose controlada por ação biogênica no Triássico Médio do Rio Grande do Sul, Brasil. Revista Pesquisas em Geociências 35(1), 57–69.

Bonaparte, J.F., Soares, M.B., Schultz, C.L., 2006. A new non-mammalian cynodont from the Middle Triassic of southern Brazil and its implications for the ancestry of mammals. Bulletin New Mexico Museum of Natural History & Science 37, 599–607.

Cox, C.B., 1965. New Triassic dicynodonts from South America, their origins and relationships. Philosophical Transactions of the Royal Society of London B 248, 457–516.

Da-Rosa, A.A.S., Schwanke, C., Aurélio, P.L.P., Poitevin, M., Neto, L.W., 2005. Sítio Linha Várzea - uma nova assembléia fossilífera do Triássico Médio do sul do Brasil. Geociências 24, 115–129.

Da-Rosa, A.A.S., Schwanke, C., Cisneros, J.C., Neto, L.W., Aurélio, P.L.P., Poitevin, M., 2004. “Sítio Cortado” - uma nova assembléia fossilífera para o Triássico Médio do sul do Brasil. Revista Brasileira de Paleontologia 7, 289–300.

Dassie, E.C.G., 2014. Tetrápodes triássicos brasileiros: uma investigação envolvendo banco de dados e análise de cluster. [Master's Dissertation]. Universidade de São Paulo (USP). Faculdade de Filosofia, Ciências e Letras de Ribeirão Preto.

Da Silva, L.R., Cabreira, S.F., 2009. Novo achado de *Luangwa sudamericana* Abdala & Teixeira, 2004 do Triássico Médio da Formação Santa Maria, Rio Grande do Sul, Brasil. Paleontologia em Destaque 24, 23–24.

Desojo, J.B., Ezcurra, M., Schultz, C.L., 2011. An unusual new archosauriform from the Middle-Late Triassic of southern Brazil and the monophyly of Doswelliidae. Zoological Journal of the Linnean Society 161, 839–871. DOI: 10.1111/j.1096-3642.2010.00655.x

Dornelles, J.E.F., 1992. *Cerritosaurus binsfeldi* Price 1946 e *Chanaresuchus* sp. (Thecodontia, Proterosuchia, Cerritosauridae) da Formação Santa Maria, Triássico do Rio Grande do Sul, Brasil. MSc Thesis, UFRGS.

Dornelles, J.E.F., 1995. Um tecodonte proterosuchídeo (*Chanaresuchus* sp.) do Triássico do Rio Grande do Sul. Comunicações do Museu de Ciências e Tecnologia UBEA/PUCRS (Série Ciências da Terra) 1, 63–68.

França, M.A., Ferigolo, J., Langer, M.C., 2011. Associated skeletons of a new middle Triassic "Rauisuchia" from Brazil. Naturwissenschaften 98 (5), 389–395. DOI: 10.1007/s00114-011-0782-3

Hanich, D., Bueno, A.O., and Ribeiro, A.M., 2012. Novos materiais de vertebrados da Formação Santa Maria (Triássico Médio, Zona-Assembleia de *Dinodontosaurus*), Município de Dona Francisca, RS, Brasil. In: Reunião Regional da Sociedade Brasileira de Paleontologia (Paleo RS 2012), São João do Polêsine. Livro Resumos Paleo RS 2012, p. 17–17.

Hanich, D., Bertoni, R.S., Abdala, F., and Ribeiro, A.M., 2013. Traversodontidae da Zona Assembleia de *Dinodontosaurus* (Triassico Medio), Dona Francisca, RS, Brasil. In: XXIII Congresso Brasileiro de Paleontologia, 2013, Gramado. Boletim de Resumos, p. 234–235.

Hsiou, A.S., Abdala, F., Arcucci, A., 2002. Novo registro de proterocampsídeo (Reptilia-Archosauriformes) do Triássico médio–superior do Brasil. Revista Brasileira de Paleontologia 3, 48–55.

Huene, F.v., 1935. Lieferung 1. Anomodontia. Die Fossilen Reptilien des Südamerikanischen Gondwanalandes. Ergebnisse der Sauriergrabungen in Südbrasilien 1928/29. C. H. Beck'sche Verlagsbuchhandlung, München 1–82.

Huene, F.v., 1936. Lieferung 2. Cynodontia. Die Fossilen Reptilien des Südamerikanischen Gondwanalandes. Ergebnisse der Sauriergrabungen in Südbrasilien 1928/29. C. H. Beck'sche Verlagsbuchhandlung, München 83–160.

Huene, F.v., 1942. Lieferungen 3/4. Pseudosuchia, Saurischia, Rhynchosauridae und Schlussabschnitt. Die Fossilen Reptilien des Südamerikanischen Gondwanalandes. Ergebnisse der Sauriergrabungen in Südbrasilien 1928/29. C. H. Beck'sche Verlagsbuchhandlung, München 161–332.

Kemp, T.S., 1980. Aspect of the structure and functional anatomy of the Middle Triassic cynodont *Luangwa*. Journal of Zoology 191, 193–239. DOI: 10.1111/j.1469-7998.1980.tb01456.x

Lacerda, M., Mastrantonio, B.M., Fortier, D.C., Schultz, C.L., 2016. New insights on *Prestosuchus chiniquensis* Huene, 1942 (Pseudosuchia, Loricata) based on new specimens from the “Tree Sanga” Outcrop, Chiniquá Region, Rio Grande do Sul, Brazil. PeerJ 4, e1622. DOI: 10.7717/peerj.1622

Langer, M.C., 2004. Basal saurischians. In: Weishampel, D., Dodson, P., Osmólska, H. (Eds.), 2004. The Dinosauria, 2nd edition: Berkeley, University of California Press, p. 25–46.

Langer M.C., Ribeiro, A.M., Schultz, C.L., Ferigolo J., 2007. The continental tetrapod bearing Triassic of south Brazil. Bulletin of the New Mexico Museum of Natural History and Science 41, 201–218.

Liparini, A., Oliveira, T.V., Pretto, F.A., Soares, M.B., Schultz, C.L. 2013. The lower jaw and dentition of the traversodontid *Exaeretodon riograndensis* Abdala, Barberena & Dornelles, from the Brazilian Triassic (Santa Maria 2 Sequence, *Hyperodapedon* Assemblage Zone). Alcheringa 37, 1–7. DOI: 10.1080/03115518.2013.752607

Liu, J., Soares, M.B., Reichel, M., 2008. *Massetognathus* (Cynodontia, Traversodontidae) from the Santa Maria Formation of Brazil. Revista Brasileira de Paleontologia 11, 27–36.

Liu, J., Abdala, F., 2014. Phylogeny and taxonomy of the Traversodontidae. In: Kammerer, C.F., Angielczyk, K.D., Fröbisch, J. (Eds.), 2014. Early evolutionary history of the Synapsida, Springer, 255–279.

Lucas, S.G., 1993. *Barysoma lenzii* (Synapsida: Dicynodontia) from the Middle Triassic of Brazil, a synonym of *Stahleckeria potens*. Journal of Paleontology 67, 318–321. DOI: 10.1017/S0022336000032285

Lucas, S.G., 2002. A new dicynodont from the Triassic of Brazil, and the tetrapod biochronology of the Brazilian Triassic. Bulletin of the New Mexico Museum of Natural History and Science 21, 131–141.

Lucas, S.G. and Harris, S.K., 1996. Taxonomic and biochronological significance of specimens of the Triassic dicynodont *Dinodontosaurus* Romer 1943 in the Tübingen collection. *Paläontologische Zeitschrift* 70, 603–622.

Machado, M., 1992. Estudo taxonômico-populacional dos dicinodontes com presas do RS mediante o emprego da análise discriminante canônica (independente do tamanho). MSc Thesis, UFRGS.

Martinelli, A.G., Soares, M.B., Schwanke, C., 2016. Two new cynodonts (Therapsida) from the Middle-early Late Triassic of Brazil and comments on South American probainognathians. Plos ONE 11(10), e0162945. DOI: 10.1371/journal.pone.0162945

Mastrantonio B.M., Schultz, C.L., Desojo, J.B., Garcia, J.B., 2013. The braincase of *Prestosuchus chiniquensis* (Archosauria: Suchia). In: Nesbitt S., Desojo J.B., Irmis R.B. (Eds.), 2013*.* Anatomy, Phylogeny and Palaeobiology of Early Archosaurs and their Kin. London: The Geological Society of London, 425–440. DOI: 10.1144/SP379.10

Mattar, L.C.B., 1987. Descrição osteólogica do crânio e segunda vértebrata cervical de *Barberenasuchus brasiliensis* Mattar, 1987 (Reptilia, Thecodontia) do Mesotriássico do Rio Grande do Sul, Brasil. Anais da Academia Brasileira de Ciências 61, 319–333.

Nesbitt, S.J., 2011. The early evolution of archosaurs: Relationships and the origin of major clades. Bulletin of the American Museum of Natural History 352, 1–292. DOI: 10.1206/352.1

Pavanatto, A.E.B., Müller, R.T., Da-Rosa, A.A.S., Dias-da-Silva, S., 2016. New information on the postcranial skeleton of *Massetognathus ochagaviae* Barberena, 1981 (Eucynodontia, Traversodontidae), from the Middle Triassic of Southern Brazil. Historical Biology 28(7), 978–989. DOI: 10.1080/08912963.2015.1070148

Peruzzo, C.S., Araújo-Barberena, D.C., 1995. Sobre a ocorrência do gênero *Ischigualastia* Cox, 1962 na Formação Santa Maria, Triássico do Rio Grande do Sul. Anais da Academia Brasileira de Ciências 67, 175–181.

Price, L.I., 1947. Um procolofonídeo do Triássico do Rio Grande do Sul. Boletim da Divisão de Geologia e Paleontologia, DNPM 122, 7–27.

Reichel, M., Schultz, C.L., Soares, M.B., 2009. A new traversodontid cynodont (Therapsida, Eucynodontia) from the Middle Triassic Santa Maria Formation of Rio Grande do Sul, Brazil. Palaeontology 52(1), 229–250. DOI: 10.1111/j.1475-4983.2008.00824.x

Raugust, T., 2014. Descrição osteológica e análise filogenética de um novo material de Rauisuchia (Archosauria, Crurotarsi) da Formação Santa Maria, Triássico Médio Sul-Rio-Grandense, Brasil. PhD Thesis, UFRGS, Porto Alegre.

Romer, A.S. 1967. The Chañares (Argentina) Triassic reptile fauna. III. Two new gomphodonts, *Massetognathus pascuali* and *Massetognathus teruggii*. Breviora 264, 1–25.

Romer, A.S., Price, L.I., 1944. *Stahleckeria lenzii*, a giant Triassic Brazilian dicynodont. Bulletin of the Museum of Comparative Zoology 93, 463–491.

Ruta, M., Botha-Brink J., Mitchell S.A, Benton M.J. 2013. The radiation of cynodonts and the ground plan of mammalian morphological diversity. Proceeding of the Royal Society of London 280, 20131865.

Schultz, C.L., Scherer, C.M.S., Barberena, M.C., 2000. Biostratigraphy of southern Brazilian Middle-Upper Triassic. Revista Brasileira de Geociências 30, 491–494.

Schultz. C.L., Langer, M.C., Montefeltro, F.C., 2016. A new rhynchosaur from south Brazil (Santa Maria Formation) and rhynchosaur diversity patterns across the Middle-Late Triassic boundary. Paläontologische Zeitschrift 90(3), 593–609. DOI: 10.1007/s12542-016-0307-7

Teixeira, A.M.S., 1979. Um novo cinodontes carnívoro (*Probelesodon kitchingi* sp. nov.) do Triássico do Rio Grande do Sul, Brasil. MSc Thesis, UFRGS.

Teixeira, A.M.S., 1982. Um novo cinodonte carnivoro (*Probelesodon kitchingi* sp. nov.) do Triassico do Rio Grande do Sul, Brasil. Comunicações do Museu de Ciências da PUCRS 24, 1–31.

Teixeira, A.M.S., 1987. Novas observações osteológicas e taxonômicas sobre *Massetoganthus ochagaviae* Barberena, 1981 (Reptilia, Cynodontia). Paula-Coutiana 1, 39–49.

Teixeira, A.M.S., 1995. A família Traversodontidae (Therapsida, Cynodontia) no sul do Brasil e suas relações com formas afins no domínio gonduânico. PhD Thesis, UFRGS.

Vega-Dias, C., Maisch, M.W., Schwanke, C., 2005. The taxonomic status of *Stahleckeria impotens* (Therapsida, Dicynodontia): redescription and discussion of its phylogenetic position. Revista Brasileira de Paleontologia 8, 221–228.

Zingano, A.G., Cauduro, A.D., 1959. Afloramentos fossilíferos do Rio Grande do Sul. Boletim Instituto de Ciências Naturais, Ministério de Educação e Cultura, Universidade do Rio Grande do Sul, 8, 1–48.
